# An opioid-sparing protocol with intravenous parecoxib can effectively reduce morphine consumption after simultaneous bilateral total knee arthroplasty

**DOI:** 10.1038/s41598-021-86826-7

**Published:** 2021-04-01

**Authors:** Hsuan-Hsiao Ma, Te-Feng Arthur Chou, Hsin-Yi Wang, Shang-Wen Tsai, Cheng-Fong Chen, Po-Kuei Wu, Wei-Ming Chen

**Affiliations:** 1 Department of Orthopaedics and Traumatology, Taipei Veterans General Hospital, No. 201, Sec 2, Shi-Pai Road, Taipei, 112 Taiwan; 2 Department of Orthopaedics, School of Medicine, National Yang Ming Chiao Tung University, Taipei, Taiwan; 3 Department of Anesthesiology, Taipei Veterans General Hospital, Taipei, Taiwan; 4 Department of Anesthesiology, School of Medicine, National Yang Ming Chiao Tung University, Taipei, Taiwan

**Keywords:** Randomized controlled trials, Outcomes research

## Abstract

Multimodal pain management protocol effectively relieves pain following simultaneous bilateral total knee arthroplasty (SBTKA) but is associated with administration of large amounts of opioids in the perioperative period. In this prospective, randomized, assessor-blinded, single-surgeon clinical trial, the goal was to validate the efficacy of an opioid-sparing protocol for SBTKA with a reduced opioid dose, while achieving similar pain relief with few adverse events. Fifty-six patients who had undergone SBTKA were randomly allocated to receive either an opioid-sparing or opioid-based protocol. The primary outcome parameters were visual analogue scale (VAS) scores at rest, with movement, and cumulative morphine dose, through time. Secondary outcome parameters included drug-related adverse events and range of motion with continuous passive motion device, through time. In the opioid-sparing group, a lower VAS score with movement at postoperative 24 and 72 h was observed compared with the opioid-based group, but the difference did not reach the minimal clinically importance difference. A reduced cumulative morphine dose was noted in the opioid-sparing group at postoperative 24, 48 and 72 h. In conclusion, the opioid-sparing protocol may be used as an alternative modality for pain management following SBTKA. Similar pain relief effects may be achieved utilizing a reduced cumulative opioid dose, with few opioid related adverse events.

## Introduction

Multimodal pain management in total joint arthroplasty has been widely adopted for better pain relief, higher patient satisfaction and faster recovery after surgery^1–3^. However, opioid overdose, addiction and drug-related adverse effects continue to be a challenge for physicians ^4–8^. Therefore, efforts have been made to reduce the need for opioids within multimodal analgesia protocols^9^.

Simultaneous bilateral total knee arthroplasty (SBTKA) is a safe, cost-effective and, when successful, highly satisfying procedure^10,11^. However, postoperative pain management continues to be a challenge for physicians^12–15^. Although it has been validated that pain intensity following SBTKA and unilateral TKA procedures are similar, the narcotics required to relieve the pain during the perioperative period can be 20% higher in patients that underwent SBTKA^15^. Current studies regarding opioid-sparing protocols consisting of intravenous parecoxib assessed patients that had undergone a unilateral TKA procedure^16–19^. As anticipated, intravenous parecoxib was associated with lower pain scores in the postoperative period, compared with the placebo group^16–18^. However, the postoperative cumulative opioid doses in the parecoxib group were relatively higher (28.93–48.86 mg) at postoperative 72 h ^16,18,20^, which did not meet the expectation of an “opioid-sparing” effect.

Therefore, this trial was designed to validate the efficacy of a multimodal opioid-sparing protocol that consisted of intravenous parecoxib for patients that had undergone SBTKA. These patients were considered more likely to benefit from the opioid-sparing protocol that reduced opioid use and thereby possibly minimize adverse events associated with opioids. The purpose of this study was to compare the efficacy of two standardized multimodal pain management protocols in patients who had undergone SBTKA procedures: (1) an opioid-based protocol, or; (2) an opioid-sparing protocol that consisted of multiple doses of intravenous parecoxib.

The hypothesis was that the opioid-sparing protocol will be non-inferior to the opioid-based protocol, in terms of pain scores at various postoperative time points, while the total dosage of opioid rescue for breakthrough pain in the opioid-sparing protocol will be minimal.

## Material and methods

This randomized, assessor-blinded, single-surgeon clinical trial was performed in a single tertiary referral center. From March 20, 2020 through June 20, 2020, patients who were to undergo SBTKA, were randomly allocated to either an opioid-sparing or an opioid-based multimodal pain management protocol immediately prior to the procedure. The study protocol was approved by the ethics committee of Taipei Veterans General Hospital (IRB-2020-02-008C) and was registered on ClinicalTrials.gov (NCT04314505) on March 19, 2020. The study was conducted according to the Declaration of Helsinki and reported based on the statement of Consolidated Standards of Reporting Trials (CONSORT). No funding was provided for this study. Written informed consent was obtained from all patients recruited into this study.

### Patient selection

Inclusion criteria for this study: (1) patients 18 years of age, or older, who had undergone a SBTKA procedure for non-inflammatory conditions such as osteoarthritis or spontaneous osteonecrosis of the knee; (2) a signed, informed consent agreement to participate in the study and to be randomly allocated to either the opioid-sparing or opioid-based protocol.

Exclusion criteria of this study were, patients with history of: (1) total knee arthroplasty procedure for inflammatory arthritis, e.g. rheumatoid arthritis, septic arthritis or gouty arthritis; (2) allergy to any medication used in either protocol; (3) chronic renal impairment (estimated glomerular filtration rate less than 60 ml/min/1.73 m^2^ on at least 2 occasions 90 days apart); (4) severe hepatic impairment (Child–Pugh score ≥ 10 points); (5) chronic opioid-dependent patients (exceeding daily morphine milligram equivalent of 50 mg) at time of recruitment); (6) coronary artery disease (CAD); (7) peptic ulcer disease; (8) substance abuse (e.g. alcohol or narcotics), or; 9) refusal to participate in the study.

### Patient allocation

Patients were randomized using the block method to ensure equal sample size across the groups over time. Randomization was performed using an automated, internet-based randomization system: Random Allocation Software (version 2.0, Microsoft Corporation, WA, USA; URL: https://random-allocation-software.software.informer.com/2.0) in order to generate the random allocation sequence that ensured concealed randomization. The randomization decision was made prior to the surgery. Patients were randomized into two multimodal pain management protocols: opioid-sparing group or opioid-based group. An independent research assistant who collected all postoperative outcome parameters was not aware of the study design or patient allocation.

#### Surgical methods

All procedures were performed under general anesthesia, induced with a bolus dose of fentanyl (3–5 μg/kg) and propofol (1.5–2.5 mg/kg). After loss of eyelash reflex, patients were administered rocuronium (0.6–1 mg/kg) as a neuromuscular blocking agent to facilitate intubation. General anesthesia was maintained with sevoflurane or desflurane throughout to achieve an adequate anesthetic plane. Ventilation was controlled to maintain an end-tidal CO_2_ between 35 and 40 mmHg. Body temperature was maintained between 35.5 and 37.0 °C. At the end of surgery, neuromuscular blockade was antagonized with neostigmine 70 μg/kg and glycopyrrolate 15 μg/kg. Upon completion of the procedure, the anesthetic was discontinued and 100% oxygen was given to the patient until adequate spontaneous ventilation was established, whereupon the endotracheal tube was removed. Patients were then transported to the post-anesthesia care unit until the patient’s general condition was stable.

All SBTKA procedures were performed by a single, high-volume, fellowship-trained surgeon. The surgery was performed under the application of a tourniquet via the mid-vastus approach^21^. Tourniquet pressure was set between 260 and 280 mmHg, dependent upon systolic blood pressure prior to inflation. The maximum pressure was 280 mmHg. The maximum time of tourniquet used on either side was 120 min. Posterior-stabilized, cemented total knee arthroplasty (TKA) (NexGen High Flex, Zimmer Inc., Warsaw, IN USA) was used in all patients. Standard intramedullary alignment tools were used to perform the femoral cuts. An extramedullary guide was used for the proximal tibial cut. After implantation of the prosthesis, the tourniquet was deflated, a periarticular injection and irrigation were completed, a Hemovac drain was inserted (Zimmer, Warsaw, IN, USA) and wound closure performed. Vicryl #1 suture for the joint capsule and Vicryl #2 for the subcutaneous layer (both from Ethicon, New Brunswick, NJ, USA), and 4–0 Polysorb (Medtronic, Minneapolis, MN, USA) for the subcuticular suture, were utilized.

#### Pain control protocol

After a general anesthesia procedure was performed, an experienced anesthesiologist performed an ultrasound-guided, single-injection adductor canal block. The patient was placed supine with the operated leg externally rotated and the knee slightly flexed. The proximal end (where the medial border of sartorius muscle that crosses over the medial border of adductor longus muscle) and distal end (where the femoral artery diverges from the sartorius muscle) of the adductor canal were identified. The needle was advanced from anterior side through the sartorius muscle, toward the midpoint of the canal, where 10 ml of 0.25% bupivacaine was injected. The sealed group allocation envelope was then opened and the patient was assigned to opioid-sparing or opioid-based group. The package contained instructions on the drugs to give before the operation (either patient-controlled analgesia (PCA) device or intravenous parecoxib), data collection sheets and pre-printed prescription labels to be attached to the drug chart.

In the opioid-sparing group, a dose of parecoxib sodium (Dynastat, Pfizer, NY, USA) 40 mg was administered intravenously immediately after the nerve block procedure. After surgery, parecoxib sodium 40 mg was given intravenously every 12 h, for 4 more doses. The patient was informed that an intravenous bolus of rescue morphine (0.1 mg/kg) was available every 4 h if the pain was intolerable.

In the opioid-based group, an intravenous PCA device (Hospira Gemstar PCA Infusion Pump) was applied and initiated right after the nerve block procedure. During the first 72 h postoperatively, analgesia was provided by using IV PCA with morphine (1 mg/mL). The pump was set at a loading dose of 0.05–0.1 mL/kg, an infusion rate of 0.004—0.008 mL/kg/hour, a bolus dose of 0.01–0.02 mL/kg, and a lockout time of 5–12 min. At the anesthesiologist's discretion, an additional antiemetic drug (5 mg of droperidol) was added to the PCA. Following surgery, an anesthesiologist regularly visited the patient and adjusted the infusion rate and bolus dose according to pain intensity and adverse events. The PCA was removed 72 h following surgery.

Prior to wound closure, periarticular injection with 10 ml of 0.25% bupivacaine was administered around the knee joint. All patients had oral paracetamol 500 mg, 4 times/day and celecoxib 200 mg, 2 times/day after surgery.

### Thromboembolism prophylaxis protocol

The study followed a protocol for thromboembolism prophylaxis^22^. Patients with: (1) bilateral TKAs; (2) body mass index (BMI) > 30 kg/m^2^; (3) history of deep vein thrombosis or pulmonary embolism, and (4) varicose vein or stasis dermatitis, were included. This protocol consisted of: (1) an injection of low molecular weight heparin (enoxaparin, Clexane, 2000 IU, 0.2 cc) immediately following surgery, and daily until postoperative day 3, then followed by; (2) low-dose aspirin (Bokey, 100 mg once daily) for 2 weeks, starting at postoperative day 4.

### Postoperative rehabilitation protocol before discharge

A standardized postoperative protocol was provided for all of subjects. Patients were permitted to ambulate and to bear weight without restrictions as soon as they were able. A continuous passive motion (CPM) device for range of motion (ROM) training was utilized four times a day, followed by ice compression over the surgical site for 15 min. CPM was started at 70 degrees and an additional 10 degrees was added at each session, if the patient could tolerate it. The indwelling Foley catheter was removed on postoperative day 1. The Hemovac drain was removed on postoperative day 2. Patients were discharged on postoperative day 4 or 5, as appropriate.

### Outcome measurements

To ensure the success of the assessor-blinded study design, an independent research assistant contacted patients by phone to evaluate pain intensity, at rest and with movement, using VAS scores at five postoperative time points: 6, 12, 24, 48 and 72 h. In determining the efficacy of the opioid-sparing effect the cumulative morphine consumption (mg) in both groups at postoperative 24, 48 and 72 h, was recorded. The maximum ROM, with CPM, that patients could tolerate postoperatively at 24, 48 and 72 h was noted.

All drug related adverse events were noted postoperatively for 2 weeks, including: nausea/vomiting, dry mouth, dizziness, pruritus or skin rashes, constipation, dyspepsia or gastrointestinal bleeding, cardiovascular events such as angina or myocardial infarction, signs of fluid retention, e.g. lower leg edema, decreased urine output or urinary retention, respiratory depression, sedation or cognitive impairment. An overall satisfaction scale (0–100 points) was employed 2 weeks postoperatively, with regards to the efficacy and safety of the pain management protocol.

### Statistical analysis

The study utilized a non-inferiority design. To estimate adequate sample size necessary to achieve clinical significance, the minimal clinically important differences (MCID) for postoperative VAS score after primary TKA was determined to be 22.6^23^. The power was set to 95% to reduce the risk of type II error. Sample size was calculated in R 3.1.6 with the command pwr.t.test from the package pwr for the primary outcome. Assuming a 20% loss or drop off during the study and setting MCID = 22.6, SD = 20.3 (from Danoff et al.^23^), power = 0.95, and a level of significance of p = 0.05, a sample size of 56 patients, with 28 in each group was adequate to determine clinical significance.

Data were entered and analyzed with SPSS software (version 25.0, SPSS Inc., Chicago, IL). Data were represented as mean, range and standard deviation for continuous variables, or number and percentages for categorical variables. Fisher’s exact test was used to compare differences between the two groups for each discrete variable. A value of P < 0.05 was considered statistically significant. The post-hoc power analysis (α = 0.05) of cumulative morphine consumption at different time points was performed using G*Power software (Heinrich-Heine Universität Düsseldorf, Düsseldorf, Germany).

## Results

**Figure 1:**
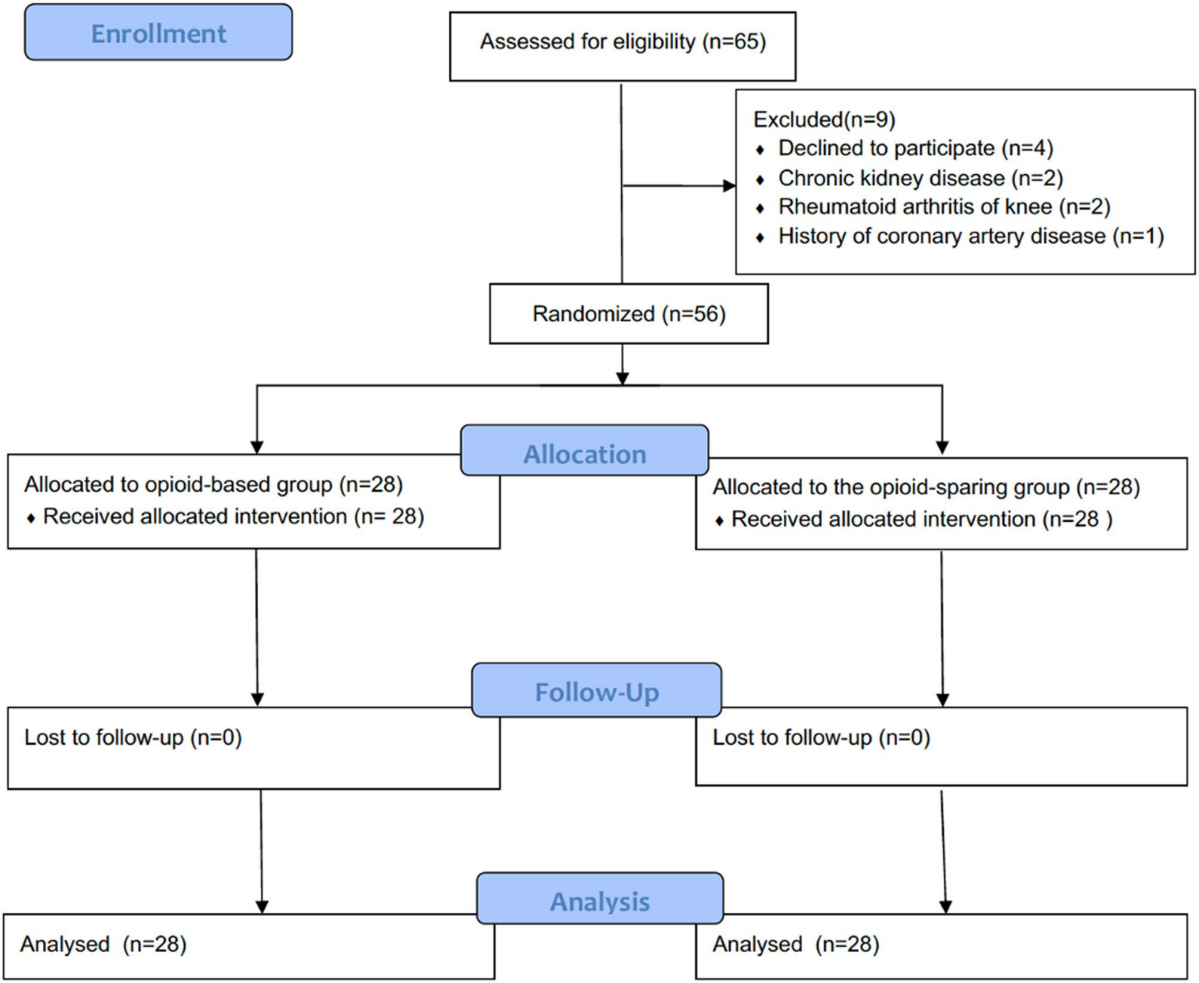
CONSORT flow diagram.

From March 20, 2020 to June 20, 2020, 65 patients who had undergone SBTKA procedures were initially considered for inclusion. Nine patients were excluded for: refusal to participate (N = 4), chronic kidney disease (N = 2), rheumatoid arthritis of the knee (N = 2), and history of CAD (N = 1). The 56 patients who met all inclusion criteria were randomly allocated to either an opioid-sparing or opioid-based protocol (Fig. 1).

Patient demographic data, including age, sex, height, weight, BMI, Charlson comorbidity index (CCI), and American Society of Anesthesiologists score were similar between the two groups. Both groups had similar operation times and similar total blood loss (Table 1).

**Table 1:** Patient demographics.

Baseline Characteristics	Opioid-based group (N = 28)	Opioid-sparing group (N = 28)	*P* values
Age (years)	70.4 ± 6.2	73.1 ± 6.7	0.112
**Sex**			0.737
Female	22 (78.6%)	23 (82.1%)	
Male	6 (21.4%)	5 (17.9%)	
Height (cm)	155.9 ± 8.0	152.0 ± 6.6	0.053
Weight (kg)	68.2 ± 11.9	64.9 ± 10.7	0.273
Body mass index	28.0 ± 4.0	28.0 ± 3.9	0.989
**CCI**			0.249
1	0	1 (3.6%)	
2	7 (25.0%)	3 (10.7%)	
3	8 (28.6%)	8 (28.6%)	
4	7 (25.0%)	13 (46.4%)	
5 +	6 (21.4%)	3 (10.7%)	
**ASA grade**			0.206
1	4 (14.3%)	1 (3.6%)	
2	20 (71.4%)	19 (67.9%)	
3	4 (14.3%)	8 (28.6%)	
Operation time (mins)	114.7 ± 15.1	107.0 ± 22.6	0.143
Tourniquet time of each Procedure (mins)	29.3 ± 4.3	28.9 ± 5.1	0.752
	(range 23–34)	(range 22–35)	
Tourniquet pressure (mmHg)	271.2 ± 5.2	273.1 ± 4.4	0.146
	(range 260–280)	(range 260–280)	
Total blood loss (ml)	743.9 ± 288.1	699.3 ± 226.9	0.523

ASA: American Society of Anesthesiologists; CCI: Charlson comorbidity index.

Postoperatively, patients in the opioid-sparing group demonstrated a lower VAS score with movement, compared with the opioid-based group, at both 24 h (3.68 ± 2.48 vs. 5.18 ± 2.47) and 72 h (3.50 ± 1.80 vs. 4.93 ± 2.23). However, this difference did not reach the MCID of VAS score for the TKA procedure^23^. VAS scores at all other time points were not significantly different between the two groups (Table 2).

**Table 2:** Comparison of clinical outcomes after simultaneous bilateral TKA in both groups.

	Opioid-based group (N = 28)	Opioid-sparing group (N = 28)	*P* values
**VAS score (0–10 points)**			
Post-op 6 h, at rest	3.18 ± 3.06	2.36 ± 2.33	0.263
Post-op 12 h, at rest	3.44 ± 2.04	2.57 ± 1.48	0.074
Post-op 24 h, at rest	1.89 ± 2.04	1.92 ± 1.94	0.947
Post-op 24 h, with movement	5.18 ± 2.47	3.68 ± 2.48	0.027*
Post-op 48 h, at rest	1.36 ± 2.16	1.54 ± 1.69	0.732
Post-op 48 h, with movement	4.50 ± 2.19	3.57 ± 2.18	0.118
Post-op 72 h, at rest	1.64 ± 1.77	1.07 ± 1.44	0.190
Post-op 72 h, with movement	4.93 ± 2.23	3.50 ± 1.80	0.011*
**Cumulative morphine dose (mg)**			
Post-op 24 h	20.99 ± 7.97	3.14 ± 2.62	< 0.001*
Post-op 48 h	35.05 ± 12.46	4.21 ± 3.68	< 0.001*
Post-op 72 h	45.69 ± 16.32	4.75 ± 4.33	< 0.001*
**Continuous passive motion (°)**			
Post-op 24 h	89.07 ± 10.56	93.21 ± 9.83	0.135
Post-op 48 h	104.64 ± 8.39	108.79 ± 8.15	0.066
Post-op 72 h	114.46 ± 6.57	114.92 ± 5.72	0.779
Satisfaction rate (0–100 points)	77.22 ± 13.75	87.46 ± 10.09	0.003*

*p < 0.05.

The cumulative morphine dose was significantly lower postoperatively in the opioid-sparing group, compared with the opioid-based group, at the three time points measured: 24 h (3.14 ± 2.62 mg vs. 20.99 ± 7.97 mg respectively); 48 h (4.21 ± 3.68 mg vs. 35.05 ± 12.46 mg, respectively), and 72 h (4.75 ± 4.33 mg vs. 45.69 ± 16.32 mg, respectively). The post-hoc power analysis revealed that the power was adequate to determine the difference of cumulative morphine dose between the opioid-sparing group and opioid-based group at postoperative 24, 48 and 72 h, with the power values all being 1.0. Patients in the opioid-sparing group had, on average, one dose of rescue morphine, with a cumulative morphine dose of 4.75 ± 4.33 mg at postoperative 72 h. In terms of ROM with CPM, no significant difference at any time point was found (Table 2).

There were 10 drug-related adverse events in the opioid-based group and 5 drug-related adverse events in the opioid-sparing group (Table 3). All events were minor, including nausea/vomiting, dizziness, pruritus or skin rash, and constipation. One patient in the opioid-based group (75-year-old female) developed intolerable pruritus and vomiting and her PCA was removed on postoperative day (POD) 2. Another patient, also in the opioid-based group (70-year-old male), developed cognitive impairment on POD2. A complete workup was performed and no significant systemic disease was noted; drug-related adverse reaction was considered a possible cause. The PCA device was removed and the patient’s normal level of cognitive functioning returned gradually by POD3. No gastrointestinal bleeding or cardiovascular events were observed. Overall satisfaction rate, regarding the efficacy and adverse events of each protocol, was higher in the opioid-sparing group than the opioid-based group (87.46 ± 10.09 vs. 77.22 ± 13.75, respectively).

**Table 3:** Adverse events in both groups.

	Opioid-based group	Opioid-sparing group	*P* values
	(N = 28)	(N = 28)	
**Adverse events**			
Nausea and vomiting	2 (7.1%)	2 (7.1%)	1
Dry mouth	0	0	–
Dizziness	2 (7.1%)	1 (3.6%)	0.55
Pruritus or skin rash	3(10.7%)	0	0.24
Constipation	2 (7.1%)	2 (7.1%)	1
Dyspepsia or gastrointestinal bleeding	0	0	–
Cardiovascular events	0	0	–
Urinary retention	0	0	–
Respiratory depression	0	0	–
Sedation or cognitive impairment	1 (3.6%)	0	1
Total events	10	5	0.26

## Discussion

In this prospective, randomized controlled trial (RCT), we compared the efficacy of two multimodal pain management protocols which consist of preemptive analgesia, regional nerve block, periarticular infiltration analgesia and systemic administration of various medications. The opioid-sparing protocol was found to be capable of reducing perioperative morphine consumption while maintaining satisfactory pain scores that were similar to the opioid-based group. The postoperative VAS score with movement was lower in the opioid-sparing group compared with the opioid based group, at both times measured. At 24 h, the VAS scores for the opioid-sparing were lower than the opioid-based group (3.68 ± 2.48 vs 5.18 ± 2.47, respectively). At 72 h, the scores for the opioid-sparing group were again lower than the opioid-based group (3.50 ± 1.80 vs 4.93 ± 2.23, respectively). In neither case did the difference reach the MCID of VAS scores (2.26) following TKA procedure^23^. The results suggest that the opioid-sparing group was non-inferior to the opioid-based group in terms of pain scores at various postoperative time points. Notably, patients in the opioid-sparing protocol were nearly opioid-free in the perioperative period, with the cumulative morphine dose at postoperative 72 h being 4.75 mg. As noted above, none of the patients in the opioid-sparing group experienced gastrointestinal bleeding or cardiovascular events.

The efficacy of parecoxib for pain relief after total joint arthroplasty has been validated in several studies^16–20^. The efficacy of each multimodal pain management protocol can be difficult to assess. One method is to calculate the MME dosage to assess the efficacy of each protocol. Bian et al. conducted a RCT to compare single-dose, preemptive parecoxib versus placebo treatment in patients who had undergone unilateral TKA. The MME was not significantly different in the two Bain groups with parecoxib vs. placebo (48.86 vs. 51.33 mg, respectively^18^. In two other RCTs comparing multiple doses of parecoxib versus placebo, the opioid-sparing effects appeared to be significant^16,20^. Hubbard observed a dose-dependent opioid-sparing effect of parecoxib after TKA as the MME decreased from approximately 60 mg in the placebo group to approximately 40 mg in the parecoxib group, at postoperative 48 hours^20^. In addition, the PIPFORCE (Postoperative intravenous parecoxib sodium followed by oral celecoxib post total knee arthroplasty in osteoarthritis patients) trial noted the cumulative opioid dose at postoperative 72 h was lower in the parecoxib group compared with the placebo group (28.63 mg vs. 59.57 mg, respectively). The parecoxib vs. placebo difference has been observed up to 6 weeks after surgery, with parecoxib lower than placebo (58.0 mg vs. 180.35 mg, respectively)^16^. None of these studies specifically described the regional analgesia modality in their protocol, which is critical in a multimodal pain management protocol^24^. This could explain the difference in MME reported in these studies^19,25^. Sarridou et al. conducted a RCT to compare the efficacy of the combination of parecoxib and continuous femoral nerve block versus placebo after TKA. Cumulative morphine consumption at postoperative 36 h using this combination was less than 10 mg^19^. Kampitak et al. compared a combination of obturator nerve block and tibial nerve block in addition to a multimodal protocol which consisted of continuous adductor canal block and parecoxib. The cumulative morphine doses at postoperative 48 h in all of the groups were less than 10 mg^25^. All of the above studies included only patients who had undergone unilateral TKA procedures^16,18–20,25^. The population reported in this study consisted of patients that had undergone SBTKA, a procedure which normally requires dosages of narcotics up to 20% higher than unilateral TKA, in order to relieve pain^15^. The opioid-sparing protocol reported here consisted of preemptive analgesia, single-injection nerve block, periarticular infiltration analgesia and systemic administration of medications. In this trial, the cumulative morphine dose at postoperative 72 h in the opioid-sparing group was 4.75 mg, which is much lower than protocols that did not utilize modalities such as nerve block and periarticular infiltration analgesia (MME: 28.93–48.86 mg)^16,18,20^. The results reported here were comparable to protocols consisting of parecoxib plus continuous nerve blocks, in which MME: < 10mg^19,25^. Another advantage of this protocol was the utilization of an ultrasound-guided single-injection adductor canal block, which can be less stressful for the patient, as there are no retained catheters, such as the ones seen with PCA or continuous nerve blocks. In addition, the combination of neuroaxial anesthesia to general anesthesia can lead to reduced risk of mortality, pulmonary complications, gastrointestinal complications, acute renal failure and all-cause infections^26^.

Patients that had undergone SBTKA procedures were selected as the study population because of the established need for, yet the potentially adverse impact of, opioids following total joint arthroplasty^27,28^. In comparison to unilateral TKA, the perioperative opioid dose can increase by up to 20% in a SBTKA procedure^15^. The goal of using parecoxib, an intravenous cyclooxygenase-2 inhibitor as a non-opioid analgesic in the multimodal pain management protocol is to reduce the incidence of opioid-related adverse events, including nausea and vomiting, dizziness, pruritus, constipation, urinary retention, sedation, impaired cognition, respiratory depression and possible death^29^. Most of the adverse events from the study reported here were minor with N = 5 in the opioid-sparing group and N = 10 in the opioid-based group. (As noted above, there was one patient in the opioid-based group who developed cognitive impairment on postoperative day 2.) Cardiovascular events, e.g. myocardial infarction and stroke, and gastrointestinal bleeding are two major adverse events associated with selective cox-2 inhibitors^30^. Although patients with a history of CAD or peptic ulcer disease are not contraindicated for the use of parecoxib, risk was minimized by excluding such patients from this study. The dose and duration of parecoxib (5 doses, within 3 days) was also limited. Possibly as a result of this exclusion, no cardiovascular or gastrointestinal bleeding events were observed.

This study has limitations. First, the required number of subjects was calculated based on a non-inferiority design. The non-inferiority margin was set at 22.6 ± 20.3 mm of VAS score, α = 0.05, 1 − β = 0.95 and a loss or drop off rate of 20%. The sample size was adequate to determine a significant difference between the cumulative morphine doses at different time points but not for drug-related adverse events. Indeed, a number of adverse events in each group were recorded. Due to the relatively small sample size, a conclusion regarding adverse effects cannot be drawn, especially for rare adverse events such as respiratory depression or cognitive impairment. Second, it should be noted that the overlapping prescription of two types of COX-II medication in the opioid-sparing protocol does not lead to additional benefits but rather to a higher risk of adverse effect, such as renal impairment. Therefore, the overlapping prescription of two types of COX-II medication cannot be recommended as the standard of care, before adjusting the prescription of COX-II medication in the opioid-sparing protocol. Third, the access to opioids in the two groups was not equal, which could lead to biased results regarding opioid consumption. Fourth, patients were excluded if there was a history of peptic ulcer disease, chronic renal impairment, severe hepatic impairment or coronary artery disease, based on the potential adverse effect of parecoxib. According to the medication package insert, parecoxib is not contraindicated in patients with the above medical diseases but should be used cautiously [Supplementary file]. The generalizability of this study is limited by these exclusion criteria. Fifth, this study was an assessor-blinded design, in which the patient and the ward personnel were not blinded. Potential biases could have occurred with this setting. Sixth, although patients were not selected based on sex, there were more female than male patients included in this study (80.3%). This percentage is generally a higher than most of the current studies, in which the range is 52.8–65.5%^31–33^. In a review regarding sex differences in pain, there was a trend toward greater post-procedural acute pain in female patients^34^. Therefore, the efficacy of the opioid-sparing protocol with regards to pain scores and cumulative opioid consumption may have been influenced by the relatively high proportion of females. One significant uniform factor was that all the procedures were performed by a single surgeon with identical postoperative care and pain control protocol, except for the interventions using intravenous parecoxib or PCA.

## Conclusion

Based on the finding of up to 85% reduced cumulative opioid consumption when using the opioid-sparing protocol, accompanied by few drug-related adverse events, and the finding that the opioid-sparing protocol was capable of reducing perioperative morphine consumption while maintaining satisfactory pain scores that were similar to the opioid-based group, it is concluded that an opioid-sparing protocol may be an effective alternative to opioid-based protocols.

## Supplementary Information

Supplementary Information.
