# Supplementary material for: An opioid-sparing protocol with intravenous parecoxib can effectively reduce morphine consumption after simultaneous bilateral total knee arthroplasty
Source: Sci Rep. 2021 Apr 1;11:7362. doi: 10.1038/s41598-021-86826-7 (PMC8016913; doi:10.1038/s41598-021-86826-7)
Supplement: Supplementary file 1 — Supplementary Information. [file 41598_2021_86826_MOESM1_ESM.pdf]

Supplement

Title: An opioid-sparing protocol with intravenous parecoxib can effectively reduce morphine consumption after simultaneous bilateral total knee arthroplasty

Author list: Hsuan-Hsiao Ma, Te-Feng Arthur Chou, Hsin-Yi Wang, Shang-Wen Tsai, Cheng-Fong Chen, Po-Kuei Wu, Wei-Ming Chen

Content

Parecoxib package insert: Page 2-Page 73
Celecoxib package insert: Page 74-Page 89

Parecoxib package insert

ANNEX I

SUMMARY OF PRODUCT CHARACTERISTICS

1. NAME OF THE MEDICINAL PRODUCT

Dynastat 40 mg powder for solution for injection

2. QUALITATIVE AND QUANTITATIVE COMPOSITION

Each vial contains 40 mg parecoxib (as 42.36 mg parecoxib sodium). After reconstitution, the concentration of parecoxib is 20 mg/ml. Each 2 ml of reconstituted powder contains 40 mg of parecoxib.

Excipient with known effect
This medicinal product contains less than 1 mmol sodium (23 mg) per dose.

When reconstituted in sodium chloride 9 mg/ml (0.9%) solution, Dynastat contains approximately 0.44 mmol of sodium per vial.

For the full list of excipients, see section 6.1.

3. PHARMACEUTICAL FORM

Powder for solution for injection (powder for injection).
White to off-white powder.

4. CLINICAL PARTICULARS

4.1 Therapeutic indications

For the short-term treatment of postoperative pain in adults.

The decision to prescribe a selective cyclooxygenase-2 (COX-2) inhibitor should be based on an assessment of the individual patient's overall risks (see sections 4.3 and 4.4).

4.2 Posology and method of administration

Posology
The recommended dose is 40 mg administered intravenously (IV) or intramuscularly (IM), followed every 6 to 12 hours by 20 mg or 40 mg as required, not to exceed 80 mg/day.

As the cardiovascular risk of COX-2 specific inhibitors may increase with dose and duration of exposure, the shortest duration possible and the lowest effective daily dose should be used. There is limited clinical experience with Dynastat treatment beyond three days (see section 5.1).

Concomitant use with opioid analgesics
Opioid analgesics can be used concurrently with parecoxib, dosing as described in the paragraph above. In all clinical assessments parecoxib was administered at a fixed time interval whereas the opioids were administered on as needed basis.

Elderly
No dose adjustment is generally necessary in elderly patients (≥65 years). However, for elderly patients weighing less than 50 kg, treatment should be initiated with half the usual recommended dose of Dynastat and reduce the maximum daily dose to 40 mg (see section 5.2).

Hepatic impairment
There is no clinical experience in patients with severe hepatic impairment (Child-Pugh score ≥10), therefore its use is contraindicated in these patients (see sections 4.3 and 5.2). No dosage adjustment is generally necessary in patients with mild hepatic impairment (Child-Pugh score 5-6). Dynastat should be introduced with caution and at half the usual recommended dose in patients with moderate hepatic impairment (Child-Pugh score 7-9) and the maximum daily dose should be reduced to 40 mg.

Renal impairment
In patients with severe renal impairment (creatinine clearance <30 ml/min.) or patients who may be predisposed to fluid retention, parecoxib should be initiated at the lowest recommended dose (20 mg) and the patient's kidney function should be closely monitored (see sections 4.4 and 5.2). On the basis of pharmacokinetics, no dose adjustment is necessary in patients with mild to moderate renal impairment (creatinine clearance of 30-80 ml/min.).

Paediatric population
The safety and efficacy of parecoxib in children under 18 years old have not been established. No data are available. Therefore, parecoxib is not recommended in these patients.

Method of administration
The IV bolus injection may be given rapidly and directly into a vein or into an existing IV line. The IM injection should be given slowly and deeply into the muscle. For instructions on reconstitution of the medicinal product before administration, see section 6.6.

Precipitation may occur when Dynastat is combined in solution with other medicinal products and therefore Dynastat must not be mixed with any other medicinal product, either during reconstitution or injection. In those patients where the same IV line is to be used to inject another medicinal product, the line must be adequately flushed prior to and after Dynastat injection with a solution of known compatibility.

After reconstitution with acceptable solvents, Dynastat may **only** be injected IV or IM, or into IV lines delivering the following:

- sodium chloride 9 mg/ml (0.9%) solution for injection/infusion;
- glucose 50 mg/ml (5%) solution for infusion;
- sodium chloride 4.5 mg/ml (0.45%) and glucose 50 mg/ml (5%) solution for injection/infusion;

  or
- Ringer-Lactate solution for injection.

Injection into an IV line delivering glucose 50 mg/ml (5%) in Ringer-Lactate solution for injection, or other IV fluids not listed above, is **not** recommended as this may cause precipitation from solution.

4.3 Contraindications

Hypersensitivity to the active substance or to any of the excipients listed in section 6.1.

History of previous serious allergic drug reaction of any type, especially cutaneous reactions such as Stevens-Johnson syndrome, drug reaction with eosinophilia and systemic symptoms syndrome (DRESS syndrome), toxic epidermal necrolysis, erythema multiforme or patients with known hypersensitivity to sulfonamides (see sections 4.4 and 4.8).

Active peptic ulceration or gastrointestinal (GI) bleeding.

Patients who have experienced bronchospasm, acute rhinitis, nasal polyps, angioneurotic oedema, urticaria or other allergic-type reactions after taking acetylsalicylic acid or nonsteroidal anti-inflammatory drugs (NSAIDs) including COX-2 inhibitors.

The third trimester of pregnancy and breast-feeding (see sections 4.6 and 5.3).

Severe hepatic impairment (serum albumin <25 g/l or Child-Pugh score ≥ 10).

Inflammatory bowel disease.

Congestive heart failure (NYHA II-IV).

Treatment of post-operative pain following coronary artery bypass graft (CABG) surgery (see sections 4.8 and 5.1).

Established ischaemic heart disease, peripheral arterial disease and/or cerebrovascular disease.

4.4 Special warnings and precautions for use

Dynastat has been studied in dental, orthopaedic, gynaecologic (principally hysterectomy) and coronary artery bypass graft surgery. There is limited experience in other types of surgery, for example gastrointestinal or urological surgery (see section 5.1).

Modes of administration other than IV or IM (e.g. intra-articular, intrathecal) have not been studied and should not be used.

Because of the possibility for increased adverse reactions at higher doses of parecoxib, other COX-2 inhibitors and NSAIDs, patients treated with parecoxib should be reviewed following dose increase and, in the absence of an increase in efficacy, other therapeutic options should be considered (see section 4.2). There is limited clinical experience with Dynastat treatment beyond three days (see section 5.1).

If, during treatment, patients deteriorate in any of the organ system functions described below, appropriate measures should be taken and discontinuation of parecoxib therapy should be considered.

This medicinal product contains less than 1 mmol sodium (23 mg) per dose, and is therefore considered essentially ‘sodium- free’.

Cardiovascular
COX-2 inhibitors have been associated with increased risk of cardiovascular and thrombotic adverse events when taken long term. The exact magnitude of the risk associated with a single dose has not been determined, nor has the exact duration of therapy associated with increased risk.

Patients with significant risk factors for cardiovascular events (e.g. hypertension, hyperlipidaemia, diabetes mellitus, smoking) should only be treated with parecoxib after careful consideration (see section 5.1).

Appropriate measures should be taken and discontinuation of parecoxib therapy should be considered if there is clinical evidence of deterioration in the condition of specific clinical symptoms in these patients. Dynastat has not been studied in cardiovascular revascularization procedures other than coronary artery bypass graft (CABG) procedures. Studies in types of surgery other than CABG procedures included patients with American Society of Anaesthesiology (ASA) Physical Status Class I-III only.

Acetylsalicyclic acid and other NSAIDs
COX-2 inhibitors are not a substitute for acetylsalicylic acid for prophylaxis of cardiovascular thrombo-embolic diseases because of their lack of antiplatelet effects. Therefore, antiplatelet therapies should not be discontinued (see section 5.1). Caution should be exercised when coadministering Dynastat with warfarin and other oral anticoagulants (see section 4.5). The concomitant use of parecoxib with other non- acetylsalicylic acid NSAIDs should be avoided.

Dynastat may mask fever and other signs of inflammation (see section 5.1). In isolated cases, an aggravation of soft tissue infections has been described in connection with the use of NSAIDs and in nonclinical studies with Dynastat (see section 5.3). Caution should be exercised with respect to monitoring the incision for signs of infection in surgical patients receiving Dynastat.

Gastrointestinal
Upper gastrointestinal (GI) complications (perforations, ulcers or bleedings [PUBs]), some of them resulting in fatal outcome, have occurred in patients treated with parecoxib. Caution is advised in the treatment of patients most at risk of developing a gastrointestinal complication with NSAIDs; the elderly, or patients with a prior history of gastrointestinal disease, such as ulceration and GI bleeding, or patients using acetylsalicylic acid concomitantly. The NSAIDs class is also associated with increased GI complications when coadministered with glucocorticoids, selective serotonin reuptake inhibitors, other antiplatelet drugs, other NSAIDs or patients ingesting alcohol. There is further increase in the risk of gastrointestinal adverse effects (gastrointestinal ulceration or other gastrointestinal complications), when parecoxib is taken concomitantly with acetylsalicylic acid (even at low doses).

Skin reactions
Serious skin reactions, including erythema multiforme, exfoliative dermatitis and Stevens-Johnson syndrome (some of them fatal) have been reported through post-marketing surveillance in patients receiving parecoxib. Additionally, fatal reports of toxic epidermal necrolysis have been reported through postmarketing surveillance in patients receiving valdecoxib (the active metabolite of parecoxib) and cannot be ruled out for parecoxib (see section 4.8). DRESS syndrome may occur with parecoxib exposure based on other serious skin reactions reported with celecoxib and valdecoxib exposure. Patients appear to be at highest risk for these reactions early in the course of therapy; the onset of the reaction occurring in the majority of cases within the first month of treatment.

Appropriate measures should be taken by physicians to monitor for any serious skin reactions with therapy, e.g. additional patient consultations. Patients should be advised to immediately report any emergent skin condition to their physician.

Parecoxib should be discontinued at the first appearance of skin rash, mucosal lesions, or any other sign of hypersensitivity. Serious skin reactions are known to occur with NSAIDs including COX-2 selective inhibitors as well as other medicinal products. However, the reported rate of serious skin events appears to be greater for valdecoxib (the active metabolite of parecoxib) as compared to

other COX-2 selective inhibitors. Patients with a history of sulfonamide allergy may be at greater risk of skin reactions (see section 4.3). Patients without a history of sulfonamide allergy may also be at risk for serious skin reactions.

Hypersensitivity
Hypersensitivity reactions (anaphylaxis and angioedema) have been reported in post-marketing experience with valdecoxib and parecoxib (see section 4.8). Some of these reactions have occurred in patients with a history of allergic-type reactions to sulfonamides (see section 4.3). Parecoxib should be discontinued at the first sign of hypersensitivity.

Cases of severe hypotension shortly following parecoxib administration have been reported in postmarketing experience with parecoxib. Some of these cases have occurred without other signs of anaphylaxis. The physician should be prepared to treat severe hypotension.

Fluid retention, oedema, renal
As with other medicinal products known to inhibit prostaglandin synthesis, fluid retention and oedema have been observed in some patients taking parecoxib. Therefore, parecoxib should be used with caution in patients with compromised cardiac function, preexisting oedema, or other conditions predisposing to, or worsened by, fluid retention including those taking diuretic treatment or otherwise at risk of hypovolemia. If there is clinical evidence of deterioration in the condition of these patients, appropriate measures including discontinuation of parecoxib should be taken.

Acute renal failure has been reported through post-marketing surveillance in patients receiving parecoxib (see section 4.8). Since prostaglandin synthesis inhibition may result in deterioration of renal function and fluid retention, caution should be observed when administering Dynastat in patients with impaired renal function (see section 4.2) or hypertension, or in patients with compromised cardiac or hepatic function or other conditions predisposing to fluid retention.

Caution should be used when initiating treatment with Dynastat in patients with dehydration. In this case, it is advisable to rehydrate patients first and then start therapy with Dynastat.

Hypertension
As with all NSAIDs, parecoxib can lead to the onset of new hypertension or worsening of pre-existing hypertension, either of which may contribute to the increased incidence of cardiovascular events. Parecoxib should be used with caution in patients with hypertension. Blood pressure should be monitored closely during the initiation of therapy with parecoxib and throughout the course of therapy. If blood pressure rises significantly, alternative treatment should be considered.

Hepatic impairment
Dynastat should be used with caution in patients with moderate hepatic impairment (Child-Pugh score 7-9) (see section 4.2).

Use with oral anticoagulants
The concomitant use of NSAIDs with oral anticoagulants increases the risk of bleeding. Oral anticoagulants include warfarin/coumarin-type and novel oral anticoagulants (e.g. apixaban, dabigatran, and rivaroxaban) (see section 4.5).

4.5 Interaction with other medicinal products and other forms of interaction

Pharmacodynamic interactions
Anticoagulant therapy should be monitored, particularly during the first few days after initiating Dynastat therapy in patients receiving warfarin or other anticoagulants, since these patients have an increased risk of bleeding complications. Therefore, patients receiving oral anticoagulants should be

closely monitored for their prothrombin time INR, particularly in the first few days when therapy with parecoxib is initiated or the dose of parecoxib is changed (see section 4.4).

Dynastat had no effect on acetylsalicylic acid-mediated inhibition of platelet aggregation or bleeding times. Clinical trials indicate that Dynastat can be given with low dose acetylsalicylic acid (≤ 325 mg). In the submitted studies, as with other NSAIDs, an increased risk of gastrointestinal ulceration or other gastrointestinal complications compared to use of parecoxib alone was shown for concomitant administration of low-dose acetylsalicylic acid (see section 5.1).

Coadministration of parecoxib and heparin did not affect the pharmacodynamics of heparin (activated partial thromboplastin time) compared to heparin alone.

Inhibition of prostaglandins by NSAIDs, including COX-2 inhibitors, may diminish the effect of angiotensin converting enzyme (ACE) inhibitors, angiotensin II antagonists, beta-blockers and diuretics. This interaction should be given consideration in patients receiving parecoxib concomitantly with ACE-inhibitors, angiotensin II antagonists, beta-blockers and diuretics.

In patients who are elderly, volume-depleted (including those on diuretic therapy), or with compromised renal function, coadministration of NSAIDs, including selective COX-2 inhibitors, with ACE inhibitors or Angiotensin-II antagonists, may result in further deterioration of renal function, including possible acute renal failure. These effects are usually reversible.

Therefore, the concomitant administration of these drugs should be done with caution. Patients should be adequately hydrated and the need to monitor the renal function should be assessed at the beginning of the concomitant treatment and periodically thereafter.

Coadministration of NSAIDs and ciclosporin or tacrolimus has been suggested to increase the nephrotoxic effect of ciclosporin and tacrolimus because of NSAID effects on renal prostaglandins. Renal function should be monitored when parecoxib and any of these medicinal products are coadministered.

Dynastat may be coadministered with opioid analgesics. In clinical trials, the daily requirement for PRN opioids was significantly reduced when coadministered with parecoxib.

<u>Effects of other medicinal products on the pharmacokinetics of parecoxib (or its active metabolite valdecoxib)</u>
Parecoxib is rapidly hydrolysed to the active metabolite valdecoxib. In humans, studies demonstrated that valdecoxib metabolism is predominantly mediated via CYP3A4 and 2C9 isozymes.

Plasma exposure (AUC and C_{max}) to valdecoxib was increased (62% and 19%, respectively) when coadministered with fluconazole (predominantly a CYP2C9 inhibitor), indicating that the dose of parecoxib should be reduced in those patients who are receiving fluconazole therapy.

Plasma exposure (AUC and C_{max}) to valdecoxib was increased (38% and 24%, respectively) when coadministered with ketoconazole (CYP3A4 inhibitor), however, a dosage adjustment should not generally be necessary for patients receiving ketoconazole.

The effect of enzyme induction has not been studied. The metabolism of valdecoxib may increase when coadministered with enzyme inducers such as rifampicin, phenytoin, carbamazepine or dexamethasone.

Effect of parecoxib (or its active metabolite valdecoxib) on the pharmacokinetics of other medicinal products
Treatment with valdecoxib (40 mg twice daily for 7 days) produced a 3-fold increase in plasma concentrations of dextromethorphan (CYP2D6 substrate). Therefore, caution should be observed when coadministering Dynastat and medicinal products that are predominantly metabolised by CYP2D6 and which have narrow therapeutic margins (e.g. flecainide, propafenone, metoprolol).

Plasma exposure of omeprazole (CYP 2C19 substrate) 40 mg once daily was increased by 46% following administration of valdecoxib 40 mg twice daily for 7 days, while the plasma exposure to valdecoxib was unaffected. These results indicate that although valdecoxib is not metabolised by CYP2C19, it may be an inhibitor of this isoenzyme. Therefore, caution should be observed when administering Dynastat with medicinal products known to be substrates of CYP2C19 (e.g. phenytoin, diazepam, or imipramine).

In two pharmacokinetic interaction studies in rheumatoid arthritis patients receiving a stable weekly methotrexate dose (5-20 mg/week, as a single oral or intramuscular dose), orally administered valdecoxib (10 mg twice daily or 40 mg twice daily) had little or no effect on the steady-state plasma concentrations of methotrexate. However caution is advised when methotrexate is administered concurrently with NSAIDs, because NSAID administration may result in increased plasma levels of methotrexate. Adequate monitoring of methotrexate-related toxicity should be considered when coadministering parecoxib and methotrexate.

Coadministration of valdecoxib and lithium produced significant decreases in lithium serum clearance (25%) and renal clearance (30%) with a 34% higher serum exposure compared to lithium alone. Lithium serum concentration should be monitored closely when initiating or changing parecoxib therapy in patients receiving lithium.

Coadministration of valdecoxib with glibenclamide (CYP3A4 substrate) did not affect either the pharmacokinetics (exposure) or the pharmacodynamics (blood glucose and insulin levels) of glibenclamide.

Injectable anaesthetics
Coadministration of IV parecoxib 40 mg with propofol (CYP2C9 substrate) or midazolam (CYP3A4 substrate) did not affect either the pharmacokinetics (metabolism and exposure) or the pharmacodynamics (EEG effects, psychomotor tests and waking from sedation) of IV propofol or IV midazolam. Additionally, coadministration of valdecoxib had no clinically significant effect on the hepatic or intestinal CYP 3A4-mediated metabolism of orally administered midazolam. Administration of IV parecoxib 40 mg had no significant effect on the pharmacokinetics of either IV fentanyl or IV alfentanil (CYP3A4 substrates).

Inhalation anaesthetics
No formal interaction studies have been done. In surgery studies in which parecoxib was administered pre-operatively, no evidence of pharmacodynamic interaction was observed in patients receiving parecoxib and the inhalation anaesthetic agents nitrous oxide and isoflurane (see section 5.1).

4.6 Fertility, pregnancy and lactation

Pregnancy
Parecoxib is suspected to cause serious birth defects when administered during the last trimester of pregnancy because as with other medicinal products known to inhibit prostaglandin, it may cause premature closure of the ductus arteriosus or uterine inertia (see sections 4.3, 5.1 and 5.3).

NSAID use during the second or third trimester of pregnancy may cause foetal renal dysfunction which may result in reduction of amniotic fluid volume or oligohydramnios in severe cases. Such effects may occur shortly after treatment initiation and are usually reversible. Pregnant women on NSAIDs should be closely monitored for amniotic fluid volume.

Dynastat is contraindicated in the third trimester of pregnancy (see section 4.3).

There are no adequate data from the use of parecoxib in pregnant women or during labour. However, inhibition of prostaglandin synthesis might adversely affect pregnancy. Data from epidemiological studies suggest an increased risk of miscarriage after use of prostaglandin synthesis inhibitors in early pregnancy. In animals, administration of prostaglandin synthesis inhibitors, including parecoxib, has been shown to result in increased pre- and post-implantation loss and embryo-foetal lethality (see sections 5.1 and 5.3). During the first and second trimester of pregnancy, Dynastat should not be given unless clearly necessary.

Breast-feeding
Administration of a single dose of parecoxib to lactating women following caesarean section resulted in the transfer of a relatively small amount of parecoxib and its active metabolite valdecoxib into human milk, and this resulted in a low relative dose for the infant (approximately 1% of the weight-adjusted maternal dose). Dynastat must not be administered to women who breast-feed (see section 4.3).

Fertility
The use of Dynastat, as with any medicinal product known to inhibit cyclooxygenase/prostaglandin synthesis, is not recommended in women attempting to conceive (see sections 4.3, 5.1 and 5.3).

Based on the mechanism of action, the use of NSAIDs, may delay or prevent rupture of ovarian follicles, which has been associated with reversible infertility in some women. In women who have difficulties conceiving or who are undergoing investigation of infertility, withdrawal of NSAIDs, including Dynastat should be considered.

4.7 Effects on ability to drive and use machines

Patients who experience dizziness, vertigo or somnolence after receiving Dynastat should refrain from driving or operating machines.

4.8 Undesirable effects

Summary of the safety profile
The most common adverse reaction for Dynastat is nausea. The most serious reactions occur uncommonly to rarely, and include cardiovascular events such as myocardial infarction and severe hypotension, as well as hypersensitivity events such as anaphylaxis, angioedema and severe skin reactions. Following coronary artery bypass graft surgery, patients administered Dynastat have a higher risk of adverse reactions such as: cardiovascular/thromboembolic events (including myocardial infarction, stroke/TIA, pulmonary embolus, and deep vein thrombosis; see sections 4.3 and 5.1), deep surgical infections, and sternal wound healing complications.

Tabulated list of adverse reactions
The following adverse reactions were reported for patients who received parecoxib (N=5,402) in 28 placebo-controlled clinical trials. Reports from post-marketing experience have been listed as "frequency not known" because the respective frequencies cannot be estimated from the available data. Within each frequency grouping, adverse reactions are listed using MedDRA terminology and presented in order of decreasing seriousness.

Adverse Drug Reaction Frequency				
Very Common (≥1/10)	*Common (≥1/100 to <1/10)*	*Uncommon (≥1/1000 to <1/100)*	*Rare (≥1/10,000 to <1/1000)*	*Not known*
Infections and infestations				
	Pharyngitis, alveolar osteitis (dry socket)	Abnormal sternal serous wound drainage, wound infection		
Blood and lymphatic system disorders				
	Anaemia postoperative	Thrombocytopenia		
Immune system disorders				
			Anaphylactoid reaction	
Metabolism and nutrition disorders				
	Hypokalaemia	Hyperglycaemia, anorexia		
Psychiatric disorders				
	Agitation, insomnia			
Nervous system disorders				
	Hypoaesthesia, dizziness	Cerebrovascular disorder		
Ear and labyrinth disorders				
		Ear pain		
Cardiac disorders				
		Myocardial infarction, bradycardia		Circulatory collapse, congestive heart failure, tachycardia
Vascular disorders				
	Hypertension, hypotension	Hypertension (aggravated), orthostatic hypotension		
Respiratory, thoracic and mediastinal disorders				
	Respiratory insufficiency	Pulmonary embolism		Dyspnoea
Gastrointestinal disorders				
Nausea	Abdominal pain, vomiting, constipation, dyspepsia, flatulence	Gastroduodenal ulceration, gastrooesophageal reflux disease, dry mouth, gastrointestinal sounds abnormal	Pancreatitis, oesophagitis, oedema mouth (perioral swelling)	

Adverse Drug Reaction Frequency				
Very Common (≥1/10)	*Common* (≥1/100 to <1/10)	*Uncommon* (≥1/1000 to <1/100)	*Rare* (≥1/10,000 to <1/1000)	*Not known*
Skin and subcutaneous tissue disorders				
	Pruritus, hyperhidrosis	Ecchymosis, rash, urticaria		Stevens-Johnson syndrome, erythema multiforme, exfoliative dermatitis
Musculoskeletal and connective tissue disorders				
	Back pain	Arthralgia		
Renal and urinary disorders				
	Oliguria		Renal failure acute	Renal failure,
General disorders and administration site conditions				
	Oedema peripheral	Asthenia, injection site pain, injection site reaction		Hypersensitivity reactions including anaphylaxis and angioedema
Investigations				
	Blood creatinine increased	Blood CPK increased, blood LDH increased, SGOT increased, SGPT increased, BUN increased.		
Injury, poisoning and procedural complications				
		Post procedural complication (skin)		

Description of selected adverse reactions
In post-marketing experience, toxic epidermal necrolysis has been reported in association with the use of valdecoxib, and cannot be ruled out for parecoxib (see section 4.4). In addition, the following rare, serious adverse reactions have been reported in association with the use of NSAIDs and cannot be ruled out for Dynastat: bronchospasm and hepatitis.

Reporting of suspected adverse reactions
Reporting suspected adverse reactions after authorisation of the medicinal product is important. It allows continued monitoring of the benefit/risk balance of the medicinal product. Healthcare professionals are asked to report any suspected adverse reactions via the national reporting system listed in Appendix V.

4.9 Overdose

Reporting of overdose with parecoxib has been associated with adverse reactions which have also been described with recommended doses of parecoxib.

In case of overdose, patients should be managed by symptomatic and supportive care. Valdecoxib is not removed by haemodialysis. Diuresis or alkalisation of urine may not be useful due to high protein binding of valdecoxib.

5. PHARMACOLOGICAL PROPERTIES

5.1 Pharmacodynamic properties

Pharmacotherapeutic group: Antiinflammatory and antirheumatic products, Coxibs, ATC code: M01AH04

Parecoxib is a prodrug of valdecoxib. Valdecoxib is a selective COX-2 inhibitor within the clinical dose range. Cyclooxygenase is responsible for generation of prostaglandins. Two isoforms, COX-1 and COX-2, have been identified. COX-2 is the isoform of the enzyme that has been shown to be induced by pro-inflammatory stimuli and has been postulated to be primarily responsible for the synthesis of prostanoid mediators of pain, inflammation, and fever. COX-2 is also involved in ovulation, implantation and closure of the ductus arteriosus, regulation of renal function, and central nervous system functions (fever induction, pain perception and cognitive function). It may also play a role in ulcer healing. COX-2 has been identified in tissue around gastric ulcers in man but its relevance to ulcer healing has not been established.

The difference in antiplatelet activity between some COX-1 inhibiting NSAIDs and COX-2 selective inhibitors may be of clinical significance in patients at risk of thrombo-embolic reactions. COX-2 selective inhibitors reduce the formation of systemic (and therefore possibly endothelial) prostacyclin without affecting platelet thromboxane. The clinical relevance of these observations has not been established.

Parecoxib has been used in a range of major and minor surgeries. The efficacy of Dynastat was established in studies of dental, gynaecologic (hysterectomy), orthopaedic (knee and hip replacement), and coronary artery bypass graft surgical pain. The first perceptible analgesic effect occurred in 7-13 minutes, with clinically meaningful analgesia demonstrated in 23-39 minutes and a peak effect within 2 hours following administration of single doses of 40 mg IV or IM Dynastat. The magnitude of analgesic effect of the 40 mg dose was comparable with that of ketorolac 60 mg IM or ketorolac 30 mg IV. After a single dose, the duration of analgesia was dose and clinical pain model dependent, and ranged from 6 to greater than 12 hours.

<u>Use of parecoxib beyond 3 days</u>
Most trials were designed for dosing of parecoxib up to 3 days. Data from 3 randomised placebo-controlled trials, where the protocols allowed treatment of parecoxib for >3 days was pooled and analysed. In the pooled analysis of 676 patients, 318 received placebo and 358 received parecoxib. Of the patients treated with parecoxib, 317 patients received parecoxib for up to 4 days, 32 patients for up to 5 days, while only 8 patients were treated for up to 6 days and 1 patient for 7 or more days. Of the patients treated with placebo, 270 patients received placebo for up to 4 days, 43 patients for up to 5 days, while only 3 patients were treated for up to 6 days and 2 patients for 7 or more days. Both groups had similar demographics. The mean (SD) duration of treatment was 4.1 (0.4) days for parecoxib and 4.2 (0.5) days for placebo, the range was 4-7 days for parecoxib and 4-9 days for placebo. The occurrence of adverse events in patients receiving parecoxib for 4-7 days (median duration 4 days) was low after treatment Day 3 and similar to placebo.

<u>Opioid-sparing effects</u>
In a placebo-controlled, orthopedic and general surgery study (n =1050), patients received Dynastat at an initial parenteral dose of 40 mg IV followed by 20 mg twice daily for a minimum of 72 hours in addition to receiving standard care including supplemental patient controlled opioids. The reduction in opioid use with Dynastat treatment on Days 2 and 3 was 7.2 mg and 2.8 mg (37% and 28% respectively).This reduction in opioid use was accompanied by significant reductions in patient-reported opioid symptom distress. Added pain relief compared to opioids alone was shown.

Additional studies in other surgical settings provided similar observations. There are no data indicating less overall adverse events associated with the use of parecoxib compared to placebo when used in conjunction with opioids.

Gastrointestinal studies

In short-term studies (7 days), the incidence of endoscopically observed gastroduodenal ulcers or erosions in healthy young and elderly (≥ 65 years) subjects administered Dynastat (5-21%), although higher than placebo (5-12%), was statistically significantly lower than the incidence observed with NSAIDs (66-90%).

CABG post-operative safety studies

In addition to routine adverse event reporting, pre-specified event categories, adjudicated by an independent expert committee, were examined in two placebo-controlled safety studies in which patients received parecoxib for at least 3 days and then were transitioned to oral valdecoxib for a total duration of 10-14 days. All patients received standard of care analgesia during treatment. Patients received low-dose acetylsalicylic acid prior to randomization and throughout the two CABG surgery studies.

The first CABG surgery study evaluated patients treated with IV parecoxib 40 mg bid for a minimum of 3 days, followed by treatment with valdecoxib 40 mg bid (parecoxib/valdecoxib group) (n=311) or placebo/placebo (n=151) in a 14-day, double-blind placebo-controlled study. Nine pre-specified adverse event categories were evaluated (cardiovascular thromboembolic events, pericarditis, new onset or exacerbation of congestive heart failure, renal failure/dysfunction, upper GI ulcer complications, major non-GI bleeds, infections, non-infectious pulmonary complications, and death). There was a significantly ($p<0.05$) greater incidence of cardiovascular/thromboembolic events (myocardial infarction, ischemia, cerebrovascular accident, deep vein thrombosis and pulmonary embolism) detected in the parecoxib/valdecoxib treatment group compared to the placebo/placebo treatment group for the IV dosing period (2.2% and 0.0% respectively) and over the entire study period (4.8% and 1.3% respectively). Surgical wound complications (most involving the sternal wound) were observed at an increased rate with parecoxib/valdecoxib treatment.

In the second CABG surgery study, four pre-specified event categories were evaluated (cardiovascular/thromboembolic; renal dysfunction/renal failure; upper GI ulcer/bleeding; surgical wound complication). Patients were randomized within 24-hours post-CABG surgery to: parecoxib initial dose of 40 mg IV, then 20 mg IV Q12H for a minimum of 3 days followed by valdecoxib PO (20 mg Q12H) (n=544) for the remainder of a 10 day treatment period; placebo IV followed by valdecoxib PO (n=544); or placebo IV followed by placebo PO (n=548). A significantly ($p=0.033$) greater incidence of events in the cardiovascular/thromboembolic category was detected in the parecoxib/valdecoxib treatment group (2.0%) compared to the placebo/placebo treatment group (0.5%). Placebo/valdecoxib treatment was also associated with a higher incidence of CV thromboembolic events versus placebo treatment, but this difference did not reach statistical significance. Three of the six cardiovascular thromboembolic events in the placebo/valdecoxib treatment group occurred during the placebo treatment period; these patients did not receive valdecoxib. Pre-specified events that occurred with the highest incidence in all three treatment groups involved the category of surgical wound complications, including deep surgical infections and sternal wound healing events.

There were no significant differences between active treatments and placebo for any of the other pre-specified event categories (renal dysfunction/failure, upper GI ulcer complications or surgical wound complications).

General surgery

In a large (N=1050) major orthopedic/general surgery trial, patients received an initial dose of parecoxib 40 mg IV, then 20 mg IV Q12H for a minimum of 3 days followed by valdecoxib PO (20 mg Q12H) (n=525) for the remainder of a 10 day treatment period, or placebo IV followed by

placebo PO (n=525). There were no significant differences in the overall safety profile, including the four pre-specified event categories described above for the second CABG surgery study, for parecoxib/valdecoxib compared to placebo treatment in these post-surgical patients.

Platelet studies
In a series of small, multiple dose studies in healthy young and elderly subjects, Dynastat 20 mg or 40 mg twice daily had no effect on platelet aggregation or bleeding compared to placebo. In young subjects, Dynastat 40 mg twice daily had no clinically significant effect on acetylsalicylic acid-mediated inhibition of platelet function (see section 4.5).

5.2 Pharmacokinetic properties

Following IV or IM injection, parecoxib is rapidly converted to valdecoxib, the pharmacologically active substance, by enzymatic hydrolysis in the liver.

Absorption
Exposure of valdecoxib following single doses of Dynastat, as measured by both the area under the plasma concentration vs. time curve (AUC) and peak concentration (C_{max}), is approximately linear in the range of clinical doses. AUC and C_{max} following twice daily administration is linear up to 50 mg IV and 20 mg IM. Steady state plasma concentrations of valdecoxib were reached within 4 days with twice daily dosing.

Following single IV and IM doses of parecoxib 20 mg, C_{max} of valdecoxib is achieved in approximately 30 minutes and approximately 1 hour, respectively. Exposure to valdecoxib was similar in terms of AUC and C_{max} following IV and IM administration. Exposure to parecoxib was similar after IV or IM administration in terms of AUC. Average C_{max} of parecoxib after IM dosing was lower compared to bolus IV dosing, which is attributed to slower extravascular absorption after IM administration. These decreases were not considered clinically important since C_{max} of valdecoxib is comparable after IM and IV parecoxib administration.

Distribution
The volume of distribution of valdecoxib after its IV administration is approximately 55 litres. Plasma protein binding is approximately 98% over the concentration range achieved with the highest recommended dose, 80 mg/day. Valdecoxib, but not parecoxib, is extensively partitioned into erythrocytes.

Biotransformation
Parecoxib is rapidly and almost completely converted to valdecoxib and propionic acid in vivo with a plasma half-life of approximately 22 minutes. Elimination of valdecoxib is by extensive hepatic metabolism involving multiple pathways, including cytochrome P 450 (CYP) 3A4 and CYP2C9 isoenzymes and glucuronidation (about 20%) of the sulfonamide moiety. A hydroxylated metabolite of valdecoxib (via the CYP pathway) has been identified in human plasma that is active as a COX-2 inhibitor. It represents approximately 10% of the concentration of valdecoxib; because of this metabolite’s low concentration, it is not expected to contribute a significant clinical effect after administration of therapeutic doses of parecoxib.

Elimination
Valdecoxib is eliminated via hepatic metabolism with less than 5% unchanged valdecoxib recovered in the urine. No unchanged parecoxib is detected in urine and only trace amounts in the faeces. About 70% of the dose is excreted in the urine as inactive metabolites. Plasma clearance (CL_p) for valdecoxib is about 6 l/hr. After IV or IM dosing of parecoxib, the elimination half-life ($t_{1/2}$) of valdecoxib is about 8 hours.

Elderly
Dynastat has been administered to 335 elderly patients (65-96 years of age) in pharmacokinetic and therapeutic trials. In healthy elderly subjects, the apparent oral clearance of valdecoxib was reduced, resulting in an approximately 40% higher plasma exposure of valdecoxib compared to healthy young subjects. When adjusted for body weight, steady state plasma exposure of valdecoxib was 16% higher in elderly females compared to elderly males (see section 4.2).

Renal impairment
In patients with varying degrees of renal impairment administered 20 mg IV Dynastat, parecoxib was rapidly cleared from plasma. Because renal elimination of valdecoxib is not important to its disposition, no changes in valdecoxib clearance were found even in patients with severe renal impairment or in patients undergoing dialysis (see section 4.2).

Hepatic impairment
Moderate hepatic impairment did not result in a reduced rate or extent of parecoxib conversion to valdecoxib. In patients with moderate hepatic impairment (Child-Pugh score 7-9), treatment should be initiated with half the usual recommended dose of Dynastat and the maximum daily dose should be reduced to 40 mg since valdecoxib exposures were more than doubled (130%) in these patients. Patients with severe hepatic impairment have not been studied and therefore the use of Dynastat in patients with severe hepatic impairment is not recommended (see sections 4.2 and 4.3).

5.3 Preclinical safety data

Non-clinical data reveal no special hazard for humans based on conventional studies of safety pharmacology or repeated dose toxicity at 2-fold the maximum human exposure to parecoxib. However, in the repeated dose toxicity studies in dogs and rats, the systemic exposures to valdecoxib (the active metabolite of parecoxib) were approximately 0.8-fold the systemic exposure in elderly human subjects at the maximum recommended therapeutic dose of 80 mg daily. Higher doses were associated with aggravation and delayed healing of skin infections, an effect probably associated with COX-2 inhibition.

In reproduction toxicity tests, the incidence of post-implantation losses, resorptions and foetal body weight retardation occurred at doses not producing maternal toxicity in the rabbit studies. No effects of parecoxib on male or female fertilities were found in rats.

The effects of parecoxib have not been evaluated in late pregnancy or in the pre- and postnatal period.

Parecoxib administered intravenously to lactating rats as a single dose showed concentrations of parecoxib, valdecoxib and a valdecoxib active metabolite in milk similar to that of maternal plasma.

The carcinogenic potential of parecoxib has not been evaluated.

6. PHARMACEUTICAL PARTICULARS

6.1 List of excipients

Disodium hydrogen phosphate
Phosphoric acid and/or sodium hydroxide (for pH adjustment).

6.2 Incompatibilities

This medicinal product must not be mixed with other medicinal products except for those mentioned in section 6.6.

Dynastat and opioids should not be administered together in the same syringe.

Use of Ringer-Lactate solution for injection or glucose 50 mg/ml (5%) in Ringer Lactate solution for injection for reconstitution will cause the parecoxib to precipitate from solution and therefore is **not** recommended.

Use of water for injection is **not** recommended, as the resulting solution is not isotonic.

Dynastat should not be injected into an IV line delivering any other medicinal product. The IV line must be adequately flushed prior to and after Dynastat injection with a solution of known compatibility (see section 6.6).

Injection into an IV line delivering glucose 50 mg/ml (5%) in Ringer-Lactate solution for injection, or other IV fluids not listed in section 6.6, is not recommended as this may cause precipitation from solution.

6.3 Shelf life

The shelf life of the unreconstituted product is 3 years.

Chemical and physical in-use stability of the reconstituted solution, which should not be refrigerated or frozen, have been demonstrated for up to 24 hours at 25°C. Thus, 24 hours should be considered the maximum shelf life of the reconstituted product. However, due to the importance of microbiological infection risk for injectable products, the reconstituted solution should be used immediately unless reconstitution has taken place in controlled and validated aseptic conditions. Unless such requirements are met, in-storage times and conditions prior to use are the responsibility of the user, and would not normally be longer than 12 hours at 25°C.

6.4 Special precautions for storage

This medicinal product does not require any special storage conditions prior to reconstitution.

For storage conditions of the reconstituted medicinal product see section 6.3.

6.5 Nature and contents of container

Type I colourless glass vials (5 ml) with a butyl rubber stopper, sealed with a purple polypropylene flip-off cap on the aluminium overseal.

Dynastat is available in packs containing 10 vials.

6.6 Special precautions for disposal and other handling.

Dynastat must be reconstituted before use. Dynastat is preservative free. Aseptic technique is required for its preparation.

Reconstitution solvents
Acceptable solvents for reconstitution of Dynastat are:

- sodium chloride 9 mg/ml (0.9%) solution for injection/infusion
- glucose 50 mg/ml (5%) solution for infusion
- sodium chloride 4.5 mg/ml (0.45%) and glucose 50 mg/ml (5%) solution for injection/infusion

Reconstitution process
Use aseptic technique to reconstitute lyophilised parecoxib (as parecoxib).
Remove the purple flip-off cap to expose the central portion of the rubber stopper of the 40 mg parecoxib vial. Withdraw, with a sterile needle and syringe, 2 ml of an acceptable solvent and insert the needle through the central portion of the rubber stopper transferring the solvent into the 40 mg vial. Dissolve the powder completely using a gentle swirling motion and inspect the reconstituted product before use. The entire contents of the vial should be withdrawn for a single administration.

After reconstitution, the liquid should be a clear solution. Dynastat should be inspected visually for particulate matter and discoloration prior to administration. The solution should not be used if discolored or cloudy, or if particulate matter is observed. Dynastat should be administered within 24 hours of reconstitution (see section 6.3), or discarded.

The reconstituted product is isotonic.

IV line solution compatibility
After reconstitution with acceptable solvents, Dynastat may **only** be injected IV or IM, or into IV lines delivering:

- sodium chloride 9 mg/ml (0.9%) solution for injection/infusion;
- glucose 50 mg/ml (5%) solution for infusion;
- sodium chloride 4.5 mg/ml (0.45%) and glucose 50 mg/ml (5%) solution for injection/infusion;
  or
- Ringer-Lactate solution for injection.

For single use only. Any unused medicinal product or waste material should be disposed of in accordance with local requirements.

7. MARKETING AUTHORISATION HOLDER

Pfizer Europe MA EEIG
Boulevard de la Plaine 17
1050 Bruxelles
Belgium

8. MARKETING AUTHORISATION NUMBER(S)

EU/1/02/209/005

9. DATE OF FIRST AUTHORISATION/RENEWAL OF THE AUTHORISATION

Date of first authorisation: 22 March 2002
Date of latest renewal: 24 January 2012

10. DATE OF REVISION OF THE TEXT

Detailed information on this medicinal product is available on the website of the European Medicines Agency http://www.ema.europa.eu.

1. NAME OF THE MEDICINAL PRODUCT

Dynastat 40 mg powder and solvent for solution for injection

2. QUALITATIVE AND QUANTITATIVE COMPOSITION

Powder vial: Each vial contains 40 mg parecoxib (as 42.36 mg parecoxib sodium). After reconstitution, the concentration of parecoxib is 20 mg/ml. Each 2 ml of reconstituted powder contains 40 mg of parecoxib.

Excipient with known effect
This medicinal product contains less than 1 mmol sodium (23 mg) per dose.

When reconstituted in sodium chloride 9 mg/ml (0.9%) solution, Dynastat contains approximately 0.44 mmol of sodium per vial.

For the full list of excipients, see section 6.1.

3. PHARMACEUTICAL FORM

Powder and solvent for solution for injection (powder for injection).
White to off-white powder.

Solvent: clear and colourless solution.

4. CLINICAL PARTICULARS

4.1 Therapeutic indications

For the short-term treatment of postoperative pain in adults.

The decision to prescribe a selective cyclooxygenase-2 (COX-2) inhibitor should be based on an assessment of the individual patient's overall risks (see sections 4.3 and 4.4).

4.2 Posology and method of administration

Posology
The recommended dose is 40 mg administered intravenously (IV) or intramuscularly (IM), followed every 6 to 12 hours by 20 mg or 40 mg as required, not to exceed 80 mg/day.

As the cardiovascular risk of COX-2 specific inhibitors may increase with dose and duration of exposure, the shortest duration possible and the lowest effective daily dose should be used. There is limited clinical experience with Dynastat treatment beyond three days (see section 5.1).

Concomitant use with opioid analgesics
Opioid analgesics can be used concurrently with parecoxib, dosing as described in the paragraph above. In all clinical assessments parecoxib was administered at a fixed time interval whereas the opioids were administered on as needed basis.

Elderly
No dose adjustment is generally necessary in elderly patients (≥65 years). However, for elderly patients weighing less than 50 kg, treatment should be initiated with half the usual recommended dose of Dynastat and reduce the maximum daily dose to 40 mg (see section 5.2).

Hepatic impairment
There is no clinical experience in patients with severe hepatic impairment (Child-Pugh score ≥10), therefore its use is contraindicated in these patients (see sections 4.3 and 5.2). No dosage adjustment is generally necessary in patients with mild hepatic impairment (Child-Pugh score 5-6). Dynastat should be introduced with caution and at half the usual recommended dose in patients with moderate hepatic impairment (Child-Pugh score 7-9) and the maximum daily dose should be reduced to 40 mg.

Renal impairment
In patients with severe renal impairment (creatinine clearance <30 ml/min.) or patients who may be predisposed to fluid retention, parecoxib should be initiated at the lowest recommended dose (20 mg) and the patient's kidney function should be closely monitored (see sections 4.4 and 5.2). On the basis of pharmacokinetics, no dose adjustment is necessary in patients with mild to moderate renal impairment (creatinine clearance of 30-80 ml/min.).

Paediatric population
The safety and efficacy of parecoxib in children under 18 years old have not been established. No data are available. Therefore, parecoxib is not recommended in these patients.

Method of administration
The IV bolus injection may be given rapidly and directly into a vein or into an existing IV line. The IM injection should be given slowly and deeply into the muscle. For instructions on reconstitution of the medicinal product before administration, see section 6.6.

Precipitation may occur when Dynastat is combined in solution with other medicinal products and therefore Dynastat must not be mixed with any other medicinal product, either during reconstitution or injection. In those patients where the same IV line is to be used to inject another medicinal product, the line must be adequately flushed prior to and after Dynastat injection with a solution of known compatibility.

After reconstitution with acceptable solvents, Dynastat may **only** be injected IV or IM, or into IV lines delivering the following:

- sodium chloride 9 mg/ml (0.9%) solution for injection/infusion;
- glucose 50 mg/ml (5%) solution for infusion;
- sodium chloride 4.5 mg/ml (0.45%) and glucose 50 mg/ml (5%) solution for injection/infusion; or
- Ringer-Lactate solution for injection.

Injection into an IV line delivering glucose 50 mg/ml (5%) in Ringer-Lactate solution for injection, or other IV fluids not listed above, is **not** recommended as this may cause precipitation from solution.

4.3 Contraindications

Hypersensitivity to the active substance or to any of the excipients listed in section 6.1.

History of previous serious allergic drug reaction of any type, especially cutaneous reactions such as Stevens-Johnson syndrome, drug reaction with eosinophilia and systemic symptoms syndrome (DRESS syndrome), toxic epidermal necrolysis, erythema multiforme or patients with known hypersensitivity to sulfonamides (see sections 4.4 and 4.8).

Active peptic ulceration or gastrointestinal (GI) bleeding.

Patients who have experienced bronchospasm, acute rhinitis, nasal polyps, angioneurotic oedema, urticaria or other allergic-type reactions after taking acetylsalicylic acid or nonsteroidal anti-inflammatory drugs (NSAIDs) including COX-2 inhibitors.

The third trimester of pregnancy and breast-feeding (see sections 4.6 and 5.3).

Severe hepatic impairment (serum albumin <25 g/l or Child-Pugh score ≥10).

Inflammatory bowel disease.

Congestive heart failure (NYHA II-IV).

Treatment of post-operative pain following coronary artery bypass graft (CABG) surgery (see sections 4.8 and 5.1).

Established ischaemic heart disease, peripheral arterial disease and/or cerebrovascular disease.

4.4 Special warnings and precautions for use

Dynastat has been studied in dental, orthopaedic, gynaecologic (principally hysterectomy) and coronary artery bypass graft surgery. There is limited experience in other types of surgery, for example gastrointestinal or urological surgery (see section 5.1).

Modes of administration other than IV or IM (e.g. intra-articular, intrathecal) have not been studied and should not be used.

Because of the possibility for increased adverse reactions at higher doses of parecoxib, other COX-2 inhibitors and NSAIDs, patients treated with parecoxib should be reviewed following dose increase and, in the absence of an increase in efficacy, other therapeutic options should be considered (see section 4.2). There is limited clinical experience with Dynastat treatment beyond three days (see section 5.1).

If, during treatment, patients deteriorate in any of the organ system functions described below, appropriate measures should be taken and discontinuation of parecoxib therapy should be considered.

This medicinal product contains less than 1 mmol sodium (23 mg) per dose, and is therefore considered essentially ‘sodium- free’.

Cardiovascular
COX-2 inhibitors have been associated with increased risk of cardiovascular and thrombotic adverse events when taken long term. The exact magnitude of the risk associated with a single dose has not been determined, nor has the exact duration of therapy associated with increased risk.

Patients with significant risk factors for cardiovascular events (e.g. hypertension, hyperlipidaemia, diabetes mellitus, smoking) should only be treated with parecoxib after careful consideration (see section 5.1).

Appropriate measures should be taken and discontinuation of parecoxib therapy should be considered if there is clinical evidence of deterioration in the condition of specific clinical symptoms in these patients. Dynastat has not been studied in cardiovascular revascularization procedures other than coronary artery bypass graft (CABG) procedures. Studies in types of surgery other than CABG procedures included patients with American Society of Anaesthesiology (ASA) Physical Status Class I-III only.

Acetylsalicyclic acid and other NSAIDs
COX-2 inhibitors are not a substitute for acetylsalicylic acid for prophylaxis of cardiovascular thrombo-embolic diseases because of their lack of antiplatelet effects. Therefore, antiplatelet therapies should not be discontinued (see section 5.1). Caution should be exercised when coadministering Dynastat with warfarin and other oral anticoagulants (see section 4.5). The concomitant use of parecoxib with other non- acetylsalicylic acid NSAIDs should be avoided.

Dynastat may mask fever and other signs of inflammation (see section 5.1). In isolated cases, an aggravation of soft tissue infections has been described in connection with the use of NSAIDs and in nonclinical studies with Dynastat (see section 5.3). Caution should be exercised with respect to monitoring the incision for signs of infection in surgical patients receiving Dynastat.

Gastrointestinal
Upper gastrointestinal (GI) complications (perforations, ulcers or bleedings [PUBs]), some of them resulting in fatal outcome, have occurred in patients treated with parecoxib. Caution is advised in the treatment of patients most at risk of developing a gastrointestinal complication with NSAIDs; the elderly, or patients with a prior history of gastrointestinal disease, such as ulceration and GI bleeding, or patients using acetylsalicylic acid concomitantly. The NSAIDs class is also associated with increased GI complications when coadministered with glucocorticoids, selective serotonin reuptake inhibitors, other antiplatelet drugs, other NSAIDs or patients ingesting alcohol. There is further increase in the risk of gastrointestinal adverse effects (gastrointestinal ulceration or other gastrointestinal complications), when parecoxib is taken concomitantly with acetylsalicylic acid (even at low doses).

Skin reactions
Serious skin reactions, including erythema multiforme, exfoliative dermatitis and Stevens-Johnson syndrome (some of them fatal) have been reported through post-marketing surveillance in patients receiving parecoxib. Additionally, fatal reports of toxic epidermal necrolysis have been reported through postmarketing surveillance in patients receiving valdecoxib (the active metabolite of parecoxib) and cannot be ruled out for parecoxib (see section 4.8). DRESS syndrome may occur with parecoxib exposure based on other serious skin reactions reported with celecoxib and valdecoxib exposure. Patients appear to be at highest risk for these reactions early in the course of therapy; the onset of the reaction occurring in the majority of cases within the first month of treatment.

Appropriate measures should be taken by physicians to monitor for any serious skin reactions with therapy, e.g. additional patient consultations. Patients should be advised to immediately report any emergent skin condition to their physician.

Parecoxib should be discontinued at the first appearance of skin rash, mucosal lesions, or any other sign of hypersensitivity. Serious skin reactions are known to occur with NSAIDs including COX-2 selective inhibitors as well as other medicinal products. However, the reported rate of serious skin events appears to be greater for valdecoxib (the active metabolite of parecoxib) as compared to other COX-2 selective inhibitors. Patients with a history of sulfonamide allergy may be at greater risk of skin reactions (see section 4.3). Patients without a history of sulfonamide allergy may also be at risk for serious skin reactions.

Hypersensitivity
Hypersensitivity reactions (anaphylaxis and angioedema) have been reported in post-marketing experience with valdecoxib and parecoxib (see section 4.8). Some of these reactions have occurred in patients with a history of allergic-type reactions to sulfonamides (see section 4.3). Parecoxib should be discontinued at the first sign of hypersensitivity.

Cases of severe hypotension shortly following parecoxib administration have been reported in postmarketing experience with parecoxib. Some of these cases have occurred without other signs of anaphylaxis. The physician should be prepared to treat severe hypotension.

Fluid retention, oedema, renal
As with other medicinal products known to inhibit prostaglandin synthesis, fluid retention and oedema have been observed in some patients taking parecoxib. Therefore, parecoxib should be used with caution in patients with compromised cardiac function, preexisting oedema, or other conditions predisposing to, or worsened by, fluid retention including those taking diuretic treatment or otherwise at risk of hypovolemia. If there is clinical evidence of deterioration in the condition of these patients, appropriate measures including discontinuation of parecoxib should be taken.

Acute renal failure has been reported through post-marketing surveillance in patients receiving parecoxib (see section 4.8). Since prostaglandin synthesis inhibition may result in deterioration of renal function and fluid retention, caution should be observed when administering Dynastat in patients with impaired renal function (see section 4.2) or hypertension, or in patients with compromised cardiac or hepatic function or other conditions predisposing to fluid retention.

Caution should be used when initiating treatment with Dynastat in patients with dehydration. In this case, it is advisable to rehydrate patients first and then start therapy with Dynastat.

Hypertension
As with all NSAIDs, parecoxib can lead to the onset of new hypertension or worsening of pre-existing hypertension, either of which may contribute to the increased incidence of cardiovascular events. Parecoxib should be used with caution in patients with hypertension. Blood pressure should be monitored closely during the initiation of therapy with parecoxib and throughout the course of therapy. If blood pressure rises significantly, alternative treatment should be considered.

Hepatic impairment
Dynastat should be used with caution in patients with moderate hepatic impairment (Child-Pugh score 7-9) (see section 4.2).

Use with oral anticoagulants
The concomitant use of NSAIDs with oral anticoagulants increases the risk of bleeding. Oral anticoagulants include warfarin/coumarin-type and novel oral anticoagulants (e.g. apixaban, dabigatran, and rivaroxaban) (see section 4.5).

4.5 Interaction with other medicinal products and other forms of interaction

Pharmacodynamic interactions
Anticoagulant therapy should be monitored, particularly during the first few days after initiating Dynastat therapy in patients receiving warfarin or other anticoagulants, since these patients have an increased risk of bleeding complications. Therefore, patients receiving oral anticoagulants should be closely monitored for their prothrombin time INR, particularly in the first few days when therapy with parecoxib is initiated or the dose of parecoxib is changed (see section 4.4).

Dynastat had no effect on acetylsalicylic acid-mediated inhibition of platelet aggregation or bleeding times. Clinical trials indicate that Dynastat can be given with low dose acetylsalicylic acid (≤ 325 mg). In the submitted studies, as with other NSAIDs, an increased risk of gastrointestinal ulceration or other gastrointestinal complications compared to use of parecoxib alone was shown for concomitant administration of low-dose acetylsalicylic acid (see section 5.1).

Coadministration of parecoxib and heparin did not affect the pharmacodynamics of heparin (activated partial thromboplastin time) compared to heparin alone.

Inhibition of prostaglandins by NSAIDs, including COX-2 inhibitors, may diminish the effect of angiotensin converting enzyme (ACE) inhibitors, angiotensin II antagonists, beta-blockers and diuretics. This interaction should be given consideration in patients receiving parecoxib concomitantly with ACE-inhibitors, angiotensin II antagonists, beta-blockers and diuretics.

In patients who are elderly, volume-depleted (including those on diuretic therapy), or with compromised renal function, coadministration of NSAIDs, including selective COX-2 inhibitors, with ACE inhibitors or Angiotensin-II antagonists, may result in further deterioration of renal function, including possible acute renal failure. These effects are usually reversible.

Therefore, the concomitant administration of these drugs should be done with caution. Patients should be adequately hydrated and the need to monitor the renal function should be assessed at the beginning of the concomitant treatment and periodically thereafter.

Coadministration of NSAIDs and ciclosporin or tacrolimus has been suggested to increase the nephrotoxic effect of ciclosporin and tacrolimus because of NSAID effects on renal prostaglandins. Renal function should be monitored when parecoxib and any of these medicinal products are coadministered.

Dynastat may be coadministered with opioid analgesics. In clinical trials, the daily requirement for PRN opioids was significantly reduced when coadministered with parecoxib.

Effects of other medicinal products on the pharmacokinetics of parecoxib (or its active metabolite valdecoxib)
Parecoxib is rapidly hydrolysed to the active metabolite valdecoxib. In humans, studies demonstrated that valdecoxib metabolism is predominantly mediated via CYP3A4 and 2C9 isozymes.

Plasma exposure (AUC and C_{max}) to valdecoxib was increased (62% and 19%, respectively) when coadministered with fluconazole (predominantly a CYP2C9 inhibitor), indicating that the dose of parecoxib should be reduced in those patients who are receiving fluconazole therapy.

Plasma exposure (AUC and C_{max}) to valdecoxib was increased (38% and 24%, respectively) when coadministered with ketoconazole (CYP3A4 inhibitor), however, a dosage adjustment should not generally be necessary for patients receiving ketoconazole.

The effect of enzyme induction has not been studied. The metabolism of valdecoxib may increase when coadministered with enzyme inducers such as rifampicin, phenytoin, carbamazepine or dexamethasone.

<u>Effect of parecoxib (or its active metabolite valdecoxib) on the pharmacokinetics of other medicinal products</u>
Treatment with valdecoxib (40 mg twice daily for 7 days) produced a 3-fold increase in plasma concentrations of dextromethorphan (CYP2D6 substrate). Therefore, caution should be observed when coadministering Dynastat and medicinal products that are predominantly metabolised by CYP2D6 and which have narrow therapeutic margins (e.g. flecainide, propafenone, metoprolol).

Plasma exposure of omeprazole (CYP 2C19 substrate) 40 mg once daily was increased by 46% following administration of valdecoxib 40 mg twice daily for 7 days, while the plasma exposure to valdecoxib was unaffected. These results indicate that although valdecoxib is not metabolised by CYP2C19, it may be an inhibitor of this isoenzyme. Therefore, caution should be observed when administering Dynastat with medicinal products known to be substrates of CYP2C19 (e.g. phenytoin, diazepam, or imipramine).

In two pharmacokinetic interaction studies in rheumatoid arthritis patients receiving a stable weekly methotrexate dose (5-20 mg/week, as a single oral or intramuscular dose), orally administered valdecoxib (10 mg twice daily or 40 mg twice daily) had little or no effect on the steady-state plasma concentrations of methotrexate. However caution is advised when methotrexate is administered concurrently with NSAIDs, because NSAID administration may result in increased plasma levels of methotrexate. Adequate monitoring of methotrexate-related toxicity should be considered when coadministering parecoxib and methotrexate.

Coadministration of valdecoxib and lithium produced significant decreases in lithium serum clearance (25%) and renal clearance (30%) with a 34% higher serum exposure compared to lithium alone. Lithium serum concentration should be monitored closely when initiating or changing parecoxib therapy in patients receiving lithium.

Coadministration of valdecoxib with glibenclamide (CYP3A4 substrate) did not affect either the pharmacokinetics (exposure) or the pharmacodynamics (blood glucose and insulin levels) of glibenclamide.

<u>Injectable anaesthetics</u>
Coadministration of IV parecoxib 40 mg with propofol (CYP2C9 substrate) or midazolam (CYP3A4 substrate) did not affect either the pharmacokinetics (metabolism and exposure) or the pharmacodynamics (EEG effects, psychomotor tests and waking from sedation) of IV propofol or IV midazolam. Additionally, coadministration of valdecoxib had no clinically significant effect on the hepatic or intestinal CYP 3A4-mediated metabolism of orally administered midazolam. Administration of IV parecoxib 40 mg had no significant effect on the pharmacokinetics of either IV fentanyl or IV alfentanil (CYP3A4 substrates).

<u>Inhalation anaesthetics</u>
No formal interaction studies have been done. In surgery studies in which parecoxib was administered pre-operatively, no evidence of pharmacodynamic interaction was observed in patients receiving parecoxib and the inhalation anaesthetic agents nitrous oxide and isoflurane (see section 5.1).

4.6 Fertility, pregnancy and lactation

Pregnancy
Parecoxib is suspected to cause serious birth defects when administered during the last trimester of pregnancy because as with other medicinal products known to inhibit prostaglandin, it may cause premature closure of the ductus arteriosus or uterine inertia (see sections 4.3, 5.1 and 5.3).

NSAID use during the second or third trimester of pregnancy may cause foetal renal dysfunction which may result in reduction of amniotic fluid volume or oligohydramnios in severe cases. Such effects may occur shortly after treatment initiation and are usually reversible. Pregnant women on NSAIDs should be closely monitored for amniotic fluid volume.

Dynastat is contraindicated in the third trimester of pregnancy (see section 4.3).

There are no adequate data from the use of parecoxib in pregnant women or during labour. However, inhibition of prostaglandin synthesis might adversely affect pregnancy. Data from epidemiological studies suggest an increased risk of miscarriage after use of prostaglandin synthesis inhibitors in early pregnancy. In animals, administration of prostaglandin synthesis inhibitors, including parecoxib, has been shown to result in increased pre- and post-implantation loss and embryo-foetal lethality (see sections 5.1 and 5.3). During the first and second trimester of pregnancy, Dynastat should not be given unless clearly necessary.

Breast-feeding
Administration of a single dose of parecoxib to lactating women following caesarean section resulted in the transfer of a relatively small amount of parecoxib and its active metabolite valdecoxib into human milk, and this resulted in a low relative dose for the infant (approximately 1% of the weight-adjusted maternal dose). Dynastat must not be administered to women who breast-feed (see section 4.3).

Fertility
The use of Dynastat, as with any medicinal product known to inhibit cyclooxygenase/prostaglandin synthesis, is not recommended in women attempting to conceive (see sections 4.3, 5.1 and 5.3).

Based on the mechanism of action, the use of NSAIDs, may delay or prevent rupture of ovarian follicles, which has been associated with reversible infertility in some women. In women who have difficulties conceiving or who are undergoing investigation of infertility, withdrawal of NSAIDs, including Dynastat should be considered.

4.7 Effects on ability to drive and use machines

Patients who experience dizziness, vertigo or somnolence after receiving Dynastat should refrain from driving or operating machines.

4.8 Undesirable effects

Summary of the safety profile
The most common adverse reaction for Dynastat is nausea. The most serious reactions occur uncommonly to rarely, and include cardiovascular events such as myocardial infarction and severe hypotension, as well as hypersensitivity events such as anaphylaxis, angioedema and severe skin reactions. Following coronary artery bypass graft surgery, patients administered Dynastat have a higher risk of adverse reactions such as: cardiovascular/thromboembolic events (including myocardial infarction, stroke/TIA, pulmonary embolus, and deep vein thrombosis; see sections 4.3 and 5.1), deep surgical infections, and sternal wound healing complications.

Tabulated list of adverse reactions
The following adverse reactions were reported for patients who received parecoxib (N=5,402) in 28 placebo-controlled clinical trials. Reports from post-marketing experience have been listed as "frequency not known" because the respective frequencies cannot be estimated from the available data. Within each frequency grouping, adverse reactions are listed using MedDRA terminology and presented in order of decreasing seriousness.

**Adverse Drug Reaction Frequency**				
Very Common (≥1/10)	*Common* (≥1/100 to <1/10)	*Uncommon* (≥1/1000 to <1/100)	*Rare* (≥1/10,000 to <1/1000)	*Not known*
Infections and infestations				
	Pharyngitis, alveolar osteitis (dry socket)	Abnormal sternal serous wound drainage, wound infection		
Blood and lymphatic system disorders				
	Anaemia postoperative	Thrombocytopenia		
Immune system disorders				
			Anaphylactoid reaction	
Metabolism and nutrition disorders				
	Hypokalaemia	Hyperglycaemia, anorexia		
Psychiatric disorders				
	Agitation, insomnia			
Nervous system disorders				
	Hypoaesthesia, dizziness	Cerebrovascular disorder		
Ear and labyrinth disorders				
		Ear pain		
Cardiac disorders				
		Myocardial infarction, bradycardia		Circulatory collapse, congestive heart failure, tachycardia

**Adverse Drug Reaction Frequency**				
Very Common (≥1/10)	*Common (≥1/100 to <1/10)*	*Uncommon (≥1/1000 to <1/100)*	*Rare (≥1/10,000 to <1/1000)*	*Not known*
Vascular disorders				
	Hypertension, hypotension	Hypertension (aggravated), orthostatic hypotension		
Respiratory, thoracic and mediastinal disorders				
	Respiratory insufficiency	Pulmonary embolism		Dyspnoea
Gastrointestinal disorders				
Nausea	Abdominal pain, vomiting, constipation, dyspepsia, flatulence	Gastroduodenal ulceration, gastrooesophageal reflux disease, dry mouth, gastrointestinal sounds abnormal	Pancreatitis, oesophagitis, oedema mouth (perioral swelling)	
Skin and subcutaneous tissue disorders				
	Pruritus, hyperhidrosis	Ecchymosis, rash, urticaria		Stevens-Johnson syndrome, erythema multiforme, exfoliative dermatitis
Musculoskeletal and connective tissue disorders				
	Back pain	Arthralgia		
Renal and urinary disorders				
	Oliguria		Renal failure acute	Renal failure,
General disorders and administration site conditions				
	Oedema peripheral	Asthenia, injection site pain, injection site reaction		Hypersensitivity reactions including anaphylaxis and angioedema
Investigations				
	Blood creatinine increased	Blood CPK increased, blood LDH increased, SGOT increased, SGPT increased, BUN increased.		
Injury, poisoning and procedural complications				
		Post procedural complication (skin)		

Description of selected adverse reactions

In post-marketing experience, toxic epidermal necrolysis has been reported in association with the use of valdecoxib, and cannot be ruled out for parecoxib (see section 4.4). In addition, the

following rare, serious adverse reactions have been reported in association with the use of NSAIDs and cannot be ruled out for Dynastat: bronchospasm and hepatitis.

Reporting of suspected adverse reactions
Reporting suspected adverse reactions after authorisation of the medicinal product is important. It allows continued monitoring of the benefit/risk balance of the medicinal product. Healthcare professionals are asked to report any suspected adverse reactions via the national reporting system listed in Appendix V

4.9 Overdose

Reporting of overdose with parecoxib has been associated with adverse reactions which have also been described with recommended doses of parecoxib.

In case of overdose, patients should be managed by symptomatic and supportive care. Valdecoxib is not removed by haemodialysis. Diuresis or alkalisation of urine may not be useful due to high protein binding of valdecoxib.

5. PHARMACOLOGICAL PROPERTIES

5.1 Pharmacodynamic properties

Pharmacotherapeutic group: Antiinflammatory and antirheumatic products, Coxibs, ATC code: M01AH04

Parecoxib is a prodrug of valdecoxib. Valdecoxib is a selective COX-2 inhibitor within the clinical dose range. Cyclooxygenase is responsible for generation of prostaglandins. Two isoforms, COX-1 and COX-2, have been identified. COX-2 is the isoform of the enzyme that has been shown to be induced by pro-inflammatory stimuli and has been postulated to be primarily responsible for the synthesis of prostanoid mediators of pain, inflammation, and fever. COX-2 is also involved in ovulation, implantation and closure of the ductus arteriosus, regulation of renal function, and central nervous system functions (fever induction, pain perception and cognitive function). It may also play a role in ulcer healing. COX-2 has been identified in tissue around gastric ulcers in man but its relevance to ulcer healing has not been established.

The difference in antiplatelet activity between some COX-1 inhibiting NSAIDs and COX-2 selective inhibitors may be of clinical significance in patients at risk of thrombo-embolic reactions. COX-2 selective inhibitors reduce the formation of systemic (and therefore possibly endothelial) prostacyclin without affecting platelet thromboxane. The clinical relevance of these observations has not been established.

Parecoxib has been used in a range of major and minor surgeries. The efficacy of Dynastat was established in studies of dental, gynaecologic (hysterectomy), orthopaedic (knee and hip replacement), and coronary artery bypass graft surgical pain. The first perceptible analgesic effect occurred in 7-13 minutes, with clinically meaningful analgesia demonstrated in 23-39 minutes and a peak effect within 2 hours following administration of single doses of 40 mg IV or IM Dynastat. The magnitude of analgesic effect of the 40 mg dose was comparable with that of ketorolac 60 mg IM or ketorolac 30 mg IV. After a single dose, the duration of analgesia was dose and clinical pain model dependent, and ranged from 6 to greater than 12 hours.

Use of parecoxib beyond 3 days
Most trials were designed for dosing of parecoxib up to 3 days. Data from 3 randomised placebo-controlled trials, where the protocols allowed treatment of parecoxib for >3 days was

pooled and analysed. In the pooled analysis of 676 patients, 318 received placebo and 358 received parecoxib. Of the patients treated with parecoxib, 317 patients received parecoxib for up to 4 days, 32 patients for up to 5 days, while only 8 patients were treated for up to 6 days and 1 patient for 7 or more days. Of the patients treated with placebo, 270 patients received placebo for up to 4 days, 43 patients for up to 5 days, while only 3 patients were treated for up to 6 days and 2 patients for 7 or more days. Both groups had similar demographics. The mean (SD) duration of treatment was 4.1 (0.4) days for parecoxib and 4.2 (0.5) days for placebo, the range was 4-7 days for parecoxib and 4-9 days for placebo. The occurrence of adverse events in patients receiving parecoxib for 4-7 days (median duration 4 days) was low after treatment Day 3 and similar to placebo.

<u>Opioid-sparing effects</u>
In a placebo-controlled, orthopedic and general surgery study (n =1050), patients received Dynastat at an initial parenteral dose of 40 mg IV followed by 20 mg twice daily for a minimum of 72 hours in addition to receiving standard care including supplemental patient controlled opioids. The reduction in opioid use with Dynastat treatment on Days 2 and 3 was 7.2 mg and 2.8 mg (37% and 28% respectively).This reduction in opioid use was accompanied by significant reductions in patient-reported opioid symptom distress. Added pain relief compared to opioids alone was shown. Additional studies in other surgical settings provided similar observations. There are no data indicating less overall adverse events associated with the use of parecoxib compared to placebo when used in conjunction with opioids.

<u>Gastrointestinal studies</u>
In short-term studies (7 days), the incidence of endoscopically observed gastroduodenal ulcers or erosions in healthy young and elderly (≥ 65 years) subjects administered Dynastat (5-21%), although higher than placebo (5-12%), was statistically significantly lower than the incidence observed with NSAIDs (66-90%).

<u>CABG post-operative safety studies</u>
In addition to routine adverse event reporting, pre-specified event categories, adjudicated by an independent expert committee, were examined in two placebo-controlled safety studies in which patients received parecoxib for at least 3 days and then were transitioned to oral valdecoxib for a total duration of 10-14 days. All patients received standard of care analgesia during treatment. Patients received low-dose acetylsalicylic acid prior to randomization and throughout the two CABG surgery studies.

The first CABG surgery study evaluated patients treated with IV parecoxib 40 mg bid for a minimum of 3 days, followed by treatment with valdecoxib 40 mg bid (parecoxib/valdecoxib group) (n=311) or placebo/placebo (n=151) in a 14-day, double-blind placebo-controlled study. Nine pre-specified adverse event categories were evaluated (cardiovascular thromboembolic events, pericarditis, new onset or exacerbation of congestive heart failure, renal failure/dysfunction, upper GI ulcer complications, major non-GI bleeds, infections, non-infectious pulmonary complications, and death). There was a significantly ($p<0.05$) greater incidence of cardiovascular/thromboembolic events (myocardial infarction, ischemia, cerebrovascular accident, deep vein thrombosis and pulmonary embolism) detected in the parecoxib/valdecoxib treatment group compared to the placebo/placebo treatment group for the IV dosing period (2.2% and 0.0% respectively) and over the entire study period (4.8% and 1.3% respectively). Surgical wound complications (most involving the sternal wound) were observed at an increased rate with parecoxib/valdecoxib treatment.

In the second CABG surgery study, four pre-specified event categories were evaluated (cardiovascular/thromboembolic; renal dysfunction/renal failure; upper GI ulcer/bleeding; surgical wound complication). Patients were randomized within 24-hours post-CABG surgery to: parecoxib initial dose of 40 mg IV, then 20 mg IV Q12H for a minimum of 3 days followed by valdecoxib PO (20 mg Q12H) (n=544) for the remainder of a 10 day treatment period; placebo IV followed by valdecoxib PO (n=544); or placebo IV followed by placebo PO (n=548). A significantly ($p=0.033$)

greater incidence of events in the cardiovascular/thromboembolic category was detected in the parecoxib/valdecoxib treatment group (2.0%) compared to the placebo/placebo treatment group (0.5%). Placebo/valdecoxib treatment was also associated with a higher incidence of CV thromboembolic events versus placebo treatment, but this difference did not reach statistical significance. Three of the six cardiovascular thromboembolic events in the placebo/valdecoxib treatment group occurred during the placebo treatment period; these patients did not receive valdecoxib. Pre-specified events that occurred with the highest incidence in all three treatment groups involved the category of surgical wound complications, including deep surgical infections and sternal wound healing events.

There were no significant differences between active treatments and placebo for any of the other pre-specified event categories (renal dysfunction/failure, upper GI ulcer complications or surgical wound complications).

General surgery
In a large (N=1050) major orthopedic/general surgery trial, patients received an initial dose of parecoxib 40 mg IV, then 20 mg IV Q12H for a minimum of 3 days followed by valdecoxib PO (20 mg Q12H) (n=525) for the remainder of a 10 day treatment period, or placebo IV followed by placebo PO (n=525). There were no significant differences in the overall safety profile, including the four pre-specified event categories described above for the second CABG surgery study, for parecoxib/valdecoxib compared to placebo treatment in these post-surgical patients.

Platelet studies
In a series of small, multiple dose studies in healthy young and elderly subjects, Dynastat 20 mg or 40 mg twice daily had no effect on platelet aggregation or bleeding compared to placebo. In young subjects, Dynastat 40 mg twice daily had no clinically significant effect on acetylsalicylic acid-mediated inhibition of platelet function (see section 4.5).

5.2 Pharmacokinetic properties

Following IV or IM injection, parecoxib is rapidly converted to valdecoxib, the pharmacologically active substance, by enzymatic hydrolysis in the liver.

Absorption
Exposure of valdecoxib following single doses of Dynastat, as measured by both the area under the plasma concentration vs. time curve (AUC) and peak concentration (C_{max}), is approximately linear in the range of clinical doses. AUC and C_{max} following twice daily administration is linear up to 50 mg IV and 20 mg IM. Steady state plasma concentrations of valdecoxib were reached within 4 days with twice daily dosing.

Following single IV and IM doses of parecoxib 20 mg, C_{max} of valdecoxib is achieved in approximately 30 minutes and approximately 1 hour, respectively. Exposure to valdecoxib was similar in terms of AUC and C_{max} following IV and IM administration. Exposure to parecoxib was similar after IV or IM administration in terms of AUC. Average C_{max} of parecoxib after IM dosing was lower compared to bolus IV dosing, which is attributed to slower extravascular absorption after IM administration. These decreases were not considered clinically important since C_{max} of valdecoxib is comparable after IM and IV parecoxib administration.

Distribution
The volume of distribution of valdecoxib after its IV administration is approximately 55 litres. Plasma protein binding is approximately 98% over the concentration range achieved with the highest recommended dose, 80 mg/day. Valdecoxib, but not parecoxib, is extensively partitioned into erythrocytes.

Biotransformation
Parecoxib is rapidly and almost completely converted to valdecoxib and propionic acid in vivo with a plasma half-life of approximately 22 minutes. Elimination of valdecoxib is by extensive hepatic metabolism involving multiple pathways, including cytochrome P 450 (CYP) 3A4 and CYP2C9 isoenzymes and glucuronidation (about 20%) of the sulfonamide moiety. A hydroxylated metabolite of valdecoxib (via the CYP pathway) has been identified in human plasma that is active as a COX-2 inhibitor. It represents approximately 10% of the concentration of valdecoxib; because of this metabolite's low concentration, it is not expected to contribute a significant clinical effect after administration of therapeutic doses of parecoxib.

Elimination
Valdecoxib is eliminated via hepatic metabolism with less than 5% unchanged valdecoxib recovered in the urine. No unchanged parecoxib is detected in urine and only trace amounts in the faeces. About 70% of the dose is excreted in the urine as inactive metabolites. Plasma clearance (CL_p) for valdecoxib is about 6 l/hr. After IV or IM dosing of parecoxib, the elimination half-life ($t_{1/2}$) of valdecoxib is about 8 hours.

Elderly
Dynastat has been administered to 335 elderly patients (65-96 years of age) in pharmacokinetic and therapeutic trials. In healthy elderly subjects, the apparent oral clearance of valdecoxib was reduced, resulting in an approximately 40% higher plasma exposure of valdecoxib compared to healthy young subjects. When adjusted for body weight, steady state plasma exposure of valdecoxib was 16% higher in elderly females compared to elderly males (see section 4.2).

Renal impairment
In patients with varying degrees of renal impairment administered 20 mg IV Dynastat, parecoxib was rapidly cleared from plasma. Because renal elimination of valdecoxib is not important to its disposition, no changes in valdecoxib clearance were found even in patients with severe renal impairment or in patients undergoing dialysis (see section 4.2).

Hepatic impairment
Moderate hepatic impairment did not result in a reduced rate or extent of parecoxib conversion to valdecoxib. In patients with moderate hepatic impairment (Child-Pugh score 7-9), treatment should be initiated with half the usual recommended dose of Dynastat and the maximum daily dose should be reduced to 40 mg since valdecoxib exposures were more than doubled (130%) in these patients. Patients with severe hepatic impairment have not been studied and therefore the use of Dynastat in patients with severe hepatic impairment is not recommended (see sections 4.2 and 4.3).

5.3 Preclinical safety data

Non-clinical data reveal no special hazard for humans based on conventional studies of safety pharmacology or repeated dose toxicity at 2-fold the maximum human exposure to parecoxib. However, in the repeated dose toxicity studies in dogs and rats, the systemic exposures to valdecoxib (the active metabolite of parecoxib) were approximately 0.8-fold the systemic exposure in elderly human subjects at the maximum recommended therapeutic dose of 80 mg daily. Higher doses were associated with aggravation and delayed healing of skin infections, an effect probably associated with COX-2 inhibition.

In reproduction toxicity tests, the incidence of post-implantation losses, resorptions and foetal body weight retardation occurred at doses not producing maternal toxicity in the rabbit studies. No effects of parecoxib on male or female fertilities were found in rats.

The effects of parecoxib have not been evaluated in late pregnancy or in the pre- and postnatal period.

Parecoxib administered intravenously to lactating rats as a single dose showed concentrations of parecoxib, valdecoxib and a valdecoxib active metabolite in milk similar to that of maternal plasma.

The carcinogenic potential of parecoxib has not been evaluated.

6. PHARMACEUTICAL PARTICULARS

6.1 List of excipients

Powder
Disodium hydrogen phosphate
Phosphoric acid and/or sodium hydroxide (for pH adjustment).

Solvent
Sodium chloride
Hydrochloric acid or sodium hydroxide (for pH adjustment)
Water for injection.

6.2 Incompatibilities

This medicinal product must not be mixed with other medicinal products except for those mentioned in section 6.6.

Dynastat and opioids should not be administered together in the same syringe.

Use of Ringer-Lactate solution for injection or glucose 50 mg/ml (5%) in Ringer Lactate solution for injection for reconstitution will cause the parecoxib to precipitate from solution and therefore is **not** recommended.

Use of water for injection is **not** recommended, as the resulting solution is not isotonic.

Dynastat should not be injected into an IV line delivering any other medicinal product. The IV line must be adequately flushed prior to and after Dynastat injection with a solution of known compatibility (see section 6.6).

Injection into an IV line delivering glucose 50 mg/ml (5%) in Ringer-Lactate solution for injection, or other IV fluids not listed in section 6.6, is not recommended as this may cause precipitation from solution.

6.3 Shelf life

The shelf life of the unreconstituted product is 3 years.

Chemical and physical in-use stability of the reconstituted solution, which should not be refrigerated or frozen, have been demonstrated for up to 24 hours at 25°C. Thus, 24 hours should be considered the maximum shelf life of the reconstituted product. However, due to the importance of microbiological infection risk for injectable products, the reconstituted solution should be used immediately unless reconstitution has taken place in controlled and validated aseptic conditions. Unless such requirements are met, in-storage times and conditions prior to use are the responsibility of the user, and would not normally be longer than 12 hours at 25°C.

6.4 Special precautions for storage

This medicinal product does not require any special storage conditions prior to reconstitution.

For storage conditions of the reconstituted medicinal product see section 6.3.

6.5 Nature and contents of container

Parecoxib sodium vials
Type I colourless glass vials (5 ml) with a butyl rubber stopper, sealed with a purple polypropylene flip-off cap on the aluminium overseal.

Solvent ampoules
2 ml ampoule: colourless neutral glass, Type I.

Dynastat is supplied as a sterile, single unit-of-use vial that is packaged with a 2 ml ampoule with a fill volume of 2 ml sodium chloride 9 mg/ml (0.9%) solution (see below for various pack sizes and configurations)

Pack sizes
1 + 1 pack: contains 1 powder vial and 1 solvent ampoule.
3 + 3 pack: contains 3 powder vials and 3 solvent ampoules.
5 + 5 pack: contains 5 powder vials and 5 solvent ampoules.

Not all pack sizes may be marketed.

6.6 Special precautions for disposal and other handling

Dynastat must be reconstituted before use. Dynastat is preservative free. Aseptic technique is required for its preparation.

Reconstitution solvents
Reconstitute Dynastat 40 mg with 2 ml sodium chloride 9 mg/ml (0.9%) solution.

The **only** other acceptable solvents for reconstitution are:
- glucose 50 mg/ml (5%) solution for infusion
- sodium chloride 4.5 mg/ml (0.45%) and glucose 50 mg/ml (5%) solution for injection/infusion

Reconstitution process
Use aseptic technique to reconstitute lyophilised parecoxib (as parecoxib).
Remove the purple flip-off cap to expose the central portion of the rubber stopper of the 40 mg parecoxib vial. Withdraw, with a sterile needle and syringe, 2 ml of an acceptable solvent and insert the needle through the central portion of the rubber stopper transferring the solvent into the 40 mg vial. Dissolve the powder completely using a gentle swirling motion and inspect the reconstituted product before use. The entire contents of the vial should be withdrawn for a single administration.

After reconstitution, the liquid should be a clear solution. Dynastat should be inspected visually for particulate matter and discoloration prior to administration. The solution should not be used if discolored or cloudy, or if particulate matter is observed. Dynastat should be administered within 24 hours of reconstitution (see section 6.3), or discarded.

The reconstituted product is isotonic.

IV line solution compatibility
After reconstitution with acceptable solvents, Dynastat may **only** be injected IV or IM, or into IV lines delivering:

- sodium chloride 9 mg/ml (0.9%) solution for injection/infusion;
- glucose 50 mg/ml (5%) solution for infusion;
- sodium chloride 4.5 mg/ml (0.45%) and glucose 50 mg/ml (5%) solution for injection/infusion;
  or
- Ringer-Lactate solution for injection.

For single use only. Any unused medicinal product or waste material should be disposed of in accordance with local requirements.

7. MARKETING AUTHORISATION HOLDER

Pfizer Europe MA EEIG
Boulevard de la Plaine 17
1050 Bruxelles
Belgium

8. MARKETING AUTHORISATION NUMBER(S)

EU/1/02/209/006-008

9. DATE OF FIRST AUTHORISATION/RENEWAL OF THE AUTHORISATION

Date of first authorisation: 22 March 2002
Date of latest renewal: 24 January 2012

10. DATE OF REVISION OF THE TEXT

Detailed information on this medicinal product is available on the website of the European Medicines Agency http://www.ema.europa.eu.

ANNEX II

A. MANUFACTURER RESPONSIBLE FOR BATCH RELEASE

B. CONDITIONS OR RESTRICTIONS REGARDING SUPPLY

C. OTHER CONDITIONS AND REQUIREMENTS OF THE MARKETING AUTHORISATION

D. CONDITIONS OR RESTRICTIONS WITH REGARD TO THE SAFE AND EFFECTIVE USE OF THE MEDICINAL PRODUCT

A. MANUFACTURER RESPONSIBLE FOR BATCH RELEASE

Name and address of the manufacturer responsible for batch release

Pfizer Manufacturing Belgium NV
Rijksweg 12
2870 Puurs
Belgium

B. CONDITIONS OR RESTRICTIONS REGARDING SUPPLY AND USE

Medicinal product subject to medical prescription.

C. OTHER CONDITIONS AND REQUIREMENTS OF THE MARKETING AUTHORISATION

- **Periodic safety update reports (PSURs)**

The requirements for submission of PSURs for this medicinal product are set out in the list of Union reference dates (EURD list) provided for under Article 107c(7) of Directive 2001/83/EC and any subsequent updates published on the European medicines web-portal.

D. CONDITIONS OR RESTRICTIONS WITH REGARD TO THE SAFE AND EFFECTIVE USE OF THE MEDICINAL PRODUCT

- **Risk management plan (RMP)**

The marketing authorisation holder (MAH) shall perform the required pharmacovigilance activities and interventions detailed in the agreed RMP presented in Module 1.8.2 of the marketing authorisation and any agreed subsequent updates of the RMP.

An updated RMP should be submitted:

- At the request of the European Medicines Agency;
- Whenever the risk management system is modified, especially as the result of new information being received that may lead to a significant change to the benefit/risk profile or as the result of an important (pharmacovigilance or risk minimisation) milestone being reached.

ANNEX III

LABELLING AND PACKAGE LEAFLET

A. LABELLING

PARTICULARS TO APPEAR ON THE OUTER PACKAGING AND THE IMMEDIATE PACKAGING

OUTER CARTON: 40 mg vials

CARTON TEXT - EU/1/02/209/005

1. NAME OF THE MEDICINAL PRODUCT

Dynastat 40 mg powder for solution for injection
parecoxib

2. STATEMENT OF ACTIVE SUBSTANCE(S)

Each vial contains 40 mg parecoxib, as 42.36 mg parecoxib sodium. After reconstitution with 2 ml of solvent, the concentration of parecoxib is 20 mg/ml. Thus, each 2 ml of reconstituted solution contains 40 mg of parecoxib.

3. LIST OF EXCIPIENTS

Also contains disodium hydrogen phosphate, phosphoric acid and sodium hydroxide.

4. PHARMACEUTICAL FORM AND CONTENTS

Powder for solution for injection

10 vials

5. METHOD AND ROUTE(S) OF ADMINISTRATION

Read the package leaflet before use.
For single use only.
Intravenous or intramuscular use.

6. SPECIAL WARNING THAT THE MEDICINAL PRODUCT MUST BE STORED OUT OF THE SIGHT AND REACH OF CHILDREN

Keep out of the sight and reach of children.

7. OTHER SPECIAL WARNING(S), IF NECESSARY

8. EXPIRY DATE

EXP
The reconstituted product should be used immediately (up to 24 hours if prepared aseptically), and should not be frozen or refrigerated.

9. SPECIAL STORAGE CONDITIONS

This medicinal product does not require any special storage conditions prior to reconstitution. For more information on storage see the package leaflet.

10. SPECIAL PRECAUTIONS FOR DISPOSAL OF UNUSED MEDICINAL PRODUCTS OR WASTE MATERIALS DERIVED FROM SUCH MEDICINAL PRODUCTS, IF APPROPRIATE

11. NAME AND ADDRESS OF THE MARKETING AUTHORISATION HOLDER

Pfizer Europe MA EEIG
Boulevard de la Plaine 17
1050 Bruxelles
Belgium

12. MARKETING AUTHORISATION NUMBER(S)

EU/1/02/209/005

13. BATCH NUMBER

Lot

14. GENERAL CLASSIFICATION FOR SUPPLY

15. INSTRUCTIONS ON USE

16. INFORMATION IN BRAILLE

Dynastat 40 mg

17. UNIQUE IDENTIFIER – 2D BARCODE

2D barcode carrying the unique identifier included.

18. UNIQUE IDENTIFIER – HUMAN READABLE DATA

PC
SN
NN

MINIMUM PARTICULARS TO APPEAR ON SMALL IMMEDIATE PACKAGING UNITS

VIAL LABEL: 40 mg

TEXT FOR VIAL LABEL - EU/1/02/209/005

1. NAME OF THE MEDICINAL PRODUCT AND ROUTE(S) OF ADMINISTRATION

Dynastat 40 mg powder for injection
parecoxib
IV/IM

2. METHOD OF ADMINISTRATION

Read the package leaflet before use.

3. EXPIRY DATE

EXP

4. BATCH NUMBER

Lot

5. CONTENTS BY WEIGHT, BY VOLUME OR BY UNIT

6. OTHER

PARTICULARS TO APPEAR ON THE OUTER PACKAGING AND THE IMMEDIATE PACKAGING

OUTER CARTON: 40 mg powder and solvent for solution for injection

CARTON TEXT - EU/1/02/209/006

1. NAME OF THE MEDICINAL PRODUCT

Dynastat 40 mg powder and solvent for solution for injection
parecoxib

2. STATEMENT OF ACTIVE SUBSTANCE(S)

Each vial contains 40 mg parecoxib, as 42.36 mg parecoxib sodium. After reconstitution with 2 ml of solvent, the concentration of parecoxib is 20 mg/ml. Thus, each 2 ml of reconstituted solution provides 40 mg of parecoxib.

3. LIST OF EXCIPIENTS

Also contains disodium hydrogen phosphate, phosphoric acid and sodium hydroxide.

2 ml solvent ampoule contains sodium chloride, hydrochloric acid, sodium hydroxide and water for injection.

4. PHARMACEUTICAL FORM AND CONTENTS

Powder and solvent for solution for injection

1 vial and 1 solvent ampoule

5. METHOD AND ROUTE(S) OF ADMINISTRATION

Read the package leaflet before use.
For single use only.
Intravenous or intramuscular use.

6. SPECIAL WARNING THAT THE MEDICINAL PRODUCT MUST BE STORED OUT OF THE SIGHT AND REACH OF CHILDREN

Keep out of the sight and reach of children.

7. OTHER SPECIAL WARNING(S), IF NECESSARY

8. EXPIRY DATE

EXP
The reconstituted product should be used immediately (up to 24 hours if prepared aseptically), and should not be frozen or refrigerated.

9. SPECIAL STORAGE CONDITIONS

This medicinal product does not require any special storage conditions prior to reconstitution. For more information on storage see the package leaflet.

10. SPECIAL PRECAUTIONS FOR DISPOSAL OF UNUSED MEDICINAL PRODUCTS OR WASTE MATERIALS DERIVED FROM SUCH MEDICINAL PRODUCTS, IF APPROPRIATE

11. NAME AND ADDRESS OF THE MARKETING AUTHORISATION HOLDER

Pfizer Europe MA EEIG
Boulevard de la Plaine 17
1050 Bruxelles
Belgium

12. MARKETING AUTHORISATION NUMBER(S)

EU/1/02/209/006

13. BATCH NUMBER

Lot

14. GENERAL CLASSIFICATION FOR SUPPLY

15. INSTRUCTIONS ON USE

16. INFORMATION IN BRAILLE

Dynastat 40 mg

17. UNIQUE IDENTIFIER – 2D BARCODE

2D barcode carrying the unique identifier included.

18. UNIQUE IDENTIFIER – HUMAN READABLE DATA

PC
SN
NN

PARTICULARS TO APPEAR ON THE OUTER PACKAGING AND THE IMMEDIATE PACKAGING

OUTER CARTON TEXT: 40 mg powder and solvent for solution for injection

CARTON TEXT - EU/1/02/209/007

1. NAME OF THE MEDICINAL PRODUCT

Dynastat 40 mg powder and solvent for solution for injection
parecoxib

2. STATEMENT OF ACTIVE SUBSTANCE(S)

Each vial contains 40 mg parecoxib, as 42.36 mg parecoxib sodium. After reconstitution with 2 ml of solvent, the concentration of parecoxib is 20 mg/ml. Thus, each 2 ml of reconstituted solution provides 40 mg of parecoxib

3. LIST OF EXCIPIENTS

Also contains disodium hydrogen phosphate, phosphoric acid and sodium hydroxide.

2 ml solvent ampoule contains sodium chloride, hydrochloric acid, sodium hydroxide and water for injection.

4. PHARMACEUTICAL FORM AND CONTENTS

Powder and solvent for solution for injection

3 vials and 3 solvent ampoules

5. METHOD AND ROUTE(S) OF ADMINISTRATION

Read the package leaflet before use.
For single use only.
Intravenous or intramuscular use.

6. SPECIAL WARNING THAT THE MEDICINAL PRODUCT MUST BE STORED OUT OF THE SIGHT AND REACH OF CHILDREN

Keep out of the sight and reach of children.

7. OTHER SPECIAL WARNING(S), IF NECESSARY

8. EXPIRY DATE

EXP
The reconstituted product should be used immediately (up to 24 hours if prepared aseptically), and should not be frozen or refrigerated.

9. SPECIAL STORAGE CONDITIONS

This medicinal product does not require any special storage conditions prior to reconstitution. For more information on storage see the package leaflet.

10. SPECIAL PRECAUTIONS FOR DISPOSAL OF UNUSED MEDICINAL PRODUCTS OR WASTE MATERIALS DERIVED FROM SUCH MEDICINAL PRODUCTS, IF APPROPRIATE

11. NAME AND ADDRESS OF THE MARKETING AUTHORISATION HOLDER

Pfizer Europe MA EEIG
Boulevard de la Plaine 17
1050 Bruxelles
Belgium

12. MARKETING AUTHORISATION NUMBER(S)

EU/1/02/209/007

13. BATCH NUMBER

Lot

14. GENERAL CLASSIFICATION FOR SUPPLY

15. INSTRUCTIONS ON USE

16. INFORMATION IN BRAILLE

Dynastat 40 mg

17. UNIQUE IDENTIFIER – 2D BARCODE

2D barcode carrying the unique identifier included.

18. UNIQUE IDENTIFIER – HUMAN READABLE DATA

PC
SN
NN

PARTICULARS TO APPEAR ON THE OUTER PACKAGING AND THE IMMEDIATE PACKAGING

OUTER CARTON TEXT: 40 mg powder and solvent for solution for injection

CARTON TEXT - EU/1/02/209/008

1. NAME OF THE MEDICINAL PRODUCT

Dynastat 40 mg powder and solvent for solution for injection
parecoxib

2. STATEMENT OF ACTIVE SUBSTANCE(S)

Each vial contains 40 mg parecoxib, as 42.36 mg parecoxib sodium. After reconstitution with 2 ml of solvent, the concentration of parecoxib is 20 mg/ml. Thus, each 2 ml of reconstituted solution provides 40 mg of parecoxib

3. LIST OF EXCIPIENTS

Also contains disodium hydrogen phosphate, phosphoric acid and sodium hydroxide.

2 ml solvent ampoule contains sodium chloride, hydrochloric acid, sodium hydroxide and water for injection.

4. PHARMACEUTICAL FORM AND CONTENTS

Powder and solvent for solution for injection

5 vials and 5 solvent ampoules

5. METHOD AND ROUTE(S) OF ADMINISTRATION

Read the package leaflet before use.
For single use only.
Intravenous or intramuscular use

6. SPECIAL WARNING THAT THE MEDICINAL PRODUCT MUST BE STORED OUT OF THE SIGHT AND REACH OF CHILDREN

Keep out of the sight and reach of children.

7. OTHER SPECIAL WARNING(S), IF NECESSARY

8. EXPIRY DATE

EXP
The reconstituted product should be used immediately (up to 24 hours if prepared aseptically), and should not be frozen or refrigerated.

9. SPECIAL STORAGE CONDITIONS

This medicinal product does not require any special storage conditions prior to reconstitution. For more information on storage see the package leaflet.

10. SPECIAL PRECAUTIONS FOR DISPOSAL OF UNUSED MEDICINAL PRODUCTS OR WASTE MATERIALS DERIVED FROM SUCH MEDICINAL PRODUCTS, IF APPROPRIATE

11. NAME AND ADDRESS OF THE MARKETING AUTHORISATION HOLDER

Pfizer Europe MA EEIG
Boulevard de la Plaine 17
1050 Bruxelles
Belgium

12. MARKETING AUTHORISATION NUMBER(S)

EU/1/02/209/008

13. BATCH NUMBER

Lot

14. GENERAL CLASSIFICATION FOR SUPPLY

15. INSTRUCTIONS ON USE

16. INFORMATION IN BRAILLE

Dynastat 40 mg

17. UNIQUE IDENTIFIER – 2D BARCODE

2D barcode carrying the unique identifier included.

18. UNIQUE IDENTIFIER – HUMAN READABLE DATA

PC
SN
NN

MINIMUM PARTICULARS TO APPEAR ON SMALL IMMEDIATE PACKAGING UNITS

SOLVENT AMPOULE LABEL : 2 ml

TEXT FOR AMPOULE LABEL - EU/1/02/209/006, EU/1/02/209/007 and EU/1/02/209/008

1. NAME OF THE MEDICINAL PRODUCT AND ROUTE(S) OF ADMINISTRATION

Sodium chloride 9 mg/ml (0.9%) solution

2. METHOD OF ADMINISTRATION

Solvent for Dynastat 40 mg

Read the package leaflet before use.

3. EXPIRY DATE

EXP

4. BATCH NUMBER

Lot

5. CONTENTS BY WEIGHT, BY VOLUME OR BY UNIT

2 ml

6. OTHER

B. PACKAGE LEAFLET

Package leaflet: Information for the user

Dynastat 40 mg powder for solution for injection
parecoxib

Read all of this leaflet carefully before you start using this medicine because it contains important information for you.
- Keep this leaflet. You may need to read it again.
- If you have further questions, ask your doctor or nurse.
- If you get any side effects, talk to your doctor or nurse. This includes any possible side effects not listed in this leaflet. See section 4.

What is in this leaflet

1. What Dynastat is and what it is used for
2. What you need to know before you use Dynastat
3. How to use Dynastat
4. Possible side effects
5. How to store Dynastat
6. Contents of the pack and other information

1. What Dynastat is and what it is used for

Dynastat contains the active substance parecoxib.

Dynastat is used for the short-term treatment of pain in adults after an operation. It is one of a family of medicines called COX-2 inhibitors (this is short for *cyclo-oxygenase-2 inhibitors*). Pain and swelling are sometimes caused by substances in the body called *prostaglandins*. Dynastat works by lowering the amount of these prostaglandins.

2. What you need to know before you use Dynastat

Do not use Dynastat
- if you are allergic to parecoxib or any of the other ingredients of this medicine (listed in section 6)
- if you have had a serious allergic reaction (especially a serious skin reaction) to any medicines
- if you have had an allergic reaction to a group of medicines called “sulfonamides” (e.g. some antibiotics used to treat infections)
- if you currently have a gastric or intestinal ulcer or bleeding in the stomach or gut
- if you have had an allergic reaction to acetylsalicylic acid (aspirin) or to other NSAIDs (e.g. ibuprofen) or to COX-2 inhibitors. Reactions might include wheezing (bronchospasm), badly blocked nose, itchy skin, rash or swelling of the face, lips or tongue, other allergic reactions or nasal polyps after taking these medicines
- if you are more than 6 months pregnant
- if you are breast-feeding
- if you have severe liver disease
- if you have inflammation of the intestines (ulcerative colitis or Crohn’s disease)
- if you have heart failure
- if you are about to have heart surgery or surgery on your arteries (including any coronary artery procedure)

- if you have established heart disease and /or cerebrovascular disease e.g. if you have had a heart attack, stroke, mini-stroke (TIA) or blockages to blood vessels to the heart or brain or an operation to clear or bypass blockages
- if you have or have had problems with your blood circulation (peripheral arterial disease)

If any of these applies to you, you will not be given the injection. **Tell your doctor or nurse immediately.**

Warnings and precautions

Do not use Dynastat if you currently have a gastric or intestinal ulcer or gastrointestinal bleeding

Do not use Dynastat if you have severe liver disease

Talk to your doctor or nurse before using Dynastat:

- If you have previously had an ulcer, bleeding or perforation of the gastrointestinal tract.
- If you are taking acetylsalicylic acid (aspirin) or other NSAIDs (e.g. ibuprofen)
- If you smoke or drink alcohol
- If you have diabetes
- If you have angina, blood clots, high blood pressure or raised cholesterol
- If you are taking antiplatelet therapies
- If you have fluid retention (oedema)
- If you have liver or kidney disease.
- If you are dehydrated – this may happen if you have had diarrhoea or have been vomiting (being sick) or unable to drink fluids
- If you have an infection as it may hide a fever (which is a sign of infection)
- If you use medicines to reduce blood clotting (e.g. warfarin/warfarin like anticoagulants or novel oral anti-clotting medicines, e.g. apixaban, dabigatran, and rivaroxaban)
- If you use medicines called corticosteroids (e.g. prednisone)
- If you use a class of medicines used to treat depression called selective serotonin re-uptake inhibitors (e.g. sertraline)

Dynastat can lead to an increase in blood pressure or worsening of existing high blood pressure which may result in an increase in side effects associated with heart conditions. Your doctor may want to monitor your blood pressure during treatment with Dynastat.

Children and adolescents
Children and adolescents under the age of 18 should not be given Dynastat.

Other medicines and Dynastat
Tell your doctor or nurse if you are taking, have recently taken or might take any other medicines. Medicines can sometimes interfere with each other. Your doctor may reduce the dose of Dynastat or other medicines, or you may need to take a different medicine. It's especially important to mention:

- Acetylsalicylic acid (aspirin) or other anti-inflammatory medicines
- Fluconazole – used for fungal infections
- ACE inhibitors, Angiotensin-II inhibitors, beta blockers and diuretics – used for high blood pressure and heart conditions
- Ciclosporin or Tacrolimus – used after transplants
- Warfarin – or other warfarin like medicines used to prevent blood clots including newer medicines like apixaban, dabigatran, and rivaroxaban

- Lithium – used to treat depression
- Rifampicin – used for bacterial infections
- Antiarrhythmics – used to treat an irregular heartbeat
- Phenytoin or Carbamazepine – used for epilepsy
- Methotrexate – used for rheumatoid arthritis and cancer
- Diazepam – used for sedation and anxiety
- Omeprazole – used for treating ulcers

Pregnancy, breast-feeding and fertility

- **If you are pregnant or trying to become pregnant**, tell your doctor. Dynastat is not recommended in the first 6 months of pregnancy and you must not receive Dynastat in the last three months of pregnancy.
- **If you are breast-feeding**, you must not receive Dynastat, as a small amount of Dynastat will be transferred to your breast milk.
- NSAIDs, including Dynastat, may make it more difficult to become pregnant. You should tell your doctor if you are planning to become pregnant or if you have problems becoming pregnant.

If you are pregnant or breast-feeding, think you may be pregnant or are planning to have a baby, ask your doctor or nurse for advice before taking this medicine.

Driving and using machines

If the injection makes you feel dizzy or tired, do not drive or use machines until you feel better again.

Dynastat contains

This medicinal product contains less than 23 mg of sodium in each dose and therefore is essentially sodium-free.

3. How to use Dynastat

Dynastat will be given to you by a doctor or nurse. They will dissolve the powder before giving you the injection, and will inject the solution into a vein or a muscle. The injection may be given rapidly and directly into a vein or into an existing intravenous line (a thin tube running into a vein), or it can be given slowly and deeply into a muscle. You will only be given Dynastat for short periods, and only for pain relief.

The usual dose to start with is 40 mg.

You may be given another dose – either 20 mg or 40 mg – 6 to 12 hours after the first one.

You will not be given more than 80 mg in 24 hours.

Some people may be given lower doses:

- People with liver problems
- People with severe kidney problems
- Patients over 65 who weigh less than 50 kg
- People taking fluconazole.

If Dynastat is used with strong pain killers (called opioid analgesics) such as morphine the dose of Dynastat will be the same as explained above.

If you are given more Dynastat than you should you may experience side effects that have been reported with recommended doses.

If you have any further questions on the use of this medicine, ask your doctor or nurse.

4. Possible side effects

Like all medicines, this medicine can cause side effects, although not everybody gets them.

Stop taking Dynastat and tell your doctor immediately:
- if you develop a rash or ulceration in any part of your body (e.g. skin, mouth, eyes, face, lips or tongue), or develop any other signs of an allergic reaction such as skin rash, swelling of the face, lips or tongue which may cause wheezing, difficulty breathing, or swallowing – this occurs **rarely**
- if you have blistering or peeling of the skin - this occurs **rarely**
- the onset of skin reactions can occur at any time but most often occur in the first month of treatment; the reported rate of these events appears to be greater for valdecoxib, a medicine related to parecoxib, as compared to other COX-2 inhibitors
- if you have jaundice (your skin or the whites of your eyes appear yellow)
- if you have any signs of bleeding in the stomach or intestine, such as passing a black or blood-stained bowel movement or vomiting blood

Very common: may affect more than 1 in 10 people
- Nausea (feeling sick)

Common: may affect up to 1 in 10 people
- Change in your blood pressure (up or down)
- You may get back pain
- Ankles, legs and feet may swell (fluid retention)
- You may feel numb – your skin may lose sensitivity to pain and touch
- You may get vomiting, stomach ache, indigestion, constipation, bloating and wind
- Tests may show abnormal kidney function
- You may feel agitated or find it hard to sleep
- Dizziness
- There is a risk of anaemia - changes in red blood cells after an operation that may cause fatigue and breathlessness
- You may get a sore throat or difficulty breathing (shortness of breath)
- Your skin may be itchy
- You may pass less urine than usual.
- Dry socket (inflammation and pain after a tooth extraction)
- Increased sweating
- Low levels of potassium in blood test results

Uncommon: may affect up to 1 in 100 people
- Heart attack
- There is a risk of cerebrovascular disease e.g. stroke, or transient ischaemic attack (transient reduced blood flow to the brain)/mini-stroke or angina, or blockages to blood vessels to the heart or brain
- Blood clot in the lungs
- Worsening of high blood pressure
- Ulcers in the digestive system, chronic stomach acid reflux
- The heart may beat more slowly

- Low blood pressure on standing
- Blood tests may show abnormal liver function
- You may bruise easily due to a low blood platelet count
- Surgical wounds may become infected, abnormal discharge from surgical wounds
- Skin discolouration or bruising
- Complications with skin healing after operations
- High sugar levels in blood tests
- Injection site pain or injection site reaction
- Rash, or raised itchy rash (hives)
- Anorexia (loss of appetite)
- Joint pain
- High levels of blood enzymes in blood tests that indicate injury or stress to the heart, the brain, or muscle tissue.
- Dry mouth
- Muscle weakness
- Ear ache
- Unusual abdominal sounds

Rare: may affect up to 1 in 1,000 people
- Rash or ulceration in any part of your body (e.g. skin, mouth, eyes, face, lips or tongue), or any other signs of allergic reactions such as skin rash, swelling of the face, lips and tongue, wheezing, difficulty breathing or swallowing (potentially fatal)
- Swelling, blistering or peeling of the skin
- Acute kidney failure
- Hepatitis (inflamed liver)
- Inflammation of the gullet (oesophagus)
- Inflammation of the pancreas (can lead to stomach pain)

Not known: frequency cannot be estimated from the available data
- Collapse due to severe low blood pressure
- Heart failure
- Kidney failure
- Racing or irregularity of the heartbeat
- Breathlessness

Reporting of side effects
If you get any side effects, talk to your doctor or nurse. This includes any possible side effects not listed in this leaflet. You can also report side effects directly via the national reporting system listed in Appendix V. By reporting side effects you can help provide more information on the safety of this medicine.

5. How to store Dynastat

Keep this medicine out of the sight and reach of children.

Do not use this medicine after the expiry date which is stated on the carton and on the vial label after Exp. The expiry date refers to the last day of that month.

This medicine does not require any special storage conditions prior to reconstitution.

It is recommended that Dynastat is used as soon as possible after it is mixed with solvent, although it may be stored if the instructions at the end of the leaflet are strictly followed.

The injection solution should be a clear colourless liquid. **If there are particles** in the injection solution or if either the powder or solution is discoloured, the solution will not be used.

6. Contents of the pack and other information

What Dynastat contains

- The active substance is parecoxib (as parecoxib sodium). Each vial contains 40 mg parecoxib, as 42.36 mg parecoxib sodium. When reconstituted with 2 ml solvent, provides 20 mg/ml of parecoxib. When reconstituted in sodium chloride 9 mg/ml (0.9%) solution, Dynastat contains approximately 0.44 mEq of sodium per vial.

- The other ingredients are:

  Disodium hydrogen phosphate
  Phosphoric acid and/or sodium hydroxide (for pH adjustment).

What Dynastat looks like and contents of the pack
Dynastat is available as a white to off-white powder.
The powder is contained in colourless glass vials (5 ml) with a stopper, sealed with a purple flip-off cap on the aluminium overseal.

Marketing Authorisation Holder and Manufacturer

Marketing Authorisation Holder: Pfizer Europe MA EEIG, Boulevard de la Plaine 17, 1050 Bruxelles, Belgium
Manufacturer: Pfizer Manufacturing Belgium NV, Rijksweg 12, 2870 Puurs, Belgium

For any information about this medicinal product, please contact the local representative of the Marketing Authorisation Holder.

België/Belgique/Belgien
Pfizer S.A./ N.V.
Tél/Tel: +32 (0)2 554 62 11

България
Пфайзер Люксембург САРЛ, Клон България
Тел.: +359 2 970 4333

Česká Republika
Pfizer PFE, spol. s r.o.
Tel: +420 283 004 111

Danmark
Pfizer ApS
Tlf: +45 44 20 11 00

Deutschland
Pfizer Pharma GmbH
Tel:-+49 (0)30 550055 51000

Lietuva
Pfizer Luxembourg SARL filialas Lietuvoje.
Tel. +3705 2514000

Luxembourg/Luxemburg
Pfizer S.A.
Tél/Tel: +32 (0)2 554 62 11

Magyarország
Pfizer Kft.
Tel: + 36 1 488 37 00

Malta
Vivian Corporation Ltd.
Tel: +356 21344610

Nederland
Pfizer bv
Tel: +31 (0)10 406 4301

Eesti
Pfizer Luxembourg SARL Eesti filiaal
Tel: +372 666 7500

Ελλάδα
PFIZER ΕΛΛΑΣ A.E.,
Τηλ: +30 210 67 85 800

España
Pfizer, S.L.
Tel: +34 91 490 99 00

France
Pfizer
Tél: +33 (0)1 58 07 34 40

Hrvatska
Pfizer Croatia d.o.o.
Tel: + 385 1 3908 777

Ireland
Pfizer Healthcare Ireland
Tel: 1800 633 363 (toll free)
+ 44 (0)1304 616161

Ísland
Icepharma hf.
Sími: + 354 540 8000

Italia
Pfizer S.r.l.
Tel: +39 06 33 18 21

Κύπρος
PFIZER ΕΛΛΑΣ A.E. (Cyprus Branch)
Τηλ: +357 22 817690

Latvija
Pfizer Luxembourg SARL filiāle Latvijā
Tel: +371 670 35 775

Norge
Pfizer Norge AS
Tlf: +47 67 52 61 00

Österreich
Pfizer Corporation Austria Ges.m.b.H.
Tel: +43 (0)1 521 15-0

Polska
Pfizer Polska Sp. z o.o.,
Tel.: +48 22 335 61 00

Portugal
Pfizer Biofarmacêutica, Sociedade Unipessoal Lda
Tel: +351 21 423 5500

România
Pfizer România S.R.L.
Tel: +40 (0)21 207 28 00

Slovenija
Pfizer Luxembourg SARL
Pfizer, podružnica za svetovanje s področja farmacevtske dejavnosti, Ljubljana
Tel: + 386 (0)152 11 400

Slovenská republika
Pfizer Luxembourg SARL, organizačná zložka
Tel: +421–2–3355 5500

Suomi/Finland
Pfizer Oy
Puh/Tel: +358(0)9 43 00 40

Sverige
Pfizer Innovations AB
Tel: +46 (0)8 550 520 00

United Kingdom
Pfizer Limited
Tel: +44 (0)1304 616161

This leaflet was last revised in <{MM/YYYY}><{month YYYY}>.

<--

Detailed information on this medicine is available on the European Medicines Agency web site: http://www.ema.europa.eu.

The following information is intended for healthcare professionals only

Dosing. The recommended dose is 40 mg administered intravenously (IV) or intramuscularly (IM), followed every 6 to 12 hours by 20 mg or 40 mg as required, not to exceed 80 mg/day. The IV bolus injection may be given rapidly and directly into a vein or into an existing IV line. The IM injection should be given slowly and deeply into the muscle.

There is limited clinical experience with Dynastat treatment beyond three days.

As the cardiovascular risk of cyclooxygenase-2 (COX-2) specific inhibitors may increase with dose and duration of exposure, the shortest duration possible and the lowest effective daily dose should be used.

Cases of severe hypotension shortly following parecoxib administration have been reported in postmarketing experience with parecoxib. Some of these cases have occurred without other signs of anaphylaxis. The physician should be prepared to treat severe hypotension.

Administration is by intramuscular (IM) or intravenous (IV) injection. The IM injection is to be given slowly and deeply into the muscle and the IV bolus injection may be given rapidly and directly into a vein or into an existing IV line.

Administration other than IV or IM
Modes of administration other than IV or IM (e.g. intra-articular, intrathecal) have not been studied and should not be used.

Reconstitution solvents
This medicinal product must not be mixed with other medicinal products. It is to be reconstituted only with one of the following:

- sodium chloride 9 mg/ml (0.9%) solution for injection/infusion;
- glucose 50 mg/ml (5%) solution for infusion; or
- sodium chloride 4.5 mg/ml (0.45%) and glucose 50 mg/ml (5%) solution for injection/infusion.

The following solutions **cannot** be used for reconstitution:

- Use of Ringer-Lactate solution for injection or glucose 50 mg/ml (5%) in Ringer-Lactate solution for injection for reconstitution will cause the parecoxib to precipitate from solution and therefore is **not** recommended.
- Use of Sterile Water for Injection for reconstitution is not recommended, as the resulting solution is **not** isotonic.

Reconstitution process
Use aseptic technique to reconstitute lyophilised parecoxib (as parecoxib sodium).
40 mg vial: Remove the purple flip-off cap to expose the central portion of the rubber stopper of the parecoxib 40 mg vial. Withdraw with a sterile needle and syringe, 2 ml of an acceptable solvent and insert the needle through the central portion of the rubber stopper transferring the solvent into the parecoxib 40 mg vial.

Dissolve the powder completely using a gentle swirling motion and inspect the reconstituted product before use.

The reconstituted solution must not be used if discoloured or cloudy or if particulate matter is observed.

The entire contents of the vial should be withdrawn for a single administration. If a dose lower than 40 mg is required, excess medicine should be discarded.

IV line solution compatibility

Precipitation may occur when Dynastat is combined in solution with other medicinal products and therefore Dynastat must not be mixed with any other drug, either during reconstitution or injection. In those patients where the same IV line is to be used to inject another medicinal product, the line must be adequately flushed prior to and after Dynastat injection with a solution of known compatibility.

After reconstitution with acceptable solvents, Dynastat may only be injected IV or IM, or into IV lines delivering the following:

- sodium chloride 9 mg/ml (0.9%) solution for injection/infusion;
- glucose 50 mg/ml (5%) solution for infusion;
- sodium chloride 4.5 mg/ml (0.45%) and glucose 50 mg/ml (5%) solution for injection/infusion; or
- Ringer-Lactate solution for injection.

It is not recommended to inject into an IV line delivering glucose 50 mg/ml (5%) in Ringer-Lactate solution for injection, or other IV fluids not listed in this section, as this may cause precipitation from solution.

The solution is for single use only and must not be stored in a refrigerator or freezer.
Chemical and physical in-use stability of the reconstituted solution have been demonstrated for up to 24 hours at 25°C. Thus, 24 hours should be considered the maximum shelf life of the reconstituted product. However, due to the importance of microbiological infection risk for injectable products, the reconstituted solution should be used immediately unless reconstitution has taken place in controlled and validated aseptic conditions. Unless such requirements are met, in-storage times and conditions prior to use are the responsibility of the user, and would not normally be longer than 12 hours at 25°C.

Package leaflet: Information for the user

Dynastat 40 mg powder and solvent for solution for injection
parecoxib

Read all of this leaflet carefully before you start using this medicine because it contains important information for you.
- Keep this leaflet. You may need to read it again.
- If you have further questions, ask your doctor or nurse.
- If you get any side effects, talk to your doctor or nurse. This includes any possible side effects not listed in this leaflet. See section 4.

What is in this leaflet

1. What Dynastat is and what it is used for
2. What you need to know before you use Dynastat
3. How to use Dynastat
4. Possible side effects
5. How to store Dynastat
6. Contents of the pack and other information

1. What Dynastat is and what it is used for

Dynastat contains the active substance parecoxib.

Dynastat is used for the short-term treatment of pain in adults after an operation. It is one of a family of medicines called COX-2 inhibitors (this is short for *cyclo-oxygenase-2 inhibitors*). Pain and swelling are sometimes caused by substances in the body called *prostaglandins*. Dynastat works by lowering the amount of these prostaglandins.

2. What you need to know before you use Dynastat

Do not take Dynastat
- if you are allergic to parecoxib or any of the other ingredients of this medicine (listed in section 6)
- if you have had a serious allergic reaction (especially a serious skin reaction) to any medicines
- if you have had an allergic reaction to a group of medicines called “sulfonamides” (e.g. some antibiotics used to treat infections)
- if you currently have a gastric or intestinal ulcer or bleeding in the stomach or gut
- if you have had an allergic reaction to acetylsalicylic acid (aspirin) or to other NSAIDs (e.g. ibuprofen) or to COX-2 inhibitors. Reactions might include wheezing (bronchospasm), badly blocked nose, itchy skin, rash or swelling of the face, lips or tongue, other allergic reactions or nasal polyps after taking these medicines
- if you are more than 6 months pregnant
- if you are breast-feeding
- if you have severe liver disease
- if you have inflammation of the intestines (ulcerative colitis or Crohn’s disease)
- if you have heart failure
- if you are about to have heart surgery or surgery on your arteries (including any coronary artery procedure)

- if you have established heart disease and /or cerebrovascular disease e.g. if you have had a heart attack, stroke, mini-stroke (TIA) or blockages to blood vessels to the heart or brain or an operation to clear or bypass blockages
- if you have or have had problems with your blood circulation (peripheral arterial disease)

If any of these applies to you, you will not be given the injection. **Tell your doctor or nurse immediately.**

Warnings and precautions

Do not use Dynastat if you currently have a gastric or intestinal ulcer or gastrointestinal bleeding

Do not use Dynastat if you have severe liver disease

Talk to your doctor or nurse before using Dynastat:

- If you have previously had an ulcer, bleeding or perforation of the gastrointestinal tract
- If you are taking acetylsalicylic acid (aspirin) or other NSAIDs (e.g. ibuprofen)
- If you smoke or drink alcohol
- If you have diabetes
- If you have angina, blood clots, high blood pressure or raised cholesterol
- If you are taking antiplatelet therapies
- If you have fluid retention (oedema)
- If you have liver or kidney disease.
- If you are dehydrated – this may happen if you have had diarrhoea or have been vomiting (being sick) or unable to drink fluids
- If you have an infection as it may hide a fever (which is a sign of infection)
- If you use medicines to reduce blood clotting (e.g. warfarin/warfarin like anticoagulants or novel oral anti-clotting medicines, e.g. apixaban, dabigatran, and rivaroxaban)
- If you use medicines called corticosteroids (e.g. prednisone)
- If you use a class of medicines used to treat depression called selective serotonin re-uptake inhibitors (e.g. sertraline)

Dynastat can lead to an increase in blood pressure or worsening of existing high blood pressure which may result in an increase in side effects associated with heart conditions. Your doctor may want to monitor your blood pressure during treatment with Dynastat.

Children and adolescents
Children and adolescents under the age of 18 should not be given Dynastat.

Other medicines and Dynastat
Tell your doctor or nurse if you are taking, have recently taken or might take any other medicines. Medicines can sometimes interfere with each other. Your doctor may reduce the dose of Dynastat or other medicines, or you may need to take a different medicine. It's especially important to mention:

- Acetylsalicylic acid (aspirin) or other anti-inflammatory medicines
- Fluconazole – used for fungal infections
- ACE inhibitors, Angiotensin-II inhibitors, beta blockers and diuretics – used for high blood pressure and heart conditions
- Ciclosporin or Tacrolimus – used after transplants
- Warfarin – or other warfarin like medicines used to prevent blood clots including newer medicines like apixaban, dabigatran, and rivaroxaban

- Lithium – used to treat depression
- Rifampicin – used for bacterial infections
- Antiarrhythmics – used to treat an irregular heartbeat
- Phenytoin or Carbamazepine – used for epilepsy
- Methotrexate – used for rheumatoid arthritis and cancer
- Diazepam – used for sedation and anxiety
- Omeprazole – used for treating ulcers

Pregnancy, breast-feeding and fertility

- **If you are pregnant or trying to become pregnant**, tell your doctor. Dynastat is not recommended in the first 6 months of pregnancy and you must not receive Dynastat in the last three months of pregnancy.
- **If you are breast-feeding**, you must not receive Dynastat, as a small amount of Dynastat will be transferred to your breast milk.
- NSAIDs, including Dynastat, may make it more difficult to become pregnant. You should tell your doctor if you are planning to become pregnant or if you have problems becoming pregnant.

If you are pregnant or breast-feeding, think you may be pregnant or are planning to have a baby, ask your doctor or nurse for advice before taking this medicine.

Driving and using machines

If the injection makes you feel dizzy or tired, do not drive or use machines until you feel better again.

Dynastat contains

This medicinal product contains less than 23 mg of sodium in each dose and therefore is essentially sodium-free.

3. How to use Dynastat

Dynastat will be given to you by a doctor or nurse. They will dissolve the powder before giving you the injection, and will inject the solution into a vein or a muscle. The injection may be given rapidly and directly into a vein or into an existing intravenous line (a thin tube running into a vein), or it can be given slowly and deeply into a muscle. You will only be given Dynastat for short periods, and only for pain relief.

The usual dose to start with is 40 mg.

You may be given another dose – either 20 mg or 40 mg – 6 to 12 hours after the first one.

You will not be given more than 80 mg in 24 hours.

Some people may be given lower doses:

- People with liver problems
- People with severe kidney problems
- Patients over 65 who weigh less than 50 kg
- People taking fluconazole.

If Dynastat is used with strong pain killers (called opioid analgesics) such as morphine the dose of Dynastat will be the same as explained above.

If you are given more Dynastat than you should you may experience side effects that have been reported with recommended doses.

If you have any further questions on the use of this medicine, ask your doctor or nurse.

4. Possible side effects

Like all medicines, this medicine can cause side effects, although not everybody gets them.

Stop taking Dynastat and tell your doctor immediately:
- if you develop a rash or ulceration in any part of your body (e.g. skin, mouth, eyes, face, lips or tongue), or develop any other signs of an allergic reaction such as skin rash, swelling of the face, lips or tongue which may cause wheezing, difficulty breathing, or swallowing – this occurs **rarely**
- if you have blistering or peeling of the skin – this occurs **rarely**
- the onset of skin reactions can occur at any time but most often occur in the first month of treatment; the reported rate of these events appears to be greater for valdecoxib, a medicine related to parecoxib, as compared to other COX-2 inhibitors
- if you have jaundice (your skin or the whites of your eyes appear yellow)
- if you have any signs of bleeding in the stomach or intestine, such as passing a black or blood-stained bowel movement or vomiting blood

Very common: may affect more than 1 in 10 people
- Nausea (feeling sick)

Common: may affect up to 1 in 10 people
- Change in your blood pressure (up or down)
- You may get back pain
- Ankles, legs and feet may swell (fluid retention)
- You may feel numb – your skin may lose sensitivity to pain and touch
- You may get vomiting, stomach ache, indigestion, constipation, bloating and wind
- Tests may show abnormal kidney function
- You may feel agitated or find it hard to sleep
- Dizziness
- There is a risk of anaemia - changes in red blood cells after an operation that may cause fatigue and breathlessness
- You may get a sore throat or difficulty breathing (shortness of breath)
- Your skin may be itchy
- You may pass less urine than usual.
- Dry socket (inflammation and pain after a tooth extraction)
- Increased sweating
- Low levels of potassium in blood test results

Uncommon: may affect up to 1 in 100 people
- Heart attack
- There is a risk of cerebrovascular disease e.g. stroke, or transient ischaemic attack (transient reduced blood flow to the brain)/mini-stroke or angina, or blockages to blood vessels to the heart or brain
- Blood clot in the lungs
- Worsening of high blood pressure
- Ulcers in the digestive system, chronic stomach acid reflux
- The heart may beat more slowly
- Low blood pressure on standing

- Blood tests may show abnormal liver function
- You may bruise easily due to a low blood platelet count
- Surgical wounds may become infected, abnormal discharge from surgical wounds
- Skin discolouration or bruising
- Complications with skin healing after operations
- High sugar levels in blood tests
- Injection site pain or injection site reaction
- Rash, or raised itchy rash (hives)
- Anorexia (loss of appetite)
- Joint pain
- High levels of blood enzymes in blood tests that indicate injury or stress to the heart, the brain, or muscle tissue.
- Dry mouth
- Muscle weakness
- Ear ache
- Unusual abdominal sounds

Rare: may affect up to 1 in 1,000 people
- Rash or ulceration in any part of your body (e.g. skin, mouth, eyes, face, lips or tongue), or any other signs of allergic reactions such as skin rash, swelling of the face, lips and tongue, wheezing, difficulty breathing or swallowing (potentially fatal)
- Swelling, blistering or peeling of the skin
- Acute kidney failure
- Hepatitis (inflamed liver)
- Inflammation of the gullet (oesophagus)
- Inflammation of the pancreas (can lead to stomach pain)

Not known: frequency cannot be estimated from the available data
- Collapse due to severe low blood pressure
- Heart failure
- Kidney failure
- Racing or irregularity of the heartbeat
- Breathlessness

Reporting of side effects
If you get any side effects, talk to your doctor or nurse. This includes any possible side effects not listed in this leaflet. You can also report side effects directly via the national reporting system listed in Appendix V. By reporting side effects you can help provide more information on the safety of this medicine.

5. How to store Dynastat

Keep this medicine out of the sight and reach of children.

Do not use this medicine after the expiry date which is stated on the carton and on the vial label after Exp. The expiry date refers to the last day of that month.

This medicine does not require any special storage conditions prior to reconstitution.

It is recommended that Dynastat is used as soon as possible after it is mixed with solvent, although it may be stored if the instructions at the end of the leaflet are strictly followed.

The injection solution should be a clear colourless liquid. **If there are particles** in the injection solution or if either the powder or solution is discoloured, the solution will not be used.

6. Contents of the pack and other information

What Dynastat contains

- The active substance is parecoxib (as parecoxib sodium). Each vial contains 40 mg parecoxib, as 42.36 mg parecoxib sodium. When reconstituted with 2 ml solvent, provides 20 mg/ml of parecoxib. When reconstituted in sodium chloride 9 mg/ml (0.9%) solution, Dynastat contains approximately 0.44 mEq of sodium per vial.

- The other ingredients are:
  Powder
  Disodium hydrogen phosphate
  Phosphoric acid and/or sodium hydroxide (for pH adjustment).

  Solvent
  Sodium chloride
  Hydrochloric acid or sodium hydroxide (for pH adjustment)
  Water for injection.

What Dynastat looks like and contents of the pack
Dynastat is available as a white to off-white powder.
The powder is contained in colourless glass vials (5 ml) with a stopper, sealed with a purple flip-off cap on the aluminium overseal.
The solvent is contained in colourless neutral glass ampoules (2 ml).

Marketing Authorisation Holder and Manufacturer

Marketing Authorisation Holder: Pfizer Europe MA EEIG, Boulevard de la Plaine 17, 1050 Bruxelles, Belgium
Manufacturer: Pfizer Manufacturing Belgium NV, Rijksweg 12, 2870 Puurs, Belgium

For any information about this medicinal product, please contact the local representative of the Marketing Authorisation Holder.

België/Belgique/Belgien
Pfizer S.A./ N.V.
Tél/Tel: +32 (0)2 554 62 11

България
Пфайзер Люксембург САРЛ, Клон България
Тел.: +359 2 970 4333

Česká Republika
Pfizer PFE, spol. s r.o.
Tel: +420 283 004 111

Danmark
Pfizer ApS
Tlf: +45 44 20 11 00

Lietuva
Pfizer Luxembourg SARL filialas Lietuvoje.
Tel. +3705 2514000

Luxembourg/Luxemburg
Pfizer S.A.
Tél/Tel: +32 (0)2 554 62 11

Magyarország
Pfizer Kft.
Tel.: + 36 1 488 37 00

Malta
Vivian Corporation Ltd.
Tel: +356 21344610

Deutschland
Pfizer Pharma GmbH
Tel:+ 49 (0)30 550055 51000

Eesti
Pfizer Luxembourg SARL Eesti filiaal
Tel: +372 666 7500

Ελλάδα
PFIZER ΕΛΛΑΣ A.E.,
Τηλ: +30 210 67 85 800

España
Pfizer, S.L.
Tel: +34 91 490 99 00

France
Pfizer
Tél: +33 (0)1 58 07 34 40

Hrvatska
Pfizer Croatia d.o.o.
Tel: + 385 1 3908 777

Ireland
Pfizer Healthcare Ireland
Tel: 1800 633 363 (toll free)
+44 (0)1304 616161

Ísland
Icepharma hf.
Sími: + 354 540 8000

Italia
Pfizer S.r.l.
Tel: +39 06 33 18 21

Κύπρος
PFIZER ΕΛΛΑΣ A.E. (Cyprus Branch)
Τηλ: +357 22 817690

Latvija
Pfizer Luxembourg SARL filiāle Latvijā
Tel: +371 670 35 775

Nederland
Pfizer bv
Tel: +31 (0)10 406 4301

Norge
Pfizer Norge AS
Tlf: +47 67 52 61 00

Österreich
Pfizer Corporation Austria Ges.m.b.H.
Tel: +43 (0)1 521 15-0

Polska
Pfizer Polska Sp. z o.o.,
Tel.: +48 22 335 61 00

Portugal
Pfizer Biofarmacêutica, Sociedade Unipessoal Lda
Tel: +351 21 423 5500

România
Pfizer România S.R.L.
Tel: +40 (0)21 207 28 00

Slovenija
Pfizer Luxembourg SARL
Pfizer, podružnica za svetovanje s področja farmacevtske dejavnosti, Ljubljana
Tel: + 386 (0)152 11 400

Slovenská republika
Pfizer Luxembourg SARL, organizačná zložka
Tel: +421–2–3355 5500

Suomi/Finland
Pfizer Oy
Puh/Tel: +358(0)9 43 00 40

Sverige
Pfizer Innovations AB
Tel: +46 (0)8 550 520 00

United Kingdom
Pfizer Limited
Tel: +44 (0)1304 616161

This leaflet was last revised in <{MM/YYYY}><{month YYYY}>.
<--

Detailed information on this medicine is available on the European Medicines Agency web site: http://www.ema.europa.eu.

The following information is intended for healthcare professionals only

Dosing. The recommended dose is 40 mg administered intravenously (IV) or intramuscularly (IM), followed every 6 to 12 hours by 20 mg or 40 mg as required, not to exceed 80 mg/day. The IV bolus injection may be given rapidly and directly into a vein or into an existing IV line. The IM injection should be given slowly and deeply into the muscle.

There is limited clinical experience with Dynastat treatment beyond three days.

As the cardiovascular risk of cyclooxygenase-2 (COX-2) specific inhibitors may increase with dose and duration of exposure, the shortest duration possible and the lowest effective daily dose should be used.

Cases of severe hypotension shortly following parecoxib administration have been reported in postmarketing experience with parecoxib. Some of these cases have occurred without other signs of anaphylaxis. The physician should be prepared to treat severe hypotension.

Administration is by intramuscular (IM) or intravenous (IV) injection. The IM injection is to be given slowly and deeply into the muscle and the IV bolus injection may be given rapidly and directly into a vein or into an existing IV line.

Administration other than IV or IM
Modes of administration other than IV or IM (e.g. intra-articular, intrathecal) have not been studied and should not be used.

Reconstitution solvents
This medicinal product must not be mixed with other medicinal products. It is to be reconstituted only with one of the following:

- sodium chloride 9 mg/ml (0.9%) solution for injection/infusion;
- glucose 50 mg/ml (5%) solution for infusion; or
- sodium chloride 4.5 mg/ml (0.45%) and glucose 50 mg/ml (5%) solution for injection/infusion.

The following solutions **cannot** be used for reconstitution:

- Use of Ringer-Lactate solution for injection or glucose 50 mg/ml (5%) in Ringer-Lactate solution for injection for reconstitution will cause the parecoxib to precipitate from solution and therefore is **not** recommended.
- Use of Sterile Water for Injection for reconstitution is not recommended, as the resulting solution is **not** isotonic.

Reconstitution process
Use aseptic technique to reconstitute lyophilised parecoxib (as parecoxib sodium).
40 mg vial: Remove the purple flip-off cap to expose the central portion of the rubber stopper of the parecoxib 40 mg vial. Withdraw with a sterile needle and syringe, 2 ml of an acceptable solvent and insert the needle through the central portion of the rubber stopper transferring the solvent into the parecoxib 40 mg vial.

Dissolve the powder completely using a gentle swirling motion and inspect the reconstituted product before use.

The reconstituted solution must not be used if discoloured or cloudy or if particulate matter is observed.

The entire contents of the vial should be withdrawn for a single administration. If a dose lower than 40 mg is required, excess medicine should be discarded.

IV line solution compatibility

Precipitation may occur when Dynastat is combined in solution with other medicinal products and therefore Dynastat must not be mixed with any other drug, either during reconstitution or injection. In those patients where the same IV line is to be used to inject another medicinal product, the line must be adequately flushed prior to and after Dynastat injection with a solution of known compatibility.

After reconstitution with acceptable solvents, Dynastat may only be injected IV or IM, or into IV lines delivering the following:

- sodium chloride 9 mg/ml (0.9%) solution for injection/infusion;
- glucose 50 mg/ml (5%) solution for infusion;
- sodium chloride 4.5 mg/ml (0.45%) and glucose 50 mg/ml (5%) solution for injection/infusion; or
- Ringer-Lactate solution for injection.

It is not recommended to inject into an IV line delivering glucose 50 mg/ml (5%) in Ringer-Lactate solution for injection, or other IV fluids not listed in this section, as this may cause precipitation from solution.

The solution is for single use only and must not be stored in a refrigerator or freezer.
Chemical and physical in-use stability of the reconstituted solution have been demonstrated for up to 24 hours at 25°C. Thus, 24 hours should be considered the maximum shelf life of the reconstituted product. However, due to the importance of microbiological infection risk for injectable products, the reconstituted solution should be used immediately unless reconstitution has taken place in controlled and validated aseptic conditions. Unless such requirements are met, in-storage times and conditions prior to use are the responsibility of the user, and would not normally be longer than 12 hours at 25°C.

Celecoxib package insert

HIGHLIGHTS OF PRESCRIBING INFORMATION

These highlights do not include all the information needed to use CELEBREX safely and effectively. See full prescribing information for CELEBREX.

CELEBREX® (celecoxib) capsules
Initial U.S. Approval: 1998

WARNING: CARDIOVASCULAR AND GASTROINTESTINAL RISKS
See full prescribing information for complete boxed warning

Cardiovascular Risk

- **CELEBREX, may cause an increased risk of serious cardiovascular thrombotic events, myocardial infarction, and stroke, which can be fatal. All NSAIDs may have a similar risk. This risk may increase with duration of use. Patients with cardiovascular disease or risk factors for cardiovascular disease may be at greater risk. *(5.1,14.7)***
- **CELEBREX is contraindicated for the treatment of peri-operative pain in the setting of coronary artery bypass graft (CABG) surgery. *(4, 5.1)***

Gastrointestinal Risk

- **NSAIDs, including CELEBREX, cause an increased risk of serious gastrointestinal adverse events including bleeding, ulceration, and perforation of the stomach or intestines, which can be fatal. These events can occur at any time during use and without warning symptoms. Elderly patients are at greater risk for serious gastrointestinal (GI) events. *(5.4)***

----------------------------**INDICATIONS AND USAGE**----------------------------

CELEBREX is a nonsteroidal anti-inflammatory drug indicated for:

- Osteoarthritis (OA) (1.1)
- Rheumatoid Arthritis (RA) (1.2)
- Juvenile Rheumatoid Arthritis (JRA) in patients 2 years and older (1.3)
- Ankylosing Spondylitis (AS) (1.4)
- Acute Pain (AP) (1.5)
- Primary Dysmenorrhea (PD) (1.6)
- Familial Adenomatous Polyposis (FAP)-adjunct to usual care) (1.7)

-----------------------**DOSAGE AND ADMINISTRATION**--------------------------

Use lowest effective dose for the shortest duration consistent with treatment goals for the individual patient. (1, 5.1, 5.4)

- OA: 200 mg once daily or 100 mg twice daily (2.1, 14.1)
- RA: 100 to 200 mg twice daily (2.2, 14.2)
- JRA: 50 mg twice daily in patients 10-25 kg. 100 mg twice daily in patients more than 25 kg (2.3, 14.3)
- AS: 200 mg once daily single dose or 100 mg twice daily. If no effect is observed after 6 weeks, a trial of 400 mg (single or divided doses) may be of benefit (2.4, 14.4)
- AP and PD: 400 mg initially, followed by 200 mg dose if needed on first day. On subsequent days, 200 mg twice daily as needed (2.5, 14.5)
- FAP: 400 mg twice daily with food, as an adjunct to usual care (2.6, 14.6)

Reduce daily dose by 50% in patients with moderate hepatic impairment (Child-Pugh Class B).

Consider a dose reduction by 50% (or alternative management for JRA) in patients who are known or suspected to be CYP2C9 poor metabolizers, (2.7, 8.4, 8.8, 12.3).

---------------------**DOSAGE FORMS AND STRENGTHS**---------------------

Capsules: 50 mg, 100 mg, 200 mg and 400 mg (3)

------------------------------**CONTRAINDICATIONS**--------------------------------

- Known hypersensitivity to celecoxib or sulfonamides (4)
- History of asthma, urticaria, or other allergic-type reactions after taking aspirin or other NSAIDs (4, 5.7, 5.8, 5.13)
- Use during the perioperative period in the setting of coronary artery bypass graft (CABG) surgery (4, 5.1)

-----------------------**WARNINGS AND PRECAUTIONS**------------------------

- Serious and potentially fatal cardiovascular (CV) thrombotic events, myocardial infarction, and stroke. Patients with known CV disease/risk factors may be at greater risk (5.1, 14.7, 17.2).
- Serious gastrointestinal (GI) adverse events, which can be fatal. The risk is greater in patients with a prior history of ulcer disease or GI bleeding, and in patients at high risk for GI events, especially the elderly. CELEBREX should be used with caution in these patients (5.4, 8.5, 14.7, 17.3).
- Elevated liver enzymes and, rarely, severe hepatic reactions. Discontinue use of CELEBREX immediately if abnormal liver enzymes persist or worsen (5.5, 17.4).
- New onset or worsening of hypertension. Blood pressure should be monitored closely during treatment with CELEBREX (5.2, 7.4, 17.2).
- Fluid retention and edema. CELEBREX should be used with caution in patients with fluid retention or heart failure (5.3, 17.6).
- Renal papillary necrosis and other renal injury with long term use. Use CELEBREX with caution in the elderly, those with impaired renal function, heart failure, liver dysfunction, and those taking diuretics, ACE-inhibitors, or angiotensin II antagonists (5.6, 7.4, 8.7, 17.6).
- Anaphylactoid reactions. Do not use CELEBREX in patients with the aspirin triad (5.7, 10, 17.7).
- Serious skin adverse events such as exfoliative dermatitis, Stevens-Johnson syndrome (SJS), and toxic epidermal necrolysis (TEN), which can be fatal and can occur without warning even without known prior sulfa allergy. Discontinue CELEBREX at first appearance of rash or skin reactions (5.8, 17.5).

------------------------**ADVERSE REACTIONS**-------------------------

Most common adverse reactions in arthritis trials (>2% and >placebo): abdominal pain, diarrhea, dyspepsia, flatulence, peripheral edema, accidental injury, dizziness, pharyngitis, rhinitis, sinusitis, upper respiratory tract infection, rash (6.1).

To report SUSPECTED ADVERSE REACTIONS, contact Pfizer at 1-800-438-1985 or FDA at 1-800-FDA-1088 or www.fda.gov/medwatch

--------------------------**DRUG INTERACTIONS**-------------------------

- Concomitant use of CELEBREX and warfarin may result in increased risk of bleeding complications. (7.1)
- Concomitant use of CELEBREX increases lithium plasma levels. (7.2)
- Concomitant use of CELEBREX may reduce the antihypertensive effect of ACE Inhibitors and angiotensin II antaonists (7.4)
- Use caution with drugs known to inhibit P450 2C9 or metabolized by 2D6 due to the potential for increased plasma levels (2.7, 8.4, 8.8, 12.3)

--------------------**USE IN SPECIFIC POPULATIONS**-----------------

- Pregnancy Category C prior to 30 weeks gestation; Category D starting at 30 weeks gestation (5.9, 8.1, 17.8)

See 17 for PATIENT COUNSELING INFORMATION and Medication Guide

Revised: December 2008

FULL PRESCRIBING INFORMATION: CONTENTS*

BOXED WARNING

1. INDICATIONS AND USAGE
1.1 Osteoarthritis
1.2 Rheumatoid Arthritis
1.3 Juvenile Rheumatoid Arthritis
1.4 Ankylosing Spondylitis
1.5 Acute Pain
1.6 Primary Dysmenorrhea
1.7 Familial Adenomatous Polyposis

2. DOSAGE AND ADMINISTRATION
2.1 Osteoarthritis
2.2 Rheumatoid Arthritis
2.3 Juvenile Rheumatoid Arthritis
2.4 Ankylosing Spondylitis
2.5 Management of Acute Pain and Treatment of Primary Dysmenorrhea
2.6 Familial Adenomatous Polyposis
2.7 Special Populations

3. DOSAGE FORMS AND STRENGTHS

4. CONTRAINDICATIONS

5. WARNINGS AND PRECAUTIONS
5.1 Cardiovascular Thrombotic Events
5.2 Hypertension
5.3 Congestive Heart Failure and Edema
5.4 Gastrointestinal (GI) Effects
5.5 Hepatic Effects
5.6 Renal Effects
5.7 Anaphylactoid Reactions
5.8 Skin Reactions
5.9 Pregnancy
5.10 Corticosteroid Treatment
5.11 Hematological Effects
5.12 Disseminated Intravascular Coagulation (DIC)
5.13 Preexisting Asthma
5.14 Laboratory Tests
5.15 GI Cancer in Familial Adenomatous Polyposis
5.16 Inflammation
5.17 Concomitant NSAID Use

6. ADVERSE REACTIONS
6.1 Pre-marketing Controlled Arthritis Trials
6.2 The Celecoxib Long-Term Arthritis Safety Study
6.3 Juvenile Rheumatoid Arthritis Study
6.4 Other Pre-Approval Studies
6.5 The APC and PreSAP Trials

7. DRUG INTERACTIONS
7.1 Warfarin
7.2 Lithium
7.3 Aspirin
7.4 ACE-Inhibitors and Angiotensin II Antagonists
7.5 Fluconazole
7.6 Furosemide
7.7 Methotrexate
7.8 Concomitant NSAID Use

8. USE IN SPECIFIC POPULATIONS
8.1 Pregnancy
8.2 Labor and Delivery
8.3 Nursing Mothers
8.4 Pediatric Use
8.5 Geriatric Use
8.6 Hepatic Insufficiency
8.7 Renal Insufficiency
8.8 Poor Metabolizers of CYP2C9 Substrates

10. OVERDOSAGE

11. DESCRIPTION

12. CLINICAL PHARMACOLOGY
12.1 Mechanism of Action
12.2 Pharmacodynamics
12.3 Pharmacokinetics

13. NONCLINICAL TOXICOLOGY
13.1 Carcinogenesis, Mutagenesis, Impairment of Fertility
13.2 Animal Toxicology

14. CLINICAL STUDIES
14.1 Osteoarthritis
14.2 Rheumatoid Arthritis
14.3 Juvenile Rheumatoid Arthritis
14.4 Ankylosing Spondylitis
14.5 Analgesia, Including Primary Dysmenorrhea
14.6 Familial Adenomatous Polyposis
14.7 Special Studies

16. HOW SUPPLIED/STORAGE AND HANDLING

17. PATIENT COUNSELING INFORMATION
17.1 Medication Guide
17.2 Cardiovascular Effects
17.3 Gastrointestinal Effects
17.4 Hepatic Effects
17.5 Adverse Skin Reactions
17.6 Weight Gain and Edema
17.7 Anaphylactoid Reactions
17.8 Effects During Pregnancy
17.9 Preexisting Asthma
17.10 GI Cancer in Familial Adenomatous Polyposis

* Sections or subsections omitted from the Full Prescribing Information are not listed.

FULL PRESCRIBING INFORMATION

WARNING: CARDIOVASCULAR AND GASTROINTESTINAL RISKS

Cardiovascular Risk

- **CELEBREX may cause an increased risk of serious cardiovascular thrombotic events, myocardial infarction, and stroke, which can be fatal. All nonsteroidal anti-inflammatory drugs (NSAIDs) may have a similar risk. This risk may increase with duration of use. Patients with cardiovascular disease or risk factors for cardiovascular disease may be at greater risk. *(5.1,14.7)***

- **CELEBREX is contraindicated for the treatment of peri-operative pain in the setting of coronary artery bypass graft (CABG) surgery. *(4, 5.1)***

Gastrointestinal Risk

- **NSAIDs, including CELEBREX, cause an increased risk of serious gastrointestinal adverse events including bleeding, ulceration, and perforation of the stomach or intestines, which can be fatal. These events can occur at any time during use and without warning symptoms. Elderly patients are at greater risk for serious gastrointestinal events. *(5.4)***

1. INDICATIONS AND USAGE

Carefully consider the potential benefits and risks of CELEBREX and other treatment options before deciding to use CELEBREX. Use the lowest effective dose for the shortest duration consistent with individual patient treatment goals [*see Warnings and Precautions (5)*]

1.1 Osteoarthritis (OA)

CELEBREX is indicated for relief of the signs and symptoms of OA [*see Clinical Studies (14.1)*]

1.2 Rheumatoid Arthritis (RA)

CELEBREX is indicated for relief of the signs and symptoms of RA [*see Clinical Studies (14.2)*]

1.3 Juvenile Rheumatoid Arthritis (JRA)

CELEBREX is indicated for relief of the signs and symptoms of JRA in patients 2 years and older [*see Clinical Studies (14.3)*]

1.4 Ankylosing Spondylitis (AS)

CELEBREX is indicated for the relief of signs and symptoms of AS [*see Clinical Studies (14.4)*]

1.5 Acute Pain (AP)

CELEBREX is indicated for the management of AP in adults [*see Clinical Studies (14.5)*]

1.6 Primary Dysmenorrhea (PD)

CELEBREX is indicated for the treatment of PD [*see Clinical Studies (14.5)*]

1.7 Familial Adenomatous Polyposis (FAP)

CELEBREX is indicated to reduce the number of adenomatous colorectal polyps in FAP, as an adjunct to usual care (e.g., endoscopic surveillance, surgery). It is not known whether there is a clinical benefit from a reduction in the number of colorectal polyps in FAP patients. It is also not known whether the effects of CELEBREX treatment will persist after CELEBREX is discontinued. The efficacy and safety of CELEBREX treatment in patients with FAP beyond six months have not been studied [*see Warnings and Precautions (5.15), Clinical Studies (14.6)*]

2. DOSAGE AND ADMINISTRATION

Use lowest effective dose for the shortest duration consistent with treatment goals for the individual patient.

These doses can be given without regard to timing of meals.

2.1 Osteoarthritis

For relief of the signs and symptoms of OA the recommended oral dose is 200 mg per day administered as a single dose or as 100 mg twice daily.

2.2 Rheumatoid Arthritis

For relief of the signs and symptoms of RA the recommended oral dose is 100 to 200 mg twice daily.

2.3 Juvenile Rheumatoid Arthritis

For the relief of the signs and symptoms of JRA the recommended oral dose for pediatric patients (age 2 years and older) is based on weight. For patients ≥10 kg to ≤25 kg the recommended dose is 50 mg twice daily. For patients >25 kg the recommended dose is 100 mg twice daily.

For patients who have difficulty swallowing capsules, the contents of a CELEBREX capsule can be added to applesauce. The entire capsule contents are carefully emptied onto a level teaspoon of cool or room temperature applesauce and ingested immediately with water. The sprinkled capsule contents on applesauce are stable for up to 6 hours under refrigerated conditions (2-8° C/ 35-45° F).

2.4 Ankylosing Spondylitis

For the management of the signs and symptoms of AS, the recommended dose of CELEBREX is 200 mg daily in single (once per day) or divided (twice per day) doses. If no effect is observed after 6 weeks, a trial of 400 mg daily may be worthwhile. If no effect is observed after 6 weeks on 400 mg daily, a response is not likely and consideration should be given to alternate treatment options.

2.5 Management of Acute Pain and Treatment of Primary Dysmenorrhea

The recommended dose of CELEBREX is 400 mg initially, followed by an additional 200 mg dose if needed on the first day. On subsequent days, the recommended dose is 200 mg twice daily as needed.

2.6 Familial Adenomatous Polyposis

Usual medical care for FAP patients should be continued while on CELEBREX. To reduce the number of adenomatous colorectal polyps in patients with FAP, the recommended oral dose is 400 mg twice per day to be taken with food.

2.7 Special Populations

Hepatic insufficiency*:* The daily recommended dose of CELEBREX capsules in patients with moderate hepatic impairment (Child-Pugh Class B) should be reduced by 50%. The use of CELEBREX in patients with severe hepatic impairment is not recommended [*see Warnings and Precautions (5.5), Use in Specific Populations (8.6) and Clinical Pharmacology (12.3)].*

Poor Metabolizers of CYP2C9 Substrates*:* Patients who are known or suspected to be poor CYP2C9 metabolizers based on previous history/experience with other CYP2C9 substrates (such as warfarin, phenytoin) should be administered celecoxib with caution. Consider starting treatment at half the lowest recommended dose in poor metabolizers. Consider using alternative management in JRA patients who are poor metabolizers. [*see Use in Specific populations (8.8), and Clinical Pharmacology (12.3)]*.

3. DOSAGE FORMS AND STRENGTHS

Capsules: 50 mg, 100 mg, 200 mg and 400 mg

4. CONTRAINDICATIONS

CELEBREX is contraindicated:

- In patients with known hypersensitivity to celecoxib, aspirin, or other NSAIDs.
- In patients who have demonstrated allergic-type reactions to sulfonamides.
- In patients who have experienced asthma, urticaria, or allergic-type reactions after taking aspirin or other NSAIDs. Severe anaphylactoid reactions to NSAIDs, some of them fatal, have been reported in such patients [*see Warnings and Precautions (5.7, 5.13)*].
- For the treatment of peri-operative pain in the setting of coronary artery bypass graft (CABG) surgery [*see Warnings and Precautions (5.1)*].

5. WARNINGS AND PRECAUTIONS

5.1 Cardiovascular Thrombotic Events

Chronic use of CELEBREX may cause an increased risk of serious adverse cardiovascular thrombotic events, myocardial infarction, and stroke, which can be fatal. In the APC (Adenoma Prevention with Celecoxib) trial, the hazard ratio for the composite endpoint of cardiovascular death, MI, or stroke was 3.4 (95% CI 1.4 – 8.5) for CELEBREX 400 mg twice daily and 2.8 (95% CI 1.1 – 7.2) with CELEBREX 200 mg twice daily compared to placebo. Cumulative rates for this composite endpoint over 3 years were 3.0% (20/671 subjects) and 2.5% (17/685 subjects), respectively, compared to 0.9% (6/679 subjects) with placebo treatment. The increases in both celecoxib dose groups versus placebo-treated patients were mainly due to an increased incidence of myocardial infarction [*see Clinical Studies (14.7)*].

All NSAIDs, both COX-2 selective and non-selective, may have a similar risk. Patients with known CV disease or risk factors for CV disease may be at greater risk. To minimize the potential risk for an adverse CV event in patients treated with CELEBREX, the lowest effective dose should be used for the shortest duration consistent with individual patient treatment goals. Physicians and patients should remain alert for the development of such events, even in the absence of previous CV symptoms. Patients should be informed about the signs and/or symptoms of serious CV toxicity and the steps to take if they occur.

There is no consistent evidence that concurrent use of aspirin mitigates the increased risk of serious CV thrombotic events associated with NSAID use. The concurrent use of aspirin and CELEBREX does increase the risk of serious GI events [*see Warnings and Precautions (5.4)*].

Two large, controlled, clinical trials of a different COX-2 selective NSAID for the treatment of pain in the first 10-14 days following CABG surgery found an increased incidence of myocardial infarction and stroke [*see Contraindications (4)*].

5.2 Hypertension

As with all NSAIDs, CELEBREX can lead to the onset of new hypertension or worsening of preexisting hypertension, either of which may contribute to the increased incidence of CV events. Patients taking thiazides or loop diuretics may have impaired response to these therapies when taking NSAIDs. NSAIDs, including CELEBREX, should be used with caution in patients with hypertension. Blood pressure should be monitored closely during the initiation of therapy with CELEBREX and throughout the course of therapy. The rates of hypertension from the CLASS trial in the CELEBREX, ibuprofen and diclofenac-treated patients were 2.4%, 4.2% and 2.5%, respectively [*see Clinical Studies (14.7)*].

5.3 Congestive Heart Failure and Edema

Fluid retention and edema have been observed in some patients taking NSAIDs, including CELEBREX [*see Adverse Reactions (6.1)*]. In the CLASS study [*see Clinical Studies (14.7)*], the Kaplan-Meier cumulative rates at 9 months of peripheral edema in patients on CELEBREX 400 mg twice daily (4-fold and 2-fold the recommended OA and RA doses, respectively, and the approved dose for FAP), ibuprofen 800 mg three times daily and diclofenac 75 mg twice daily were 4.5%, 6.9% and 4.7%, respectively. CELEBREX should be used with caution in patients with fluid retention or heart failure.

5.4 Gastrointestinal (GI) Effects

Risk of GI Ulceration, Bleeding, and Perforation

NSAIDs, including CELEBREX, can cause serious gastrointestinal events including bleeding, ulceration, and perforation of the stomach, small intestine or large intestine, which can be fatal. These serious adverse events can occur at any time, with or without warning symptoms, in patients treated with NSAIDs. Only one in five patients who develop a serious upper GI adverse event on NSAID therapy is symptomatic. Complicated and symptomatic ulcer rates were 0.78% at nine months for all patients in the CLASS trial, and 2.19% for the subgroup on low-dose ASA. Patients 65 years of age and older had an incidence of 1.40% at nine months, 3.06% when also taking ASA [*see Clinical Studies (14.7)*]. With longer duration of use of NSAIDs, there is a trend for increasing the likelihood of developing a serious GI event at some time during the course of therapy. However, even short-term therapy is not without risk.

NSAIDs should be prescribed with extreme caution in patients with a prior history of ulcer disease or gastrointestinal bleeding. Patients with a prior history of peptic ulcer disease and/or gastrointestinal bleeding who use NSAIDs have a greater than 10-fold increased risk for developing a GI bleed compared to patients with neither of these risk factors. Other factors that increase the risk of GI bleeding in patients treated with NSAIDs include concomitant use of oral corticosteroids or anticoagulants, longer duration of NSAID therapy, smoking, use of alcohol, older age, and poor general health status. Most spontaneous reports of fatal GI events are in elderly or debilitated patients and therefore special care should be taken in treating this population.

To minimize the potential risk for an adverse GI event, the lowest effective dose should be used for the shortest duration consistent with individual patient treatment goals. Physicians and patients should remain alert for signs and symptoms of GI ulceration and bleeding during CELEBREX therapy and promptly initiate additional evaluation and treatment if a serious GI adverse event is suspected. For high-risk patients, alternate therapies that do not involve NSAIDs should be considered.

5.5 Hepatic Effects

Borderline elevations of one or more liver-associated enzymes may occur in up to 15% of patients taking NSAIDs, and notable elevations of ALT or AST (approximately 3 or more times the upper limit of normal) have been reported in approximately 1% of patients in clinical trials with NSAIDs. These laboratory abnormalities may progress, may remain unchanged, or may be transient with continuing therapy. Rare cases of severe hepatic reactions, including jaundice and fatal fulminant hepatitis, liver necrosis and hepatic failure (some with fatal outcome) have been reported with NSAIDs, including CELEBREX [*see Adverse Reactions (6.5)*]. In controlled clinical trials of CELEBREX, the incidence of borderline elevations (greater than or equal to 1.2 times and less than 3 times the upper limit of normal) of liver associated enzymes was 6% for CELEBREX and 5% for placebo, and approximately 0.2% of patients taking CELEBREX and 0.3% of patients taking placebo had notable elevations of ALT and AST.

A patient with symptoms and/or signs suggesting liver dysfunction, or in whom an abnormal liver test has occurred, should be monitored carefully for evidence of the development of a more severe hepatic reaction while on therapy with CELEBREX. If clinical signs and symptoms consistent with liver disease develop, or if systemic manifestations occur (e.g., eosinophilia, rash, etc.), CELEBREX should be discontinued.

5.6 Renal Effects

Long-term administration of NSAIDs has resulted in renal papillary necrosis and other renal injury. Renal toxicity has also been seen in patients in whom renal prostaglandins have a compensatory role in the maintenance of renal perfusion. In these patients, administration of an NSAID may cause a dose-dependent reduction in prostaglandin formation and, secondarily, in renal blood flow, which may precipitate overt renal decompensation. Patients at greatest risk of this reaction are those with impaired renal function, heart failure, liver dysfunction, those taking diuretics, ACE-inhibitors, angiotensin II receptor antagonists, and the elderly. Discontinuation of NSAID therapy is usually followed by recovery to the pretreatment state. Clinical trials with CELEBREX have shown renal effects similar to those observed with comparator NSAIDs.

No information is available from controlled clinical studies regarding the use of CELEBREX in patients with advanced renal disease. Therefore, treatment with CELEBREX is not

recommended in these patients with advanced renal disease. If CELEBREX therapy must be initiated, close monitoring of the patient's renal function is advisable.

5.7 Anaphylactoid Reactions

As with NSAIDs in general, anaphylactoid reactions have occurred in patients without known prior exposure to CELEBREX. In post-marketing experience, rare cases of anaphylactic reactions and angioedema have been reported in patients receiving CELEBREX. CELEBREX should not be given to patients with the aspirin triad. This symptom complex typically occurs in asthmatic patients who experience rhinitis with or without nasal polyps, or who exhibit severe, potentially fatal bronchospasm after taking aspirin or other NSAIDs [*see Contraindications (4), Warnings and Precautions (5.7)*]. Emergency help should be sought in cases where an anaphylactoid reaction occurs.

5.8 Skin Reactions

CELEBREX is a sulfonamide and can cause serious skin adverse events such as exfoliative dermatitis, Stevens-Johnson syndrome (SJS), and toxic epidermal necrolysis (TEN), which can be fatal. These serious events can occur without warning and in patients without prior known sulfa allergy. Patients should be informed about the signs and symptoms of serious skin manifestations and use of the drug should be discontinued at the first appearance of skin rash or any other sign of hypersensitivity.

5.9 Pregnancy

In late pregnancy, starting at 30 weeks gestation, CELEBREX should be avoided because it may cause premature closure of the ductus arteriosus [*see Use in Specific Populations (8.1)*].

5.10 Corticosteroid Treatment

CELEBREX cannot be expected to substitute for corticosteroids or to treat corticosteroid insufficiency. Abrupt discontinuation of corticosteroids may lead to exacerbation of corticosteroid-responsive illness. Patients on prolonged corticosteroid therapy should have their therapy tapered slowly if a decision is made to discontinue corticosteroids.

5.11 Hematological Effects

Anemia is sometimes seen in patients receiving CELEBREX. In controlled clinical trials the incidence of anemia was 0.6% with CELEBREX and 0.4% with placebo. Patients on long-term treatment with CELEBREX should have their hemoglobin or hematocrit checked if they exhibit any signs or symptoms of anemia or blood loss. CELEBREX does not generally affect platelet counts, prothrombin time (PT), or partial thromboplastin time (PTT), and does not inhibit platelet aggregation at indicated dosages [*see Clinical Pharmacology (12.2)*].

5.12 Disseminated Intravascular Coagulation (DIC)

CELEBREX should be used only with caution in pediatric patients with systemic onset JRA due to the risk of disseminated intravascular coagulation.

5.13 Preexisting Asthma

Patients with asthma may have aspirin-sensitive asthma. The use of aspirin in patients with aspirin-sensitive asthma has been associated with severe bronchospasm, which can be fatal. Since cross reactivity, including bronchospasm, between aspirin and other nonsteroidal anti-inflammatory drugs has been reported in such aspirin-sensitive patients, CELEBREX should not be administered to patients with this form of aspirin sensitivity and should be used with caution in patients with preexisting asthma.

5.14 Laboratory Tests

Because serious GI tract ulcerations and bleeding can occur without warning symptoms, physicians should monitor for signs or symptoms of GI bleeding. Patients on long-term treatment with NSAIDs should have a CBC and a chemistry profile checked periodically. If abnormal liver tests or renal tests persist or worsen, CELEBREX should be discontinued.

In controlled clinical trials, elevated BUN occurred more frequently in patients receiving CELEBREX compared with patients on placebo. This laboratory abnormality was also seen in patients who received comparator NSAIDs in these studies. The clinical significance of this abnormality has not been established.

5.15 GI Cancer in Familial Adenomatous Polyposis

Treatment with CELEBREX in FAP has not been shown to reduce the risk of gastrointestinal cancer or the need for prophylactic colectomy or other FAP-related surgeries. Therefore, the usual care of FAP patients should not be altered because of the concurrent administration of CELEBREX. In particular, the frequency of routine endoscopic surveillance should not be decreased and prophylactic colectomy or other FAP-related surgeries should not be delayed.

5.16 Inflammation

The pharmacological activity of CELEBREX in reducing inflammation, and possibly fever, may diminish the utility of these diagnostic signs in detecting infectious complications of presumed noninfectious, painful conditions.

5.17 Concomitant NSAID Use

The concomitant use of CELEBREX with any dose of a non-aspirin NSAID should be avoided due to the potential for increased risk of adverse reactions.

6. ADVERSE REACTIONS

Of the CELEBREX-treated patients in the pre-marketing controlled clinical trials, approximately 4,250 were patients with OA, approximately 2,100 were patients with RA, and approximately 1,050 were patients with post-surgical pain. More than 8,500 patients received a total daily dose of CELEBREX of 200 mg (100 mg twice daily or 200 mg once daily) or more, including more than 400 treated at 800 mg (400 mg twice daily). Approximately 3,900 patients received CELEBREX at these doses for 6 months or more; approximately 2,300 of these have received it for 1 year or more and 124 of these have received it for 2 years or more.

Because clinical trials are conducted under widely varying conditions, adverse reaction rates observed in the clinical trials of a drug cannot be directly compared to rates in the clinical trials of another drug and may not reflect the rates observed in practice. The adverse reaction information from clinical trials does, however, provide a basis for identifying the adverse events that appear to be related to drug use and for approximating rates.

6.1 Pre-marketing Controlled Arthritis Trials

Table 1 lists all adverse events, regardless of causality, occurring in ≥2% of patients receiving CELEBREX from 12 controlled studies conducted in patients with OA or RA that included a placebo and/or a positive control group. Since these 12 trials were of different durations, and patients in the trials may not have been exposed for the same duration of time, these percentages do not capture cumulative rates of occurrence.

Table 1: Adverse Events Occurring in ≥2% of CELEBREX Patients from Pre-marketing Controlled Arthritis Trials

	CBX N=4146	Placebo N=1864	NAP N=1366	DCF N=387	IBU N=345
**Gastrointestinal**					
Abdominal Pain	4.1%	2.8%	7.7%	9.0%	9.0%
Diarrhea	5.6%	3.8%	5.3%	9.3%	5.8%
Dyspepsia	8.8%	6.2%	12.2%	10.9%	12.8%
Flatulence	2.2%	1.0%	3.6%	4.1%	3.5%
Nausea	3.5%	4.2%	6.0%	3.4%	6.7%
**Body as a whole**					
Back Pain	2.8%	3.6%	2.2%	2.6%	0.9%
Peripheral Edema	2.1%	1.1%	2.1%	1.0%	3.5%
Injury-Accidental	2.9%	2.3%	3.0%	2.6%	3.2%
**Central, Peripheral Nervous system**					
Dizziness	2.0%	1.7%	2.6%	1.3%	2.3%
Headache	15.8%	20.2%	14.5%	15.5%	15.4%
**Psychiatric**					
Insomnia	2.3%	2.3%	2.9%	1.3%	1.4%
**Respiratory**					
Pharyngitis					
Rhinitis	2.3%	1.1%	1.7%	1.6%	2.6%
Sinusitis	2.0%	1.3%	2.4%	2.3%	0.6%
Upper Respiratory	5.0%	4.3%	4.0%	5.4%	5.8%
Infection	8.1%	6.7%	9.9%	9.8%	9.9%
**Skin**					
Rash	2.2%	2.1%	2.1%	1.3%	1.2%

CBX = CELEBREX 100 – 200 mg twice daily or 200 mg once daily;
NAP = Naproxen 500 mg twice daily;
DCF = Diclofenac 75 mg twice daily;
IBU = Ibuprofen 800 mg three times daily.

In placebo- or active-controlled clinical trials, the discontinuation rate due to adverse events was 7.1% for patients receiving CELEBREX and 6.1% for patients receiving placebo. Among the most common reasons for discontinuation due to adverse events in the CELEBREX treatment groups were dyspepsia and abdominal pain (cited as reasons for discontinuation in 0.8% and 0.7% of CELEBREX patients, respectively). Among patients receiving placebo, 0.6% discontinued due to dyspepsia and 0.6% withdrew due to abdominal pain.

The following adverse reactions occurred in 0.1 - 1.9% of patients treated with CELEBREX (100 - 200 mg twice daily or 200 mg once daily):

***Gastrointestinal**:* Constipation, diverticulitis, dysphagia, eructation, esophagitis, gastritis, gastroenteritis, gastroesophageal reflux, hemorrhoids, hiatal hernia, melena, dry mouth, stomatitis, tenesmus, vomiting

Cardiovascular: Aggravated hypertension, angina pectoris, coronary artery disorder, myocardial infarction

***General**:* Allergy aggravated, allergic reaction, chest pain, cyst NOS, edema generalized, face edema, fatigue, fever, hot flushes, influenza-like symptoms, pain, peripheral pain

Central, peripheral nervous system: Leg cramps, hypertonia, hypoesthesia, migraine, paresthesia, vertigo

***Hearing and vestibular**:* Deafness, tinnitus

***Heart rate and rhythm**:* Palpitation, tachycardia

***Liver and biliary**:* Hepatic function abnormal, SGOT increased, SGPT increased

***Metabolic and nutritional**:* BUN increased, CPK increased, hypercholesterolemia, hyperglycemia, hypokalemia, NPN increased, creatinine increased, alkaline phosphatase increased, weight increased

***Musculoskeletal**:* Arthralgia, arthrosis, myalgia, synovitis, tendinitis

***Platelets (bleeding or clotting)**:* Ecchymosis, epistaxis, thrombocythemia,

***Psychiatric**:* Anorexia, anxiety, appetite increased, depression, nervousness, somnolence

***Hemic**:* Anemia

***Respiratory**:* Bronchitis, bronchospasm, bronchospasm aggravated, coughing, dyspnea, laryngitis, pneumonia

***Skin and appendages**:* Alopecia, dermatitis, photosensitivity reaction, pruritus, rash erythematous, rash maculopapular, skin disorder, skin dry, sweating increased, urticaria

***Application site disorders**:* Cellulitis, dermatitis contact

***Urinary**:* Albuminuria, cystitis, dysuria, hematuria, micturition frequency, renal calculus

The following serious adverse events (causality not evaluated) occurred in <0.1% of patients (cases reported only in post-marketing experience are indicated *in italics*):

***Cardiovascular**:* Syncope, congestive heart failure, ventricular fibrillation, pulmonary embolism, cerebrovascular accident, peripheral gangrene, thrombophlebitis, *vasculitis, deep venous thrombosis*

***Gastrointestinal**:* Intestinal obstruction, intestinal perforation, gastrointestinal bleeding, colitis with bleeding, esophageal perforation, pancreatitis, ileus

***Liver and biliary**:* Cholelithiasis, *hepatitis, jaundice, liver failure*

***Hemic and lymphatic**:* Thrombocytopenia, *agranulocytosis,aplastic anemia, pancytopenia, leucopenia*

Metabolic: *Hypoglycemia, hyponatremia*

***Nervous**:* Ataxia, suicide, *aseptic meningitis, ageusia, anosmia, fatal intracranial hemorrhage [see Drug Interactions (7.1)]*

***Renal**:* Acute renal failure, *interstitial nephritis*

Skin: *Erythema multiforme, exfoliative dermatitis, Stevens-Johnson syndrome, toxic epidermal necrolysis*

***General**:* Sepsis, sudden death, *anaphylactoid reaction, angioedema*

6.2 The Celecoxib Long-Term Arthritis Safety Study [see *Special Studies* (14.7)]

***Hematological Events**:* The incidence of clinically significant decreases in hemoglobin (>2 g/dL) was lower in patients on CELEBREX 400 mg twice daily (0.5%) compared to patients on either diclofenac 75 mg twice daily (1.3%) or ibuprofen 800 mg three times daily 1.9%. The lower incidence of events with CELEBREX was maintained with or without ASA use [*see Clinical Pharmacology (12.2)*].

***Withdrawals/Serious Adverse Events**:* Kaplan-Meier cumulative rates at 9 months for withdrawals due to adverse events for CELEBREX, diclofenac and ibuprofen were 24%, 29%, and 26%, respectively. Rates for serious adverse events (i.e., causing hospitalization or felt to be life-threatening or otherwise medically significant), regardless of causality, were not different across treatment groups (8%, 7%, and 8%, respectively).

6.3 Juvenile Rheumatoid Arthritis Study

In a 12-week, double-blind, active-controlled study, 242 JRA patients 2 years to 17 years of age were treated with celecoxib or naproxen; 77 JRA patients were treated with celecoxib 3 mg/kg BID, 82 patients were treated with celecoxib 6 mg/kg BID, and 83 patients were treated with naproxen 7.5 mg/kg BID. The most commonly occurring (≥5%) adverse events in celecoxib treated patients were headache, fever (pyrexia), upper

abdominal pain, cough, nasopharyngitis, abdominal pain, nausea, arthralgia, diarrhea and vomiting. The most commonly occurring (≥5%) adverse experiences for naproxen-treated patients were headache, nausea, vomiting, fever, upper abdominal pain, diarrhea, cough, abdominal pain, and dizziness (Table 2). Compared with naproxen, celecoxib at doses of 3 and 6 mg/kg BID had no observable deleterious effect on growth and development during the course of the 12-week double-blind study. There was no substantial difference in the number of clinical exacerbations of uveitis or systemic features of JRA among treatment groups.

In a 12-week, open-label extension of the double-blind study described above, 202 JRA patients were treated with celecoxib 6 mg/kg BID. The incidence of adverse events was similar to that observed during the double-blind study; no unexpected adverse events of clinical importance emerged.

Table 2: Adverse Events Occurring in ≥5% of JRA Patients in Any Treatment Group, by System Organ Class (% of patients with events)

System Organ Class / Preferred Term	All Doses Twice Daily Celecoxib 3 mg/kg N=77	Celecoxib 6 mg/kg N=82	Naproxen 7.5 mg/kg N=83
**Any Event**	**64**	**70**	**72**
**Eye Disorders**	**5**	**5**	**5**
**Gastrointestinal**	**26**	**24**	**36**
Abdominal pain NOS	4	7	7
Abdominal pain upper	8	6	10
Vomiting NOS	3	6	11
Diarrhea NOS	5	4	8
Nausea	7	4	11
**General**	**13**	**11**	**18**
Pyrexia	8	9	11
**Infections**	**25**	**20**	**27**
Nasopharyngitis	5	6	5
**Injury and Poisoning**	**4**	**6**	**5**
**Investigations***	**3**	**11**	**7**
**Musculoskeletal**	**8**	**10**	**17**
Arthralgia	3	7	4
**Nervous System**	**17**	**11**	**21**
Headache NOS	13	10	16
Dizziness (excl vertigo)	1	1	7
**Respiratory**	**8**	**15**	**15**
Cough	7	7	8
**Skin & Subcutaneous**	**10**	**7**	**18**

* Abnormal laboratory tests, which include: Prolonged activated partial thromboplastin time, Bacteriuria NOS present, Blood creatine phosphokinase increased, Blood culture positive, Blood glucose increased, Blood pressure increased, Blood uric acid increased, Hematocrit decreased, Hematuria present, Hemoglobin decreased, Liver function tests NOS abnormal, Proteinuria present, Transaminase NOS increased, Urine analysis abnormal NOS

6.4 Other Pre-Approval Studies

Adverse Events from Ankylosing Spondylitis Studies: A total of 378 patients were treated with CELEBREX in placebo- and active-controlled AS studies. Doses up to 400 mg once daily were studied. The types of adverse events reported in the AS studies were similar to those reported in the OA/RAstudies.

Adverse Events from Analgesia and Dysmenorrhea Studies: Approximately 1,700 patients were treated with CELEBREX in analgesia and dysmenorrhea studies. All patients in post-oral surgery pain studies received a single dose of study medication. Doses up to 600 mg/day of CELEBREX were studied in primary dysmenorrhea and post-orthopedic surgery pain studies. The types of adverse events in the analgesia and dysmenorrhea studies were similar to those reported in arthritis studies. The only additional adverse event reported was post-dental extraction alveolar osteitis (dry socket) in the post-oral surgery pain studies.

Adverse Events from the Familial Adenomatous Polyposis Study: The adverse event profile reported for the 83 patients with familial adenomatous polyposis enrolled in the randomized, controlled clinical trial was similar to that reported for patients in the arthritis-controlled trials. Intestinal anastomotic ulceration was the only new adverse event reported in the FAP trial, regardless of causality, and was observed in 3 of 58 patients (one at 100 mg twice daily, and two at 400 mg twice daily) who had prior intestinal surgery.

6.5 The APC and PreSAP Trials

Adverse reactions from long-term, placebo-controlled polyp prevention studies: Exposure to CELEBREX in the APC and PreSAP trials was 400 to 800 mg daily for up to 3 years [*see Special Studies Adenomatous Polyp Prevention Studies (14.7)*].

Some adverse reactions occurred in higher percentages of patients than in the arthritis pre-marketing trials (treatment durations up to 12 weeks; see *Adverse events from* CELEBREX *pre-marketing controlled arthritis trials*, above). The adverse reactions for which these differences in patients treated with CELEBREX were greater as compared to the arthritis pre-marketing trials were as follows:

	CELEBREX (400 to 800 mg daily) N = 2285	Placebo N=1303
Diarrhea	10.5%	7.0%
Gastroesophageal reflux disease	4.7%	3.1%
Nausea	6.8%	5.3%
Vomiting	3.2%	2.1%
Dyspnea	2.8%	1.6%
Hypertension	12.5%	9.8%

The following additional adverse reactions occurred in ≥0.1% and <1% of patients taking CELEBREX, at an incidence greater than placebo in the long-term polyp prevention studies and were either not reported during the controlled arthritis pre-marketing trials or occurred with greater frequency in the long-term, placebo-controlled polyp prevention studies:

Nervous system disorders: Cerebral infarction

Eye disorders: Vitreous floaters, conjunctival hemorrhage

Ear and labyrinth: Labyrinthitis

Cardiac disorders: Angina unstable, aortic valve incompetence, coronary artery atherosclerosis, sinus bradycardia, ventricular hypertrophy

Vascular disorders: Deep vein thrombosis

Reproductive system and breast disorders: Ovarian cyst

Investigations: Blood potassium increased, blood sodium increased, blood testosterone decreased

Injury, poisoning and procedural complications: Epicondylitis, tendon rupture

7. DRUG INTERACTIONS

General: Celecoxib metabolism is predominantly mediated via cytochrome P450 (CYP) 2C9 in the liver. Co-administration of celecoxib with drugs that are known to inhibit CYP2C9 should be done with caution. Significant interactions may occur when celecoxib is administered together with drugs that inhibit CYP2C9.

In vitro studies indicate that celecoxib, although not a substrate, is an inhibitor of CYP2D6. Therefore, there is a

potential for an *in vivo* drug interaction with drugs that are metabolized by CYP2D6.

7.1 Warfarin

Anticoagulant activity should be monitored, particularly in the first few days, after initiating or changing CELEBREX therapy in patients receiving warfarin or similar agents, since these patients are at an increased risk of bleeding complications. The effect of celecoxib on the anticoagulant effect of warfarin was studied in a group of healthy subjects receiving daily 2-5 mg doses of warfarin. In these subjects, celecoxib did not alter the anticoagulant effect of warfarin as determined by prothrombin time. However, in post-marketing experience, serious bleeding events, some of which were fatal, have been reported, predominantly in the elderly, in association with increases in prothrombin time in patients receiving CELEBREX concurrently with warfarin.

7.2 Lithium

In a study conducted in healthy subjects, mean steady-state lithium plasma levels increased approximately 17% in subjects receiving lithium 450 mg twice daily with CELEBREX 200 mg twice daily as compared to subjects receiving lithium alone. Patients on lithium treatment should be closely monitored when CELEBREX is introduced or withdrawn.

7.3 Aspirin

CELEBREX can be used with low-dose aspirin. However, concomitant administration of aspirin with CELEBREX increases the rate of GI ulceration or other complications, compared to use of CELEBREX alone [*see Warnings and Precautions (5.1, 5.4) and Clinical Studies (14.7)*].
Because of its lack of platelet effects, CELEBREX is not a substitute for aspirin for cardiovascular prophylaxis [*see Clinical Pharmacology (12.2)*].

7.4 ACE-inhibitors and Angiotensin II Antagonists

Reports suggest that NSAIDs may diminish the antihypertensive effect of Angiotensin Converting Enzyme (ACE) inhibitors and angiotensin II antagonists. This interaction should be given consideration in patients taking CELEBREX concomitantly with ACE-inhibitors and angiotensin II antagonists [*see Clinical Pharmacology (12.2*].

7.5 Fluconazole

Concomitant administration of fluconazole at 200 mg once daily resulted in a two-fold increase in celecoxib plasma concentration. This increase is due to the inhibition of celecoxib metabolism via P450 2C9 by fluconazole [*see Clinical Pharmacology (12.3)*]. CELEBREX should be introduced at the lowest recommended dose in patients receiving fluconazole.

7.6 Furosemide

Clinical studies, as well as post-marketing observations, have shown that NSAIDs can reduce the natriuretic effect of furosemide and thiazides in some patients. This response has been attributed to inhibition of renal prostaglandin synthesis.

7.7 Methotrexate

In an interaction study of rheumatoid arthritis patients taking methotrexate, CELEBREX did not have an effect on the pharmacokinetics of methotrexate [*see Clinical Pharmacology (12.3)*].

7.8 Concomitant NSAID Use

The concomitant use of CELEBREX with any dose of a non-aspirin NSAID should be avoided due to the potential for increased risk of adverse reactions.

8. USE IN SPECIFIC POPULATIONS

8.1 Pregnancy

Pregnancy Category C. Pregnancy category D from 30 weeks of gestation onward.

***Teratogenic effects**:* Celecoxib at oral doses ≥150 mg/kg/day (approximately 2-fold human exposure at 200 mg twice daily as measured by AUC_{0-24}), caused an increased incidence of ventricular septal defects, a rare event, and fetal alterations, such as ribs fused, sternebrae fused and sternebrae misshapen when rabbits were treated throughout organogenesis. A dose-dependent increase in diaphragmatic hernias was observed when rats were given celecoxib at oral doses ≥30 mg/kg/day (approximately 6-fold human exposure based on the AUC_{0-24} at 200 mg twice daily) throughout organogenesis. There are no studies in pregnant women. CELEBREX should be used during pregnancy only if the potential benefit justifies the potential risk to the fetus.

Nonteratogenic effects: Celecoxib produced pre-implantation and post-implantation losses and reduced embryo/fetal survival in rats at oral dosages ≥50 mg/kg/day (approximately 6-fold human exposure based on the AUC_{0-24} at 200 mg twice daily). These changes are expected with inhibition of prostaglandin synthesis and are not the result of permanent alteration of female reproductive function, nor are they expected at clinical exposures. No studies have been conducted to evaluate the effect of celecoxib on the closure of the ductus arteriosus in humans. Therefore, use of CELEBREX during the third trimester of pregnancy should be avoided.

8.2 Labor and Delivery

Celecoxib produced no evidence of delayed labor or parturition at oral doses up to 100 mg/kg in rats (approximately 7-fold human exposure as measured by the AUC_{0-24} at 200 mg BID). The effects of CELEBREX on labor and delivery in pregnant women are unknown.

8.3 Nursing Mothers

Limited data from 3 published reports that included a total of 12 breastfeeding women showed low levels of CELEBREX in breast milk. The calculated average daily infant dose was 10-40 mcg/kg/day, less than 1% of the weight-based therapeutic dose for a two-year old-child. A report of two breastfed infants 17 and 22 months of age did not show any adverse events. Caution should be exercised when CELEBREX is administered to a nursing woman.

8.4 Pediatric Use

CELEBREX is approved for relief of the signs and symptoms of Juvenile Rheumatoid Arthritis in patients 2 years and older. Safety and efficacy have not been studied beyond six months in children. The long-term cardiovascular toxicity in children exposed to CELEBREX has not been evaluated and it is unknown if long-term risks may be similar to that seen in adults exposed to CELEBREX or other COX-2 selective and non-selective NSAIDs [*(see Boxed Warning, Warnings and Precautions (5.12), and Clinical Studies (14.3)*].

The use of celecoxib in patients 2 years to 17 years of age with pauciarticular, polyarticular course JRA or in patients with systemic onset JRA was studied in a 12-week, double-blind, active controlled, pharmacokinetic, safety and efficacy study, with a 12-week open-label extension. Celecoxib has not been studied in patients under the age of 2 years, in patients with body weight less than 10 kg (22 lbs), and in patients with active systemic features. Patients with systemic onset JRA (without active systemic features) appear to be at risk for the development of abnormal coagulation laboratory tests. In some patients with systemic onset JRA, both celecoxib and naproxen were associated with mild prolongation of activated partial thromboplastin time (APTT) but not prothrombin time (PT). NSAIDs including celecoxib should be used only with caution in patients with systemic onset JRA, due to the risk of disseminated intravascular coagulation. Patients with systemic onset JRA should be monitored for the development of abnormal coagulation tests [see *Dosage and Administration (2.3), Warnings and Precautions (5.12), Adverse Reactions (6.3), Animal Toxicology (13.2), Clinical Studies (14.3)*].

Alternative therapies for treatment of JRA should be considered in pediatric patients identified to be CYP2C9 poor metabolizers [see *Poor Metabolizers of CYP2C9 substrates (8.8)*].

8.5 Geriatric Use

Of the total number of patients who received CELEBREX in pre-approval clinical trials, more than 3,300 were 65-74 years of age, while approximately 1,300 additional patients were 75 years and over. No substantial differences in

effectiveness were observed between these subjects and younger subjects. In clinical studies comparing renal function as measured by the GFR, BUN and creatinine, and platelet function as measured by bleeding time and platelet aggregation, the results were not different between elderly and young volunteers. However, as with other NSAIDs, including those that selectively inhibit COX-2, there have been more spontaneous post-marketing reports of fatal GI events and acute renal failure in the elderly than in younger patients [*see Warnings and Precautions (5.4, 5.6)*].

8.6 Hepatic Insufficiency

The daily recommended dose of CELEBREX capsules in patients with moderate hepatic impairment (Child-Pugh Class B) should be reduced by 50%. The use of CELEBREX in patients with severe hepatic impairment is not recommended [*see Dosage and Administration (2.7) and Clinical Pharmacology (12.3)*].

8.7 Renal Insufficiency

CELEBREX is not recommended in patients with severe renal insufficiency [*see Warnings and Precautions (5.6) and Clinical Pharmacology (12.3)*].

8.8 Poor Metabolizers of CYP2C9 Substrates

Patients who are known or suspected to be poor CYP2C9 metabolizers based on previous history/experience with other CYP2C9 substrates (such as warfarin, phenytoin) should be administered celecoxib with caution. Consider starting treatment at half the lowest recommended dose. Alternative management should be considered in JRA patients identified to be CYP2C9 poor metabolizers. [*see Dosage and Administration (2.7) and Clinical Pharmacology (12.3)*].

10. OVERDOSAGE

No overdoses of CELEBREX were reported during clinical trials. Doses up to 2400 mg/day for up to 10 days in 12 patients did not result in serious toxicity. Symptoms following acute NSAID overdoses are usually limited to lethargy, drowsiness, nausea, vomiting, and epigastric pain, which are generally reversible with supportive care. Gastrointestinal bleeding can occur. Hypertension, acute renal failure, respiratory depression and coma may occur, but are rare. Anaphylactoid reactions have been reported with therapeutic ingestion of NSAIDs, and may occur following an overdose.

Patients should be managed by symptomatic and supportive care following an NSAID overdose. There are no specific antidotes. No information is available regarding the removal of celecoxib by hemodialysis, but based on its high degree of plasma protein binding (>97%) dialysis is unlikely to be useful in overdose. Emesis and/or activated charcoal (60 to 100 g in adults, 1 to 2 g/kg in children) and/or osmotic cathartic may be indicated in patients seen within 4 hours of ingestion with symptoms or following a large overdose. Forced diuresis, alkalinization of urine, hemodialysis, or hemoperfusion may not be useful due to high protein binding.

11. DESCRIPTION

CELEBREX (celecoxib) is chemically designated as 4-[5-(4-methylphenyl)- 3-(trifluoromethyl)-1H-pyrazol-1-yl] benzenesulfonamide and is a diaryl-substituted pyrazole. The empirical formula is $C_{17}H_{14}F_3N_3O_2S$, and the molecular weight is 381.38; the chemical structure is as follows:

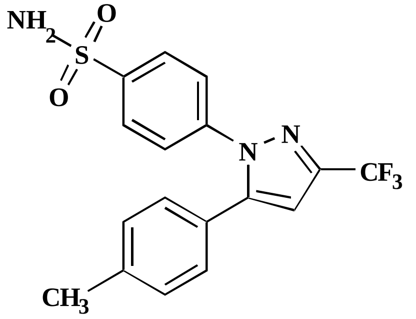

CELEBREX oral capsules contain either 50 mg, 100 mg, 200 mg or 400 mg of celecoxib, together with inactive ingredients including: croscarmellose sodium, edible inks, gelatin, lactose monohydrate, magnesium stearate, povidone and sodium lauryl sulfate.

12. CLINICAL PHARMACOLOGY

12.1 Mechanism of Action

CELEBREX is a nonsteroidal anti-inflammatory drug that exhibits anti-inflammatory, analgesic, and antipyretic activities in animal models. The mechanism of action of CELEBREX is believed to be due to inhibition of prostaglandin synthesis, primarily via inhibition of cyclooxygenase-2 (COX-2), and at therapeutic concentrations in humans, CELEBREX does not inhibit the cyclooxygenase-1 (COX-1) isoenzyme. In animal colon tumor models, CELEBREX reduced the incidence and multiplicity of tumors.

12.2 Pharmacodynamics

***Platelets**:* In clinical trials using normal volunteers, CELEBREX at single doses up to 800 mg and multiple doses of 600 mg twice daily for up to 7 days duration (higher than recommended therapeutic doses) had no effect on reduction of platelet aggregation or increase in bleeding time. Because of its lack of platelet effects, CELEBREX is not a substitute for aspirin for cardiovascular prophylaxis. It is not known if there are any effects of CELEBREX on platelets that may contribute to the increased risk of serious cardiovascular thrombotic adverse events associated with the use of CELEBREX.

***Fluid Retention**:* Inhibition of PGE2 synthesis may lead to sodium and water retention through increased reabsorption in the renal medullary thick ascending loop of Henle and perhaps other segments of the distal nephron. In the collecting ducts, PGE2 appears to inhibit water reabsorption by counteracting the action of antidiuretic hormone.

12.3 Pharmacokinetics

***Absorption**:* Peak plasma levels of celecoxib occur approximately 3 hrs after an oral dose. Under fasting conditions, both peak plasma levels (C_{max}) and area under the curve (AUC) are roughly dose-proportional up to 200 mg BID; at higher doses there are less than proportional increases in C_{max} and AUC *[see Food Effects]*. Absolute bioavailability studies have not been conducted. With multiple dosing, steady-state conditions are reached on or before Day 5. The pharmacokinetic parameters of celecoxib in a group of healthy subjects are shown in Table 3.

Table 3
Summary of Single Dose (200 mg) Disposition Kinetics of Celecoxib in Healthy Subjects[1]

Mean (%CV) PK Parameter Values				
**C_{max}, ng/mL**	**T_{max}, hr**	**Effective $t_{1/2}$, hr**	**V_{ss}/F, L**	**CL/F, L/hr**
705 (38)	2.8 (37)	11.2 (31)	429 (34)	27.7 (28)

[1] Subjects under fasting conditions (n=36, 19-52 yrs.)

***Food Effects**:* When CELEBREX capsules were taken with a high fat meal, peak plasma levels were delayed for about 1 to 2 hours with an increase in total absorption (AUC) of 10% to 20%. Under fasting conditions, at doses above 200 mg, there is less than a proportional increase in C_{max} and AUC, which is thought to be due to the low solubility of the drug in aqueous media.

Coadministration of CELEBREX with an aluminum- and magnesium-containing antacids resulted in a reduction in plasma celecoxib concentrations with a decrease of 37% in C_{max} and 10% in AUC. CELEBREX, at doses up to 200 mg twice daily, can be administered without regard to timing of meals. Higher doses (400 mg twice daily) should be administered with food to improve absorption.

In healthy adult volunteers, the overall systemic exposure (AUC) of celecoxib was equivalent when celecoxib was administered as intact capsule or capsule contents sprinkled on applesauce. There were no significant alterations in Cmax, Tmax or $t_{1/2}$ after administration of capsule contents on applesauce [*see Dosage and Administration (2)*].

***Distribution**:* In healthy subjects, celecoxib is highly protein bound (~97%) within the clinical dose range. *In vitro* studies indicate that celecoxib binds primarily to albumin and, to a lesser extent, α_1-acid glycoprotein. The apparent volume of distribution at steady state (V_{ss}/F) is approximately 400 L, suggesting extensive distribution into the tissues. Celecoxib is not preferentially bound to red blood cells.

***Metabolism**:* Celecoxib metabolism is primarily mediated via CYP2C9. Three metabolites, a primary alcohol, the corresponding carboxylic acid and its glucuronide conjugate, have been identified in human plasma. These metabolites are inactive as COX-1 or COX-2 inhibitors.

CYP2C9 activity is reduced in individuals with genetic polymorphisms that lead to reduced enzyme activity, such as those homozygous for the CYP2C9*2 and CYP2C9*3 polymorphisms. Limited data from 4 published reports that included a total of 8 subjects with the homozygous CYP2C9*3/*3 genotype showed celecoxib systemic levels that were 3- to 7-fold higher in these subjects compared to subjects with CYP2C9*1/*1 or *I/*3 genotypes. The pharmacokinetics of celecoxib have not been evaluated in subjects with other CYP2C9 polymorphisms, such as *2, *5, *6, *9 and *11. It is estimated that the frequency of the homozygous *3/*3 genotype is 0.3% to 1.0% in various ethnic groups. [*see Dosage and Administration (2.7), Use in Specific Populations (8.8)*].

***Excretion**:* Celecoxib is eliminated predominantly by hepatic metabolism with little (<3%) unchanged drug recovered in the urine and feces. Following a single oral dose of radiolabeled drug, approximately 57% of the dose was excreted in the feces and 27% was excreted into the urine. The primary metabolite in both urine and feces was the carboxylic acid metabolite (73% of dose) with low amounts of the glucuronide also appearing in the urine. It appears that the low solubility of the drug prolongs the absorption process making terminal half-life ($t_{1/2}$) determinations more variable. The effective half-life is approximately 11 hours under fasted conditions. The apparent plasma clearance (CL/F) is about 500 mL/min.

Geriatric: At steady state, elderly subjects (over 65 years old) had a 40% higher C_{max} and a 50% higher AUC compared to the young subjects. In elderly females, celecoxib C_{max} and AUC are higher than those for elderly males, but these increases are predominantly due to lower body weight in elderly females. Dose adjustment in the elderly is not generally necessary. However, for patients of less than 50 kg in body weight, initiate therapy at the lowest recommended dose [*see Dosage and Administration (2.7) and Use in Specific Populations (8.5)*].

Pediatric: The steady state pharmacokinetics of celecoxib administered as an investigational oral suspension was evaluated in 152 JRA patients 2 years to 17 years of age weighing ≥10 kg with pauciarticular or polyarticular course JRA and in patients with systemic onset JRA. Population pharmacokinetic analysis indicated that the oral clearance (unadjusted for body weight) of celecoxib increases less than proportionally to increasing weight, with 10 kg and 25 kg patients predicted to have 40% and 24% lower clearance, respectively, compared with a 70 kg adult RA patient.

Twice-daily administration of 50 mg capsules to JRA patients weighing ≥12 to ≤25 kg and 100 mg capsules to JRA patients weighing >25 kg should achieve plasma concentrations similar to those observed in a clinical trial that demonstrated the non-inferiority of celecoxib to naproxen 7.5 mg/kg twice daily (*see Dosage and Administration (2.3).* Celecoxib has not been studied in JRA patients under the age of 2 years, in patients with body weight less than 10 kg (22 lbs), or beyond 24 weeks.

***Race**:* Meta-analysis of pharmacokinetic studies has suggested an approximately 40% higher AUC of celecoxib in Blacks compared to Caucasians. The cause and clinical significance of this finding is unknown.

***Hepatic Insufficiency**:* A pharmacokinetic study in subjects with mild (Child-Pugh Class A) and moderate (Child-Pugh Class B) hepatic impairment has shown that steady-state celecoxib AUC is increased about 40% and 180%, respectively, above that seen in healthy control subjects. Therefore, the daily recommended dose of CELEBREX capsules should be reduced by approximately 50% in patients with moderate (Child-Pugh Class B) hepatic impairment. Patients with severe hepatic impairment (Child-Pugh Class C) have not been studied. The use of CELEBREX in patients with severe hepatic impairment is not recommended [*see Dosage and Administration (2.7) and Use in Specific Populations (8.6)*].

***Renal Insufficiency**:* In a cross-study comparison, celecoxib AUC was approximately 40% lower in patients with chronic renal insufficiency (GFR 35-60 mL/min) than that seen in subjects with normal renal function. No significant relationship was found between GFR and celecoxib clearance. Patients with severe renal insufficiency have not been studied. Similar to other NSAIDs, CELEBREX is not recommended in patients with severe renal insufficiency [*see Warnings and Precautions (5.6)*].

Drug interactions:

In vitro studies indicate that celecoxib is not an inhibitor of cytochrome P450 2C9, 2C19 or 3A4.

In vivo studies have shown the following:

Lithium: In a study conducted in healthy subjects, mean steady-state lithium plasma levels increased approximately 17% in subjects receiving lithium 450 mg twice daily with CELEBREX 200 mg twice daily as compared to subjects receiving lithium alone [*see Drug Interactions (7.2)*].

Fluconazole: Concomitant administration of fluconazole at 200 mg once daily resulted in a two-fold increase in celecoxib plasma concentration. This increase is due to the inhibition of celecoxib metabolism via P450 2C9 by fluconazole [*see Drug Interactions (7.5)*].

Other Drugs: The effects of celecoxib on the pharmacokinetics and/or pharmacodynamics of glyburide, ketoconazole, methotrexate [*see Drug Interactions (7.5)*], phenytoin, and tolbutamide have been studied *in vivo* and clinically important interactions have not been found.

13. NONCLINICAL TOXICOLOGY

13.1 Carcinogenesis, Mutagenesis, Impairment of Fertility

Celecoxib was not carcinogenic in rats given oral doses up to 200 mg/kg for males and 10 mg/kg for females (approximately 2-to 4-fold the human exposure as measured by the AUC_{0-24} at 200 mg twice daily) or in mice given oral doses up to 25 mg/kg for males and 50 mg/kg for females (approximately equal to human exposure as measured by the AUC_{0-24} at 200 mg twice daily) for two years.

Celecoxib was not mutagenic in an Ames test and a mutation assay in Chinese hamster ovary (CHO) cells, nor clastogenic in a chromosome aberration assay in CHO cells and an *in vivo* micronucleus test in rat bone marrow.

Celecoxib did not impair male and female fertility in rats at oral doses up to 600 mg/kg/day (approximately 11-fold human exposure at 200 mg twice daily based on the AUC_{0-24}).

13.2 Animal Toxicology

An increase in the incidence of background findings of spermatocele with or without secondary changes such as epididymal hypospermia as well as minimal to slight dilation of the seminiferous tubules was seen in the juvenile rat. These

reproductive findings while apparently treatment-related did not increase in incidence or severity with dose and may indicate an exacerbation of a spontaneous condition. Similar reproductive findings were not observed in studies of juvenile or adult dogs or in adult rats treated with celecoxib. The clinical significance of this observation is unknown.

14. CLINICAL STUDIES

14.1 Osteoarthritis

CELEBREX has demonstrated significant reduction in joint pain compared to placebo. CELEBREX was evaluated for treatment of the signs and the symptoms of OA of the knee and hip in placebo- and active-controlled clinical trials of up to 12 weeks duration. In patients with OA, treatment with CELEBREX 100 mg twice daily or 200 mg once daily resulted in improvement in WOMAC (Western Ontario and McMaster Universities) osteoarthritis index, a composite of pain, stiffness, and functional measures in OA. In three 12-week studies of pain accompanying OA flare, CELEBREX doses of 100 mg twice daily and 200 mg twice daily provided significant reduction of pain within 24-48 hours of initiation of dosing. At doses of 100 mg twice daily or 200 mg twice daily the effectiveness of CELEBREX was shown to be similar to that of naproxen 500 mg twice daily. Doses of 200 mg twice daily provided no additional benefit above that seen with 100 mg twice daily. A total daily dose of 200 mg has been shown to be equally effective whether administered as 100 mg twice daily or 200 mg once daily.

14.2 Rheumatoid Arthritis

CELEBREX has demonstrated significant reduction in joint tenderness/pain and joint swelling compared to placebo. CELEBREX was evaluated for treatment of the signs and symptoms of RA in placebo- and active-controlled clinical trials of up to 24 weeks in duration. CELEBREX was shown to be superior to placebo in these studies, using the ACR20 Responder Index, a composite of clinical, laboratory, and functional measures in RA. CELEBREX doses of 100 mg twice daily and 200 mg twice daily were similar in effectiveness and both were comparable to naproxen 500 mg twice daily.

Although CELEBREX 100 mg twice daily and 200 mg twice daily provided similar overall effectiveness, some patients derived additional benefit from the 200 mg twice daily dose. Doses of 400 mg twice daily provided no additional benefit above that seen with 100-200 mg twice daily.

14.3 Juvenile Rheumatoid Arthritis

In a 12-week, randomized, double-blind active-controlled, parallel-group, multicenter, non-inferiority study, patients from 2 years to 17 years of age with pauciarticular, polyarticular course JRA or systemic onset JRA (with currently inactive systemic features), received one of the following treatments: celecoxib 3 mg/kg (to a maximum of 150 mg) twice daily; celecoxib 6 mg/kg (to a maximum of 300 mg) twice daily; or naproxen 7.5 mg/kg (to a maximum of 500 mg) twice daily. The response rates were based upon the JRA Definition of Improvement greater than or equal to 30% (JRA DOI 30) criterion, which is a composite of clinical, laboratory, and functional measures of JRA. The JRA DOI 30 response rates at week 12 were 69%, 80% and 67% in the celecoxib 3 mg/kg BID, celecoxib 6 mg/kg BID, and naproxen 7.5 mg/kg BID treatment groups, respectively.

The efficacy and safety of CELEBREX for JRA have not been studied beyond six months. The long-term cardiovascular toxicity in children exposed to CELEBREX has not been evaluated and it is unknown if the long-term risk may be similar to that seen in adults exposed to CELEBREX or other COX-2 selective and non-selective NSAIDs [(*see Boxed Warning, Warnings and Precautions (5.12)*].

14.4 Ankylosing Spondylitis

CELEBREX was evaluated in AS patients in two placebo- and active-controlled clinical trials of 6 and 12 weeks duration. CELEBREX at doses of 100 mg twice daily, 200 mg once daily and 400 mg once daily was shown to be statistically superior to placebo in these studies for all three co-primary efficacy measures assessing global pain intensity (Visual Analogue Scale), global disease activity (Visual Analogue Scale) and functional impairment (Bath Ankylosing Spondylitis Functional Index). In the 12-week study, there was no difference in the extent of improvement between the 200 mg and 400 mg CELEBREX doses in a comparison of mean change from baseline, but there was a greater percentage of patients who responded to CELEBREX 400 mg, 53%, than to CELEBREX 200 mg, 44%, using the Assessment in Ankylosing Spondylitis response criteria (ASAS 20). The ASAS 20 defines a responder as improvement from baseline of at least 20% and an absolute improvement of at least 10 mm, on a 0 to 100 mm scale, in at least three of the four following domains: patient global pain, Bath Ankylosing Spondylitis Functional Index, and inflammation. The responder analysis also demonstrated no change in the responder rates beyond 6 weeks.

14.5 Analgesia, including Primary Dysmenorrhea

In acute analgesic models of post-oral surgery pain, post-orthopedic surgical pain, and primary dysmenorrhea, CELEBREX relieved pain that was rated by patients as moderate to severe. Single doses [*see Dosage and Administration (2.5)*] of CELEBREX provided pain relief within 60 minutes.

14.6 Familial Adenomatous Polyposis

CELEBREX was evaluated to reduce the number of adenomatous colorectal polyps. A randomized, double-blind, placebo-controlled study was conducted in patients with FAP. The study population included 58 patients with a prior subtotal or total colectomy and 25 patients with an intact colon. Thirteen patients had the attenuated FAP phenotype.

One area in the rectum and up to four areas in the colon were identified at baseline for specific follow-up, and polyps were counted at baseline and following six months of treatment. The mean reduction in the number of colorectal polyps was 28% for CELEBREX 400 mg twice daily, 12% for CELEBREX 100 mg twice daily and 5% for placebo. The reduction in polyps observed with CELEBREX 400 mg twice daily was statistically superior to placebo at the six-month timepoint (p=0.003). (See Figure 1)

Figure 1
Percent Change from Baseline in Number of Colorectal Polyps (FAP Patients)

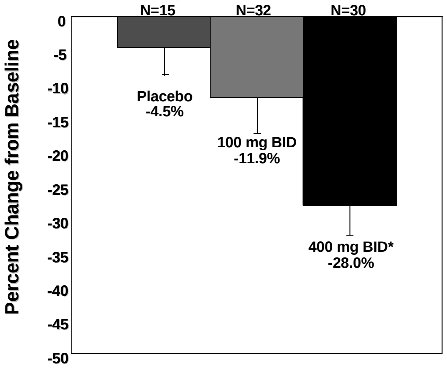

* p=0.003 versus placebo

14.7 Special Studies

Adenomatous Polyp Prevention Studies:
Cardiovascular safety was evaluated in two randomized, double-blind, placebo-controlled, three year studies involving patients with Sporadic Adenomatous Polyps treated with CELEBREX: the APC trial (Adenoma Prevention with Celecoxib) and the PreSAP trial (Prevention of Spontaneous Adenomatous Polyps). In the APC trial, there was a dose-related increase in the composite endpoint (adjudicated) of cardiovascular death, myocardial infarction, or stroke with celecoxib compared to placebo over 3 years of treatment. The PreSAP trial did not demonstrate a statistically significant increased risk for the same composite endpoint (adjudicated):

- In the APC trial, the hazard ratios compared to placebo for a composite endpoint (adjudicated) of cardiovascular death, myocardial infarction, or stroke were 3.4 (95% CI 1.4 - 8.5) with celecoxib 400 mg twice daily and 2.8 (95% CI 1.1 - 7.2) with celecoxib 200 mg twice daily. Cumulative rates for this composite endpoint over 3 years were 3.0% (20/671 subjects) and 2.5% (17/685 subjects), respectively, compared to 0.9% (6/679 subjects) with placebo treatment. The increases in both celecoxib dose groups versus placebo-treated patients were mainly due to an increased incidence of myocardial infarction.
- In the PreSAP trial, the hazard ratio for this same composite endpoint (adjudicated) was 1.2 (95% CI 0.6 - 2.4) with celecoxib 400 mg once daily compared to placebo. Cumulative rates for this composite endpoint over 3 years were 2.3% (21/933 subjects) and 1.9% (12/628 subjects), respectively.

Clinical trials of other COX-2 selective and non-selective NSAIDs of up to three-years duration have shown an increased risk of serious cardiovascular thrombotic events, myocardial infarction, and stroke, which can be fatal. As a result, all NSAIDs are considered potentially associated with this risk.

Celecoxib Long-Term Arthritis Safety Study (CLASS):
This was a prospective, long-term, safety outcome study conducted post-marketing in approximately 5,800 OA patients and 2,200 RA patients. Patients received CELEBREX 400 mg twice daily (4-fold and 2-fold the recommended OA and RA doses, respectively, and the approved dose for FAP), ibuprofen 800 mg three times daily or diclofenac 75 mg twice daily (common therapeutic doses). Median exposures for CELEBREX (n = 3,987) and diclofenac (n = 1,996) were 9 months while ibuprofen (n = 1,985) was 6 months. The primary endpoint of this outcome study was the incidence of complicated ulcers (gastrointestinal bleeding, perforation or obstruction). Patients were allowed to take concomitant low-dose (≤ 325 mg/day) aspirin (ASA) for cardiovascular prophylaxis (ASA subgroups: CELEBREX, n = 882; diclofenac, n = 445; ibuprofen, n = 412). Differences in the incidence of complicated ulcers between CELEBREX and the combined group of ibuprofen and diclofenac were not statistically significant.

Patients on CELEBREX and concomitant low-dose ASA (N=882) experienced 4-fold higher rates of complicated ulcers compared to those not on ASA (N=3105). The Kaplan-Meier rate for complicated ulcers at 9 months was 1.12% versus 0.32% for those on low-dose ASA and those not on ASA, respectively [*see Warnings and Precautions (5.4)*].

The estimated cumulative rates at 9 months of complicated and symptomatic ulcers for patients treated with CELEBREX 400 mg twice daily are described in Table 4. Table 4 also displays results for patients less than or greater than 65 years of age. The difference in rates between CELEBREX alone and CELEBREX with ASA groups may be due to the higher risk for GI events in ASA users.

Table 4: Complicated and Symptomatic Ulcer Rates in Patients Taking CELEBREX 400 mg Twice Daily (Kaplan-Meier Rates at 9 months [%]) Based on Risk Factors

**All Patients**	
CELEBREX alone (n=3105)	0.78
CELEBREX with ASA (n=882)	2.19
**Patients <65 Years**	
CELEBREX alone (n=2025)	0.47
CELEBREX with ASA (n=403)	1.26
**Patients ≥65 Years**	
CELEBREX alone (n=1080)	1.40
CELEBREX with ASA (n=479)	3.06

In a small number of patients with a history of ulcer disease, the complicated and symptomatic ulcer rates in patients taking CELEBREX alone or CELEBREX with ASA were, respectively, 2.56% (n=243) and 6.85% (n=91) at 48 weeks. These results are to be expected in patients with a prior history of ulcer disease [*see Warnings and Precautions (5.4) and Adverse Reactions (6.1)*].

Cardiovascular safety outcomes were also evaluated in the CLASS trial. Kaplan-Meier cumulative rates for investigator-reported serious cardiovascular thromboembolic adverse events (including MI, pulmonary embolism, deep venous thrombosis, unstable angina, transient ischemic attacks, and ischemic cerebrovascular accidents) demonstrated no differences between the CELEBREX, diclofenac, or ibuprofen treatment groups. The cumulative rates in all patients at nine months for CELEBREX, diclofenac, and ibuprofen were 1.2%, 1.4%, and 1.1%, respectively. The cumulative rates in non-ASA users at nine months in each of the three treatment groups were less than 1%. The cumulative rates for myocardial infarction in non-ASA users at nine months in each of the three treatment groups were less than 0.2%. There was no placebo group in the CLASS trial, which limits the ability to determine whether the three drugs tested had no increased risk of CV events or if they all increased the risk to a similar degree.

Endoscopic Studies: The correlation between findings of short-term endoscopic studies with CELEBREX and the relative incidence of clinically significant serious upper GI events with long-term use has not been established. Serious clinically significant upper GI bleeding has been observed in patients receiving CELEBREX in controlled and open-labeled trials [*see Warnings and Precautions (5.4) and Clinical Studies (14.7)*]

A randomized, double-blind study in 430 RA patients was conducted in which an endoscopic examination was performed at 6 months. The incidence of endoscopic ulcers in patients taking CELEBREX 200 mg twice daily was 4% vs. 15% for patients taking diclofenac SR 75 mg twice daily. However, CELEBREX was not statistically different than diclofenac for clinically relevant GI outcomes in the CLASS trial [*see Clinical Studies (14.7)*].

The incidence of endoscopic ulcers was studied in two 12-week, placebo-controlled studies in 2157 OA and RA patients in whom baseline endoscopies revealed no ulcers. There was no dose relationship for the incidence of gastroduodenal ulcers and the dose of CELEBREX (50 mg to 400 mg twice daily). The incidence for naproxen 500 mg twice daily was 16.2 and 17.6% in the two studies, for placebo was 2.0 and 2.3%, and for all doses of CELEBREX the incidence ranged between 2.7%-5.9%. There have been no large, clinical outcome studies to compare clinically relevant GI outcomes with CELEBREX and naproxen.

In the endoscopic studies, approximately 11% of patients were taking aspirin (≤ 325 mg/day). In the CELEBREX groups, the endoscopic ulcer rate appeared to be higher in aspirin users than in non-users. However, the increased rate of ulcers in these aspirin users was less than the endoscopic ulcer rates observed in the active comparator groups, with or without aspirin.

16. HOW SUPPLIED/STORAGE AND HANDLING

CELEBREX 50 mg capsules are white, with reverse printed white on red band of body and cap with markings of 7767 on the cap and 50 on the body, supplied as:

NDC Number	Size

0025-1515-01	bottle of 60

CELEBREX 100 mg capsules are white, with reverse printed white on blue band of body and cap with markings of 7767 on the cap and 100 on the body, supplied as:

NDC Number	Size
0025-1520-31	bottle of 100
0025-1520-51	bottle of 500
0025-1520-34	carton of 100 unit dose

CELEBREX 200 mg capsules are white, with reverse printed white on gold band with markings of 7767 on the cap and 200 on the body, supplied as:

NDC Number	Size
0025-1525-31	bottle of 100
0025-1525-51	bottle of 500
0025-1525-34	carton of 100 unit dose

CELEBREX 400 mg capsules are white, with reverse printed white on green band with markings of 7767 on the cap and 400 on the body, supplied as:

NDC Number	Size
0025-1530-02	bottle of 60
0025-1530-01	carton of 100 unit dose

Storage*:* Store at 25°C (77°F); excursions permitted to 15-30°C (59-86°F) [see USP Controlled Room Temperature]

17. PATIENT COUNSELING INFORMATION

Patients should be informed of the following information before initiating therapy with CELEBREX and periodically during the course of ongoing therapy.

17.1 Medication Guide

Patients should be informed of the availability of a Medication Guide for NSAIDs that accompanies each prescription dispensed, and should be instructed to read the Medication Guide prior to using CELEBREX.

17.2 Cardiovascular Effects

Patients should be informed that CELEBREX may cause serious CV side effects such as MI or stroke, which may result in hospitalization and even death. Patients should be informed of the the signs and symptoms of chest pain, shortness of breath, weakness, slurring of speech, and to seek immediate medical advice if they observe any of these signs or symptoms. [*see Warnings and Precautions (5.1)*].

Patients should be informed that CELEBREX can lead to the onset of new hypertension or worsening of preexisting hypertension, and that CELEBREX may impair the response of some antihypertensive agents. Patients should be instructed on the proper follow up for monitoring of blood pressure. [*see Warnings and Precautions (5.2) and Drug Interactions (7.4)*].

17.3 Gastrointestinal Effects

Patients should be informed that CELEBREX can cause gastrointestinal discomfort and more serious side effects, such as ulcers and bleeding, which may result in hospitalization and even death. Patients should be informed of the signs and symptoms of ulcerations and bleeding, and to seek immediate medical advice if they observe any signs or symptoms that are indicative of these disorders, including epigastric pain, dyspepsia, melena, and hematemesis. [*see Warnings and Precautions (5.4)*].

17.4 Hepatic Effects

Patients should be informed of the warning signs and symptoms of hepatotoxicity (e.g., nausea, fatigue, lethargy, pruritus, jaundice, right upper quadrant tenderness, and “flu-like” symptoms). Patients should be instructed that they should stop therapy and seek immediate medical therapy if these signs and symptoms occur [*see Warnings and Precautions (5.5), Use in Specific Populations (8.6)*].

17.5 Adverse Skin Reactions

Patients should be informed that CELEBREX is a sulfonamide and can cause serious skin side effects such as exfoliative dermatitis, SJS, and TEN, which may result in hospitalizations and even death. Although serious skin reactions may occur without warning, patients should be informed of the signs and symptoms of skin rash and blisters, fever, or other signs of hypersensitivity such as itching, and seek immediate medical advice when observing any indicative signs or symptoms.

Patients should be advised to stop CELEBREX immediately if they develop any type of rash and contact their physician as soon as possible.

Patients with prior history of sulfa allergy should not take CELEBREX [*see Warnings and Precautions (5.8)*].

17.6 Weight Gain and Edema

Long-term administration of NSAIDs including CELEBREX has resulted in renal injury. Patients at greatest risk are those taking diuretics, ACE-inhibitors, angiotensin II antagonists, or with renal or liver dysfunction, heart failure, and the elderly [*see Warnings and Precautions (5.3, 5.6), Use in Specific Populations (8*].

Patients should be instructed to promptly report to their physicians signs or symptoms of unexplained weight gain or edema following treatment with CELEBREX [*see Warnings and Precautions (5.3)*].

17.7 Anaphylactoid Reactions

Patients should be informed of the signs and symptoms of an anaphylactoid reaction (e.g., difficulty breathing, swelling of the face or throat). Patients should be instructed to seek immediate emergency assistance if they develop any of these signs and symptoms [*see Warnings and Precautions (5.7)*].

17.8 Effects During Pregnancy

Patients should be informed that in late pregnancy CELEBREX should be avoided because it may cause premature closure of the ductus arteriosus [*see Warnings and Precautions (5.9), Use in Specific Populations (8.1)*].

17.9 Preexisting Asthma

Patients should be instructed to tell their physicians if they have a history of asthma or aspirin-sensitive asthma because the use of NSAIDs in patients with aspirin-sensitive asthma has been associated with severe bronchospasm, which can be fatal. Patients with this form of aspirin sensitivity should be instructed not to take Celebrex. Patients with preexisting asthma should be instructed to seek immediate medical attention if their asthma worsens after taking Celebrex [*see Warnings and Precautions (5.13)*].

17.10 GI Cancer in Familial Adenomatous Polyposis

Patients with FAP should be informed that CELEBREX has not been shown to reduce colorectal, duodenal or other FAP-related cancers, or the need for endoscopic surveillance, prophylactic or other FAP-related surgery. Therefore, all patients with FAP should be instructed to continue their usual care while receiving CELEBREX [*see Warnings and Precautions (5.15)*].

Distributed by
G.D. Searle LLC
Division of Pfizer Inc, NY, NY 10017

LAB-0036-12
December 2008

Medication Guide
for
Non-Steroidal Anti-Inflammatory Drugs (NSAIDs)
(See the end of this Medication Guide for a list of prescription NSAID medicines.)

What is the most important information I should know about medicines called Non-Steroidal Anti-Inflammatory Drugs (NSAIDs)?

NSAID medicines may increase the chance of a heart attack or stroke that can lead to death. This chance increases:
- with longer use of NSAID medicines
    - in people who have heart disease

NSAID medicines should never be used right before or after a heart surgery called a "coronary artery bypass graft (CABG)."

NSAID medicines can cause ulcers and bleeding in the stomach and intestines at any time during treatment. Ulcers and bleeding:
- can happen without warning symptoms
- may cause death

The chance of a person getting an ulcer or bleeding increases with:
- taking medicines called "corticosteroids" and "anticoagulants"
- longer use
- smoking
- drinking alcohol
- older age
- having poor health

NSAID medicines should only be used:
- exactly as prescribed
- at the lowest dose possible for your treatment
- for the shortest time needed

What are Non-Steroidal Anti-Inflammatory Drugs (NSAIDs)?
NSAID medicines are used to treat pain and redness, swelling, and heat (inflammation) from medical conditions such as:
- different types of arthritis
- menstrual cramps and other types of short-term pain

Who should not take a Non-Steroidal Anti-Inflammatory Drug (NSAID)?
Do not take an NSAID medicine:
- if you had an asthma attack, hives, or other allergic reaction with aspirin or any other NSAID medicine
- for pain right before or after heart bypass surgery

Tell your healthcare provider:
- about all of your medical conditions.
- about all of the medicines you take. NSAIDs and some other medicines can interact with each other and cause serious side effects. **Keep a list of your medicines to show to your healthcare provider and pharmacist.**
- if you are pregnant. **NSAID medicines should not be used by pregnant women late in their**

pregnancy.
- if you are breastfeeding. **Talk to your doctor.**

What are the possible side effects of Non-Steroidal Anti-Inflammatory Drugs (NSAIDs)?

Serious side effects include:	Other side effects include:
• heart attack<br>• stroke<br>• high blood pressure<br>• heart failure from body swelling (fluid retention)<br>• kidney problems including kidney failure<br>• bleeding and ulcers in the stomach and intestine<br>• low red blood cells (anemia)<br>• life-threatening skin reactions<br>• life-threatening allergic reactions<br>• liver problems including liver failure<br>• asthma attacks in people who have asthma	• stomach pain<br>• constipation<br>• diarrhea<br>• gas<br>• heartburn<br>• nausea<br>• vomiting<br>• dizziness

Get emergency help right away if you have any of the following symptoms:
- shortness of breath or trouble breathing
- chest pain
- weakness in one part or side of your body
- slurred speech
- swelling of the face or throat

Stop your NSAID medicine and call your healthcare provider right away if you have any of the following symptoms:
- nausea
- more tired or weaker than usual
- itching
- your skin or eyes look yellow
- stomach pain
- flu-like symptoms
- vomit blood
- there is blood in your bowel movement or it is black and sticky like tar
- skin rash or blisters with fever
- unusual weight gain
- swelling of the arms and legs, hands and feet

These are not all the side effects with NSAID medicines. Talk to your healthcare provider or pharmacist for more information about NSAID medicines.

Other information about Non-Steroidal Anti-Inflammatory Drugs (NSAIDs)
- Aspirin is an NSAID medicine but it does not increase the chance of a heart attack. Aspirin can cause bleeding in the brain, stomach, and intestines. Aspirin can also cause ulcers in the stomach and intestines.

- Some of these NSAID medicines are sold in lower doses without a prescription (over –the –counter). Talk to your healthcare provider before using over –the –counter NSAIDs for more than 10 days.

NSAID medicines that need a prescription

Generic Name	Tradename
Celecoxib	Celebrex
Diclofenac	Cataflam, Voltaren, Arthrotec (combined with misoprostol)
Diflunisal	Dolobid
Etodolac	Lodine, Lodine XL
Fenoprofen	Nalfon, Nalfon 200
Flurbiprofen	Ansaid
Ibuprofen	Motrin, Tab-Profen, Vicoprofen* (combined with hydrocodone), Combunox (combined with oxycodone)
Indomethacin	Indocin, Indocin SR, Indo-Lemmon, Indomethagan
Ketoprofen	Oruvail
Ketorolac	Toradol
Mefenamic Acid	Ponstel
Meloxicam	Mobic
Nabumetone	Relafen
Naproxen	Naprosyn, Anaprox, Anaprox DS, EC-Naproxyn, Naprelan, Naprapac (copackaged with lansoprazole)
Oxaprozin	Daypro
Piroxicam	Feldene
Sulindac	Clinoril
Tolmetin	Tolectin, Tolectin DS, Tolectin 600

* Vicoprofen contains the same dose of ibuprofen as over-the-counter (OTC) NSAIDs, and is usually used for less than 10 days to treat pain. The OTC NSAID label warns that long term continuous use may increase the risk of heart attack or stroke.

This Medication Guide has been approved by the U.S. Food and Drug Administration.
